# Supplementary figures and images for: Metagenomics of African Empogona and Tricalysia (Rubiaceae) reveals the presence of leaf endophytes
Source: PeerJ. 2023 Aug 4;11:e15778. doi: 10.7717/peerj.15778 (PMC10405798; doi:10.7717/peerj.15778)

**A**

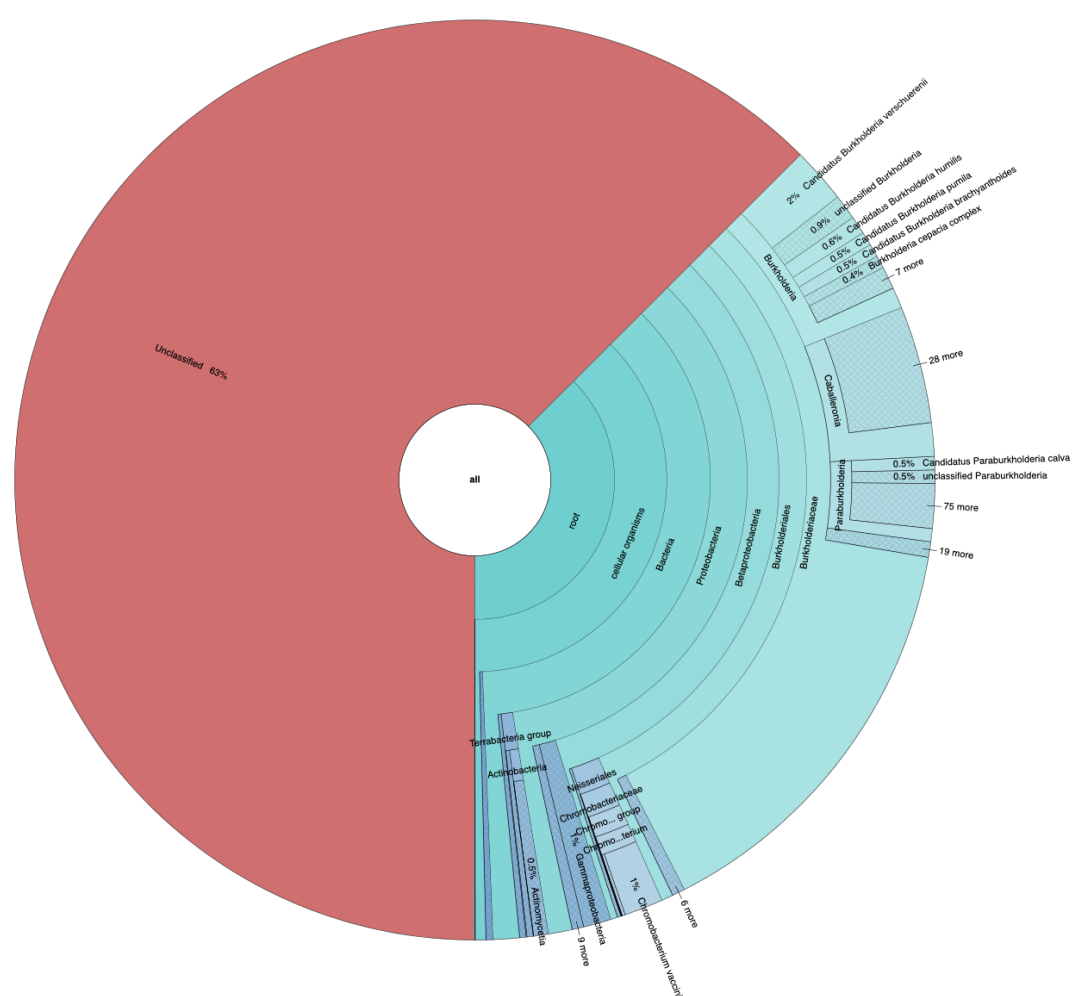

# B

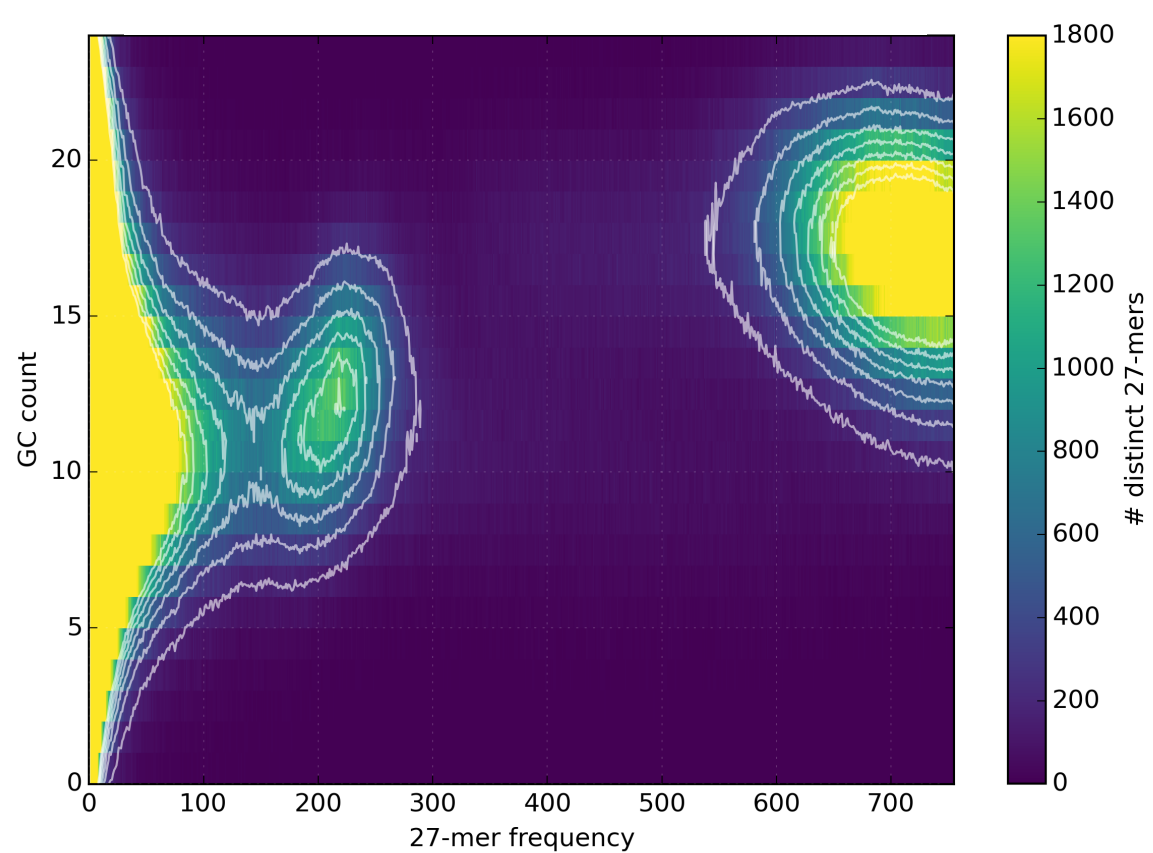

**C**

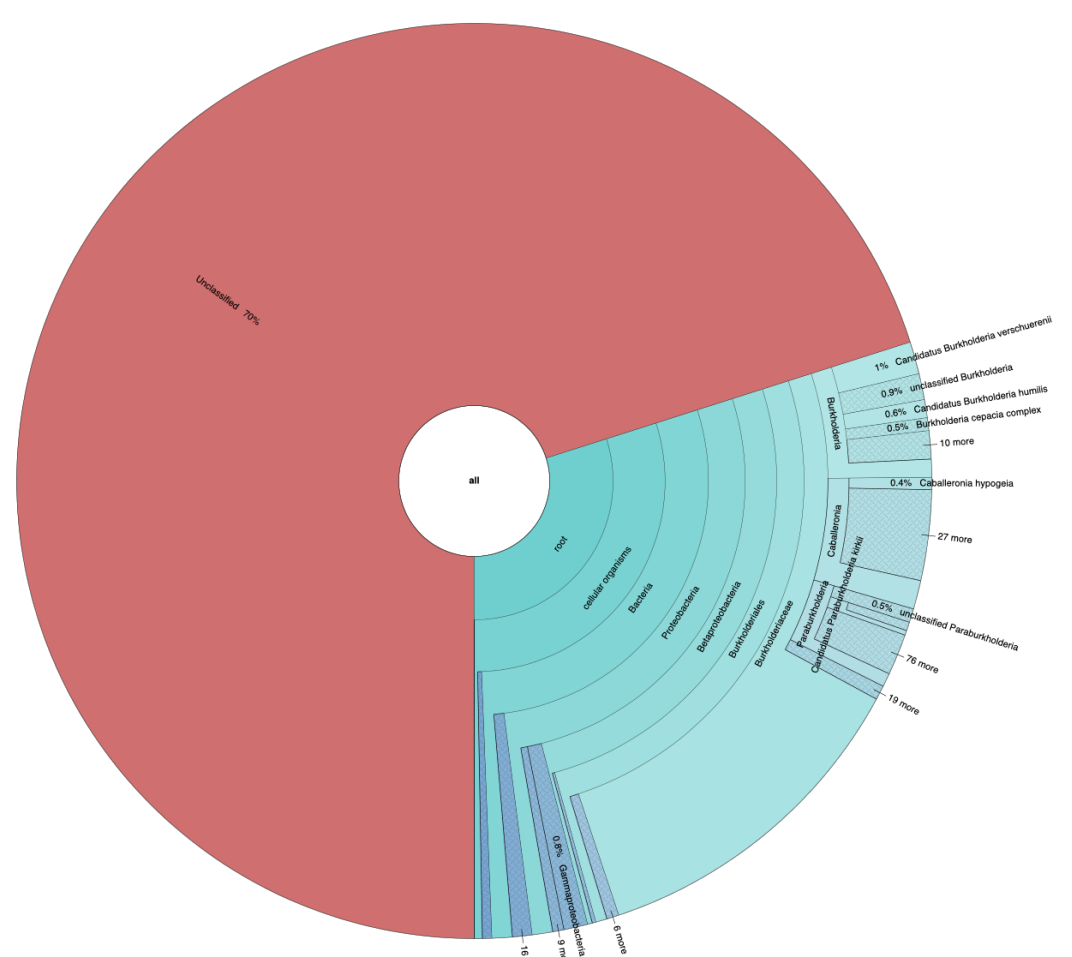

# D

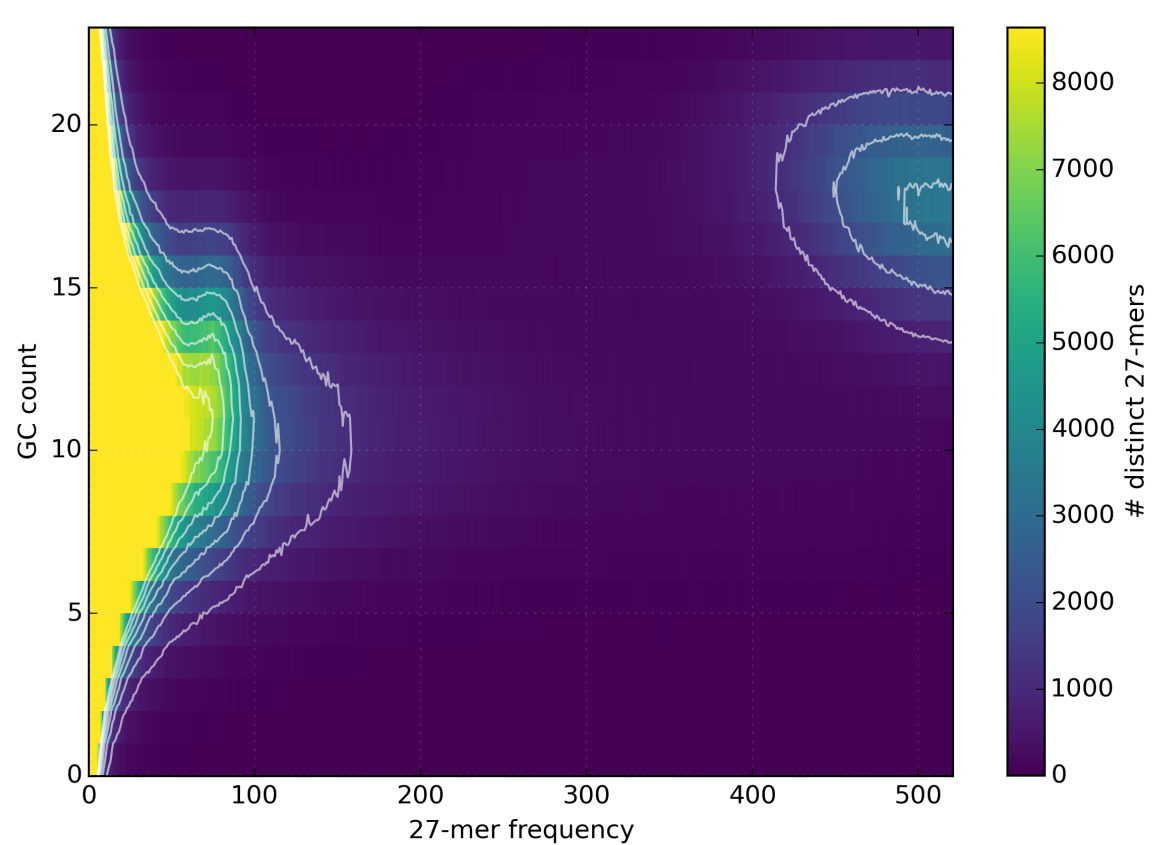

# E

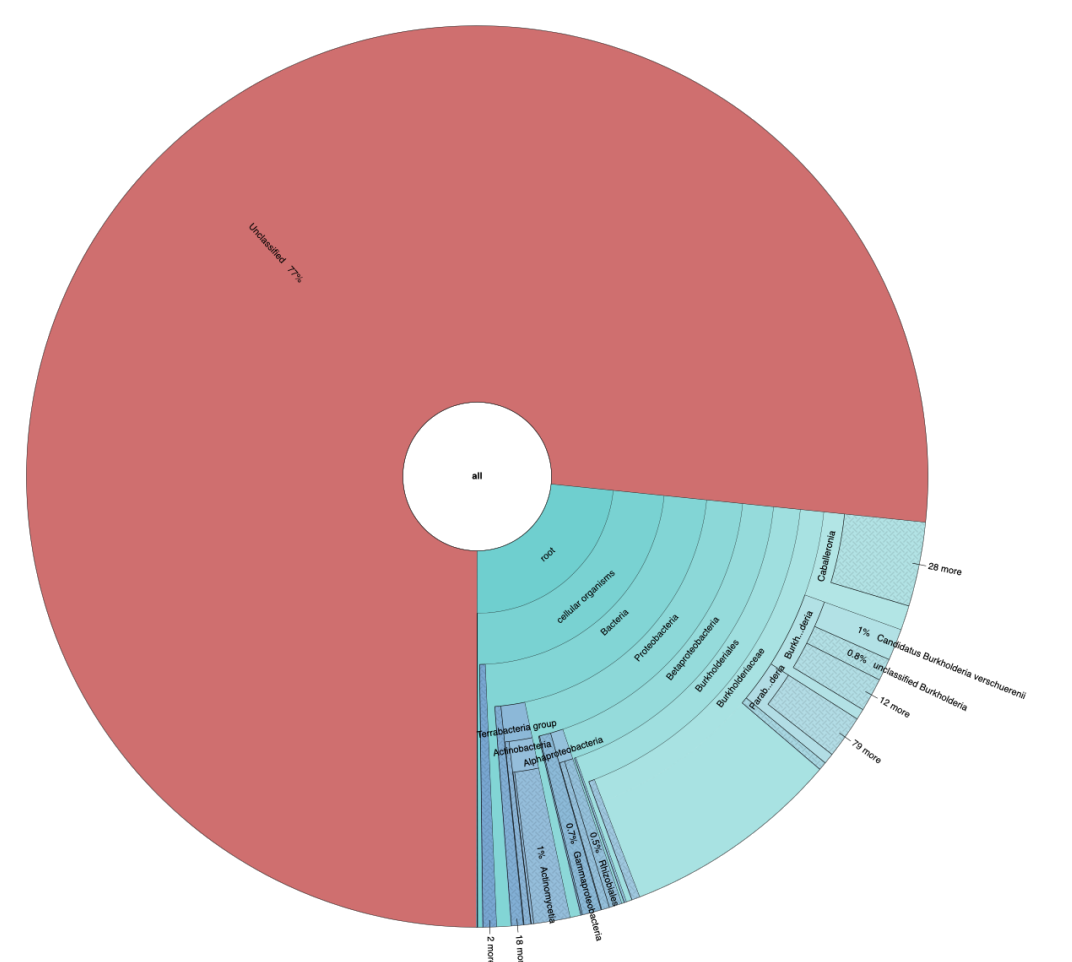**F**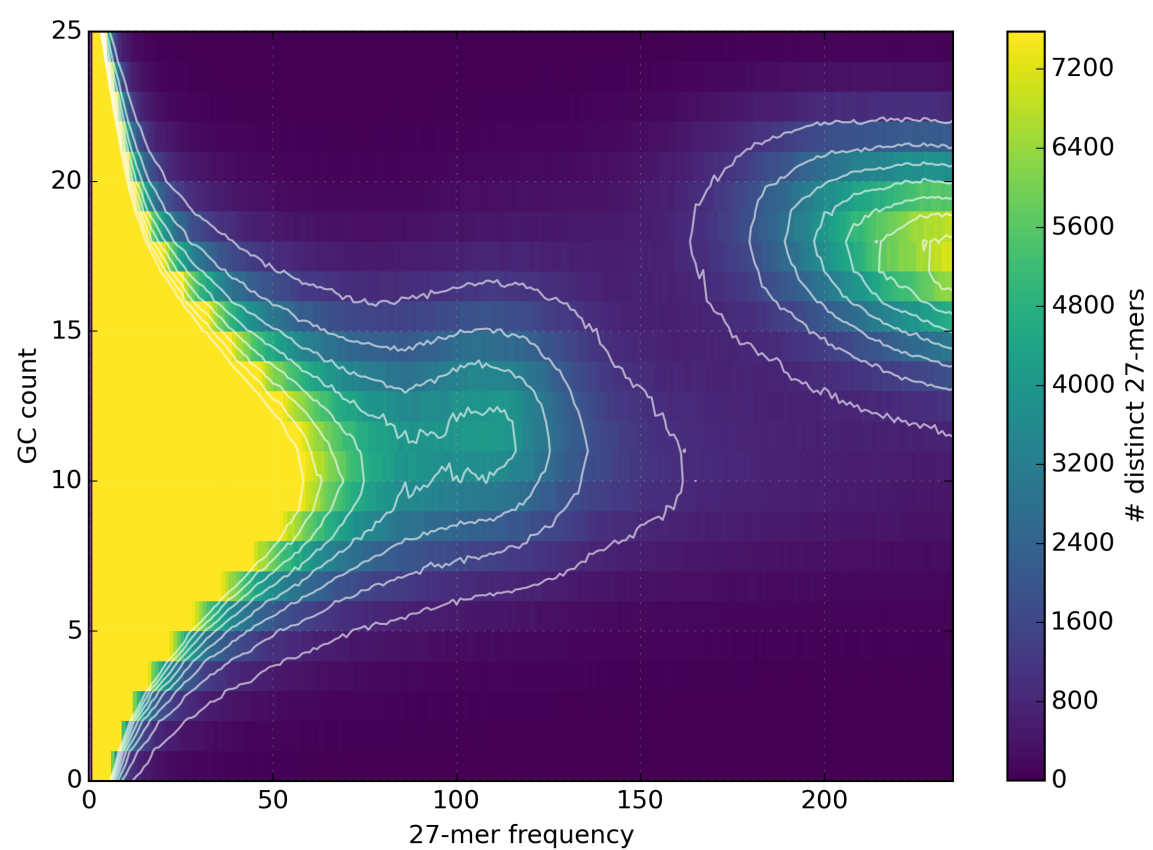

# G

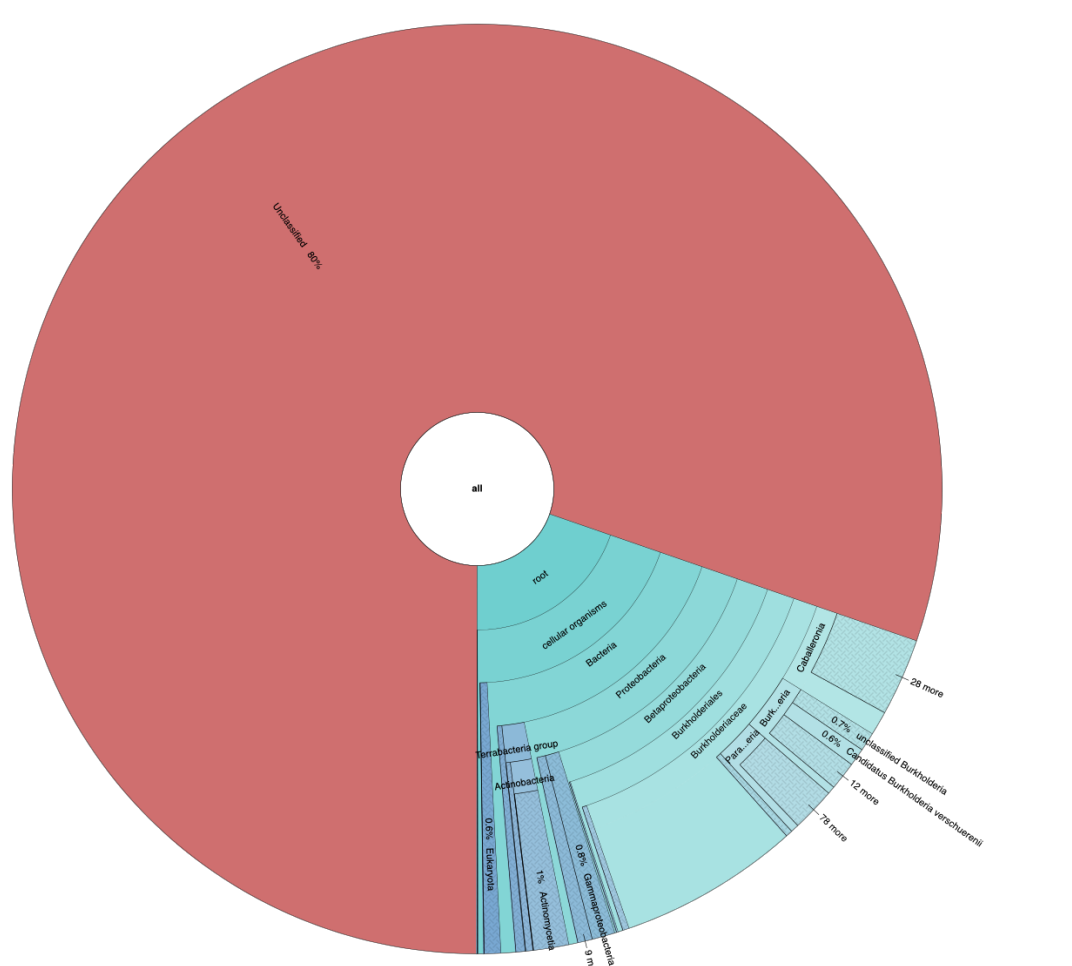

H

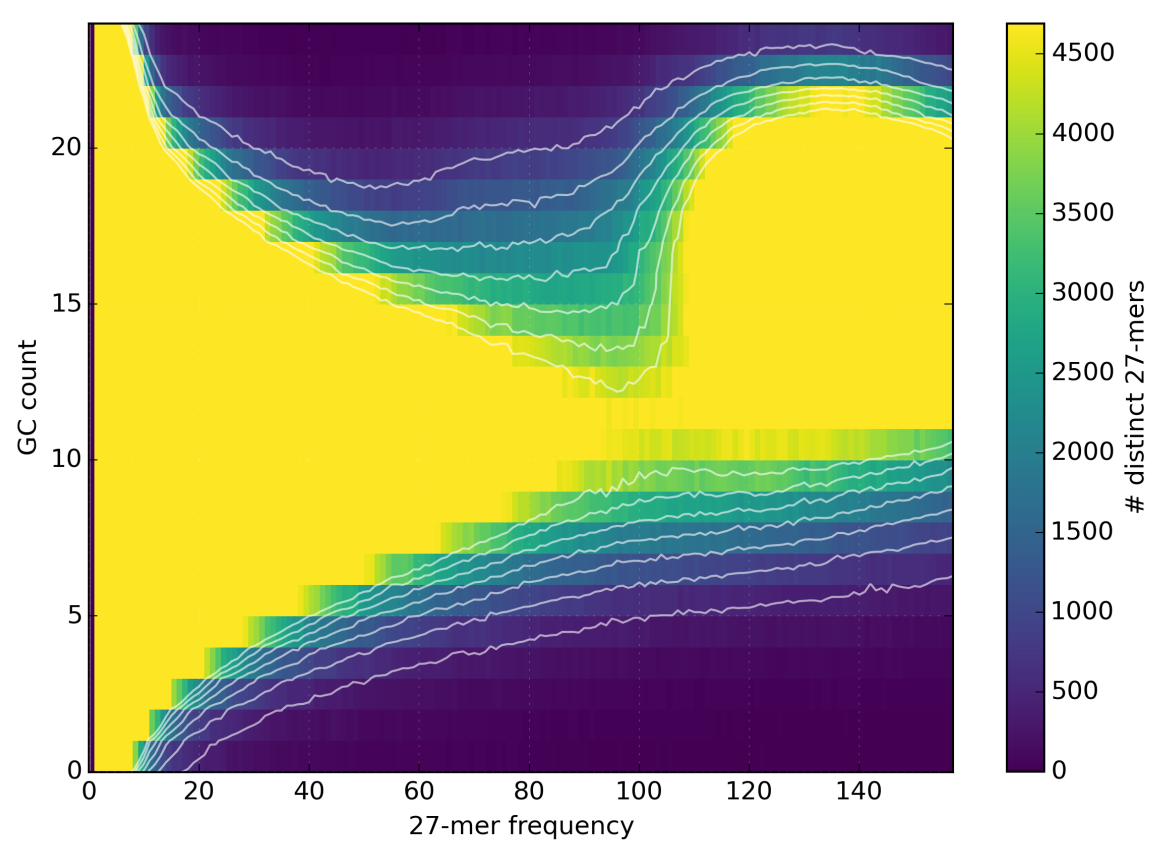

Supplement: Figure S1 — (A) For E. congesta, 37% of all reads is assigned to a taxon name (blue) and about 30% of all reads or 80% of the named reads is assigned to Burkholderiaceae. (B) Density plot of k-mer coverage and GC count per distinct k-mer of the raw reads of E. congesta. (C) For T. semidecidua, 30% of all reads is assigned to a taxon name (blue) and about 25% of all reads or 83% of the named reads is assigned to Burkholderiaceae. (D) Density plot of k-mer coverage and GC count per distinct k-mer of the raw reads of T. semidecidua. (E) For T. lasiodelphys, 23% of all reads is assigned to a taxon name (blue) and about 18% of all reads or 75% of the named reads is assigned to Burkholderiaceae. (F) Density plot of k-mer coverage and GC count per distinct k-mer of the raw reads of T. lasiodelphys. (G) For T. hensii, 20% of all reads is assigned to a taxon name (blue) and about 14% of all reads or 73% of the named reads is assigned to Burkholderiaceae. (H) Density plot of k-mer coverage and GC count per distinct k-mer of the raw reads of T. hensii. [file peerj-11-15778-s001.pdf]
