# Supplementary material for: Metagenomics of African Empogona and Tricalysia (Rubiaceae) reveals the presence of leaf endophytes
Source: PeerJ. 2023 Aug 4;11:e15778. doi: 10.7717/peerj.15778 (PMC10405798; doi:10.7717/peerj.15778)
Supplement: Data S1 [file peerj-11-15778-s003.zip › Empogona congesta.html]

Javascript must be enabled to view this page.

members
magnitude
magnitudeUnassigned

kaiju.out

1999998

749946
101

7405
749586

197
12

1

1

1

3

3

56

47

12

1

1

1

1

1

6

2

2

2

4

4

5

5

1

1

4

4

35

32

13

13

11

11

11

8

7

7

1

1

3

3

3

3

3
1

1

1

1

1

5

5

5

7

1

1

1

1

6

1

1

5

5

5

1

1

1

1

1

117

14

1
14

13

103

103

103

737403
18551

4
48

32

8

5

5

3

1

1

2

2

1

1

1

1

1

23

23

4

4

16

3

13

3

3

4
2

1

1

8

8

2

2

2

2

2

2

2

2

2

2

2

27
164

48

7

8

7

1

22

6

3

35

3517

43

43

43

10

1

1

9

9

9

3

1

1

2

2

2

2

3423

14

14

14

12

12

12

12

12

2

1

1

1

1

5
1

4

2

2

2

2

2

2

78
3404

19
1

4

7

1

5

1

346

346

9

9

9

1

1

1

13

11

11

2

2

289

13

12

1

1

1

1

14

13

1

259

258

258

1

1

1

1

1

1

16

1
15

2

1

1

1

9

5

4

1

1

1

17

1

1

16

16

1

1

266

4
259

22

22

22

2

2

1
2

1

8
1

7

223

1

1

145

20

10

115

49
6

1

1

1

1

1

1

4

2

1

32

1

1

6

6

6

1

1

1

1

1

1

8

8

1

1

1

1

1

1

1

1

1

2

2

2

1

1

1

1

1

2

2

2

7

7

815

815

1
815

718

718

13

9

9

3

1

6
70

4

60

2

2

11
3

3

2

2

3

7

7

7

7

7

1823

1765
155

1303

642

642

661

661

304
5

2

2

2

78

9

69

62

7

219

219

3

2

2

1

1

17
58

1

1

1

1

4

4

4

10

1
10

9

9

19

19

19

2

2

2

1

1

4

3

3

1

1

50

50

50

3

3

7

6

1

1

2

1

1

1

1

1

2

2

1

1

17

3

14

14

1

1

1

16

16

16

1

1

1

1

1

1

35

6

6

6

6

6

2
10

8

19

19

18

1

1

1

17

17

1

1

21

12

1

1

1

1

11

10

10

10

10

1

1

1

9

9

104

96

1

1

1

32

30
1

1

1

26

26

1

1

1

2
1

1

27

27

27

2

1

1

1

18

18

18

3

3

6

1

1

2

2

2

2

2

4

8

8

16788
698756

13

13

13

6723
228

23

23

2

2

2

2

2

1

1

1

1

1

1

1

1

2

2

1

1

1

1

157
21

1

1

2

1

12

1

18

9

2

7

3

16

44

7

3

1

7

1

4

4

4

3

3

1

410

408

6

5
6

1

1

402

402

2

2

2

2

2

6

6

6

6

1

1

1

1

91

2

2

89
2

48
1

4

6

21

21

16

11

11

11

28

3

5
18

4

1

1

2

1

4

7

41

41

14

14

14

9
27

18

18

1

1

1

1

37
1140

38

37

37

1

1

41
622

1

1

9

9

25

25

22

13

12

1

9

10

2

1

7

7

1

1

1

18

18

7

7

13

13

43
5

9

6

3

29

18

18

18

1

1

9

9
3

6

10

10

10

6

4

41

41

22
156

16

94

11

73

3

3

4

3

11

10

4

4

1

1

10

10

3

3

23

23

2
4

2

4
30

26

26

7

4

3

19
105

60

9

9

9

8

1

1

1

363
10

2

2

1

1

1

4

4

5

14
1

13

5

5

2

2

6

6

2
43

3

18

20

23
7

16

8

8

15

15

13

13

12

12

12

8

8

8

1

7

7

19

19

7

7

11
90

18

10

10

1

6

3

1

4

13
26

1

5

5

2

1

1

4

2

2

15

15

41

18

23

8

5

3

7

7

1

1

1

78
1

1
59

58

46

12

18

4

4

14

2

2

2

2

16
487

55
4

29

29

3

24
11

8

4

1

2

1

1

4
3

1

1

1

2
1

1

8

8

2

2

2

3

3

1

1

28
416

1

1

50
4

1

19
44

11

1

12

1

1

1

1

215
33

1

6

1

1

1

1

1

1

1

1

11

1

1

2

1

2

8

1

1

10

1

1

6

46
116

4

1

1

2

1

2

1

2

19

1

1

2

1

3

2

2

1

4

1

2

1

1

1

8

1

1

1

1

2

5

3

3

1

2

3

1

1

1

77
4

5

5

11
15

1

1

2

5

6

32

1

7

2

1

1

1

1

10
34

2

1

20

1

1

1

1

1

117
3588

127
13

2
1

1

1

1

42

26
9

4

13

2

11

1

2

8

8

20

8

12

1

1

10

2

2

39

39

39

59

59
37

16

6

19

3
19

16

20

12

12

1

1

7

7

1
687

44

44

44

33

3

11

16

2

1

4

4

4

9

9

9

17

1

16

7

7

43

27

24

3

16

523
4

498
171

1

3

3

4

2

292

1

4

2

1

4

2

2

1

1

2

2

12

5

3

1

6

6

43
1122

20
36

14

2

122
967

47
496

19

8

20

1

2

2

1

1

6

2

1

255

1

10

6

3

5

65

10

12

10

1

8

4

7

15

1

3

1

1

8

115

8

1

8

10

158

9

29
41

12
4

2

1

5

3
1

1

1

5

5

5

25

2
1

1

23

2

2

4

4

3

1

1

167
2

14
6

8

43

27

16

16

30

15

12

2

1

2
77

5

12

1

1

56

34

8

4

10

1

1

3
653

2

56
1

20
49

1

1

2

26

6

3

2

1

581
41

77

1

29

5

5

27

27

6

6

9

29
1

25
2

23

3

78
419

6

15

13

7

45
103

1

1

16

7

3

4

7

1

2

7

7

1

1

6

143

1

1

45

2

15

15

3

2

1

5

5

3

42

15
11

3

1

12
14

2

13

23

23

23

27

27

27

27

5

5

5

20

1

1

1

1

3

3

1

2

5

5

4

1

10

10

37
11

6

3

2

9

2

2

2

49

3

3

3

1

1

1
6

5

17

11

11

3

3

1
22

4

17

3

1

1

2

2

2

33

4

4

4

19

19

19

1

1

1

7

7
1

6

2

2

11

11
8

1

1

1

1

1

246
7

57
19

5

4

6

23
9

5

9

48
177

12

3

1

1

26
94

1

1

3

3

21

28

1

2

3

5

2

1

7

1

7

5

2

3

3

6

6

1

5

13

13

13

98

18

18

11

7

17

11

6

6

15

15

1

1

24
3

21
5

16

22

10

10

12

1

1

1
537

49
529

6

6

50
3

1

2

12

15
24

2

7

4

2

1

1

1

1

12

12

26

2

2

24

13

13

10

10

4

2

2

1

1

2

2

8

8

1

1

18

18

5

5

5

4

1

3

1

1

2

1

1

13

13

3

10

33

33

17

17

17

4

4

5

5

1

1

1

9

9

9

11

11

4

4

15
7

8

4

4

4

2

2

1

1

1

1

1

20

20
1

6

13

27

11

6

10

8

8

8

8

3

3

1

1

2

1

1

1

5

5

9

9

9

18

18

3

15

6

6

7

7

7

1

1

2

2

2

19

19

19

13

8

8

10

1

3

6

1

5

11

6

5

5

6

6

10

10

2

2

2

1

1

1

1

6

6

1

1

1

29

29

2

2

27

11
27

1

1

1

9

4

654436
4884

126

126

398

1

1

1

366
12

310
5

14

1

270

2

8

1

9

17

27

12

9

3

3

11

2

6

3

8

8

581
43

6

19

4

3

1

11

282

2

12

8

12

1

1

2

3

11

6

1

7

14

12

10

1

13

2

27

4

5

6

7

23

21

1

616665
8223

154
2219

16

16

127

36

15

6
16

10

60

15

15

26
85

15

13

31

15

15

41

41

38

38

3

3

40

8
40

32

106

14

31

31

61

16

5

11

87

16

70

70

1

24
6

6

12

12

103

19

55

29

22

7

9
117

4

15

3

4
86

67

2

6

7

24

17

7

7

113
431

69

8

1

4

8

15

1

2

1

3

17
204

1

3

11

39

1

8

1

123

2

30

23

7

8

8

28
5

9

9

4

2

3

4
5

1

20

20

15
1

13

1

1
9

8

8

36

35

1

1

631
99

19

199

9

3

5

2

32

8

9

28

13

1

32

2

1

13

16
122

5

60

13

16

12

26

6

2

4
143

26

26

20

19

19

1

1

93
1

75

50

25

6

6

11

11

2735
299

29

27

2

2

27
169

60

24

36

1

20

15

22

2

2

34

4

23

7

575
166

5

57
276

1

2

4

7

3

16

84

28

37

17

11

6

3

9

64

23

5

17

4

6

32

13

4

13

2

1

1

20
119

1

8

1

20
77

9

8

2

7

6

6

3

6

9

1

10

2

23

23

23

24
90

16

26

26

24

298
3

106

24

3

14

2

64

26

26

1

11

25

28

29

107
580

7

1

296
86

9

10

60

5

4

4

2

42

6

1

4

8

19

6

1

24

5

3

5

5

2

5

17

5

2

1

2

10

2

14

3

16

25

6

8

19

2

13

4

46

46

201
47

38
56

7

4

7

10

11

1

2

49

17

8

21

6

6

6

9

94

14

15

12

53

119
18

24
65

11

1

1

4

8

7

8

1

24

11

1

4

4

599442
295661

18

18

167

77

31

59

269
1232

18
75

57

19

13

641
1

13

555

6

4

2

16

44

97
109

1

11

1

87
105

18

94

22

72

2620
1132

17

135

41

120
581

29

1

26

98

37

11

6

3

52

15

48

23

35

19

23

4

21

8

2

95
100

5

24

191

2

55

6

4

15

80
83

3

234

14634
127625

8916
957

2119

133

131

148

18

174

617
615

1

1

48

52

137

18

290

756
792

5

23

8

767
966

19

180

981

14
54

25

15

139

168

194

12

56

299
229

70

364
413

20

29

1070

9265

4950

12663

131

122
113

9

3599
495

18

383
306

77

137

25

129

1660
2054

4

10

10

1

5

210

8

21

14

12

16

14

8

61

12

7

26

179
203

24

99

11

39043

9666

1263
17657

3

41

10

3

191

22

84

823

541

585

2

630

32

9

30

287

32

11

20

3

66

18

11

322

510

1

7

10

2

4

26

16

30

32

19

12

8

15

15

2

12

3

313

469

1

57

7

8

46

1

11

3

7

78

21

1

8

8

66

108

15

7

180

2

277

5299

11

129

9

17

26

30

73

160

8

528

50

47

75

20

16

1

54

11

196

350

16

4

134

7

29

189

12

1

3

223

7

25

1722

1

13

152

317

11

32

18

174

5144

765

2070
109

163

124

3

369

320

376

266

340

340

302

302

10
38

2

26
1

16

9

98

9

89

89

1

1

57
318

14

2

5

93
18

18

14

8

2

12

21

2

38

5

35

1

27

30

5

4

33
11

21

1

20

1

10

10

12

6

6

6

609
11

191

407

59360
9257

144

89

684

183

132

234

300

115

166

183

65

237

777
751

26

3851

205

57
35

22

1042

653

330

214

97

158

155
400

245

102

9367
705

10

663

33

25

436

436

155

25

77

72

74

104

83

156

27

568

703

23

60

356

57

31

210

236

68

14

46

485

31

293

188

33

70

28

195

57

217

34

188

140

415

193

90

6

420

7

824

403

342

313

183

294

146

87

232

218

292

594

158

140

153

200

50

99

6675
3449

3226

435

298
234

64

466

870

104

305

92

63

590

65

154

744

197

221

386

405

399

210

406

166

303

328

320

163

670

84

98

942

254

161

147

170

121

9402

105937
23531

2401

681

326
283

43

325

4039

5721

4631

1772

875

446

2134

624

2165

1205

3417

3986

404
6850

34

1047

5365

3308

3145

5114

571

7727

1020

4274

5027

7766

458

2398

64

15

10

10

29

29

409
8

187
318

131

83

2734
832

10

254
1

40

20

8

130

55

19

146

14

211

3

1

47

204

11

125

12

23

13

25

257

12

166

1

58

37

59

14

2

62

23

45

48

30

30

31
802

67

23

42
11

30

1

2

10
25

7

8

8

12

3

9

45

45
15

11

6

13

2

1

1

65
24

13

1

27

12

9

5

1

6
1

1

2

2

17

17

44

44

43

1

44
1

8

5

26

3

1

18

15

2

1

25
2

23

6

11

6

35

22

13

13

4

4

1

1

58

9

4

35

1

5

29

2

4

4

41
2

4

5

6

24

21

1

1

1

25

11
85

12

29

3

14

12

33

13
117

22

4

77

4

9

64

1

35

2

32
10

14

8

1

12
220

13

2

11

5

1

3

5

6

13

4

11

100

20

8

6

186
2881

1

1

641
144

110
353

37

1

3

10

1

1

12

47

12

9

17

17

9

26

6

3

4

1

5

8

3

11

33

89

1

21

45

45

19

23

3

18
89

8

8

1

1

62

19

19

1

1

12

1

11

7

7

7

15

8

7

2

2

11

11

35

29

9

20

6

4

4

1

1

10
176

16

26
112

14

1

27

6

34

1

1

2

38

10

10

206
89

1

116

4

20
191

4

1

20
74

2

18

1

8

3

1

1

5

7

1

7

8

5

15

30

32

2

17
31

3

6

5

5

2
22

1

1

19

2

20

20

27
131

15

3

5

2

4

70
11

2

9

3

45

5

53

20

32

5

3

3

21

1

5
289

234

6

44

17

6

21

101

29

3
47

3

5

9

1

26

11

2

11

1

31

31

3

3

12
101

4

8
61

8

12

10

21

2

21

3

134

134

120
1

1

118
49

31

8

4

16

4

6

47
1

17

9
20

1

9

1

9

48

48
1

12

9

14

9

3

4

14

5

9

9

63

20

8

5

4

26

3

6

1

7

1

1

3

3

1

11

11

21
767

68
5

26
1

25
8

11

5

1

12

3

8

8

1

6

6

6

17
11

6

2

2

108
19

2

2

9

9

21
4

17

17

4

4

14

1

13

23
10

2
9

7

4

16
6

2

8

51

5

5

28

18

18

10

6

6

5

4

1

7

7

279
6

36
23

2

10
6

4

1

8

1

1

7

26
229

7

10

27

2

1

1

30
155

10

1

61

11

37

5

20

20

18

2

60
29

5

1

12

1

12

108

9

9

6

6

5
93

66

66

10

12

52

1

1

43

38
1

37

5

8

8

10
30383

322
9

25

14

11

34

20

10

4

5
30

12
1

11

13
5

8

28
151

21

21

1

26

8

5

13

35

35

4

1

8

8

7
4

3

3

22

22

5
2

1

1

2

1

1

19

19

11

11

69
30051

1

1

1
37

7

15

15

9

5

92
10

76

71

5

6

15

35

33

2

45

21

24

4

4

4

59
7

21

25

25

6

19

17

2

3
29

1

22

3

9
10

1

3

3

3

29558

29540
13

56

14

27314

2116

15

15

12

9
18

7

5

2

2

8
1

6

1

2
32

30

14

16

8

8

27

7

7

7

39
625

3

3

3

277

7
1

6

6

10
5

1

4

4

20

20

14

14

102

4

98

31
58

9

12

6

25

3

14

8

1

1

1
23

11

11

17

8

8

9

1
202

2
33

1

4

4

10

12

3

1

8

26

21

3
5

2

17
121

11

9

23

8
40

22

1

9

10

10

1

21

6

3

3

15

7
98

26
86

1

59

3

11

14

24

5

2

5
1

3

1

6

1

5

824
19504

81

2

1

1

1

79

55
14

9

2

23

4

3

7

7

16

16

1

1

1

40
3207

41
1357

36

13

13

2

21

806

732

17

35
11

8

14

2

18

4

23

23

4

4

16
125

29
106

13

10

5

2

32

4

11

1

2

6

5

1

1

36
271

13

59

6

22

10

5

77

20

2

9

1

14

1

5

1

14

10

43

8

8

9

9

7
23

16

16

3

3

2

2

14

14

18
1796

4
19

15

5
180

175

219
3

63

4

1

1
57

6

2

47

1

3

8

3

18

3

56

679
1096

29

14

15

15

16

1

17

7

34
33

1

1

1

1

1

4

4

9

14

3

262

166
20

17

11

6

6

2

119

119

2

62
2

10

1

1

2

10

35
15

13

7

1

11
1

2

8

6

2

10

1

1

5

4

15

1

14

356

3

3

3

3

15

15

14
4

10

10

8

2

23

9

14

14

137

4
14

5

5

6

6

3

3

5

3

1

1

7

7

2

1

1

7
50

1

19

5
23

3

14

1

6

6

4

4

9

7

2

2

31
29

2

2

164
13

1

1

16

8

8

1

1

1

29

1

28

2

14

14

12

10

1

1

6

6

16
33

2

5

10

3

4

3

14

14

22

22

125
1

86
8

10

1

9

3

6

4

4

1

1

2

2

61

61

38

38

4

12

8

2

1

4

7

7

7
1

6

6

12

12

6

6

6
1

4

1

104

5
104

33

3

30
13

17

13

4

5

5

32
19

12

1

2

2

15

15

1

1

1

7

6

1

4

27

27

11

11

12

12

4

13
5133

4169
32

125

109
1

58

13

37

16

16

4

4

10

10

3997
1110

443

125

1

6

219

92

9

808
205

4

1

18

12

15

9

4

6

3

3

9

4

10

2

1

29

38

3

7

1

6

9

4

16

2

12

5

14

7

30

13

1

3

16

28

5

4

11

28

5

1

2

2

9

1

12

5

1

9

10

1

6

1

10

2

8

67

4

8

4

26

3

10

3

54

5

2

8

1

71

1

21

18

13

18

15

1

144

9

4

1

2

3

7

10

6

1

12

7

17

8

1

4

11

21

35

26

1

6

145

145
58

87

3

1

7

21

10

479

450

2

18

1

4

4

4

326
16

5

2

3

92

72
92

20

18

2

119

3

15

1
76

7

54

14
12

2

1

1

1

32

22

22

10

10

1

7

22

65

2

1

1

951
3

12

4

8

1

1

855
69

15

1

1

2

2

10
23

1

2

1

7

2

2

606
18

554

1

22

11

126

1

1

6

13
9

4

1

1

1

1

50
2

1

3

6

28
38

1

1

2

6

15

9

6

682

33

25
11

1

13

8

8

486
6

6

5

1

9

9

1
103

102

1

1

5
23

11

7

1

1

323
35

1

7

7

1

3

4

5

29

3

13

10

4

156

1

4

12

35
5

3

1

1

1

1

13

2

4

4

3
14

4

7

30

12

12

3

2

2

1

15

14

1

1

1

1

1

1

1

1

1

1

1

129

1

1

9

9

2

2

42

4

1

8

3

5

11

5

9

2

2

1

1

46
13

15

1

4

4

13

8

8

8

6

6

14

14

1

1

1

204

140

10
140

120

117

3

7

3

15
64

19

17

2

4

4

2

2

20

20

4

1

1

3

1
112

2

1

1

103
5

13

13

31
1

1

3
29

9

17

17

17

4

4

9
8

1

3

3

10

9

1

8

1

1

8
11

2

1

1

1

6

1

1

5

5

1397

1397

11

11

27
535

45

81

4

3

52

20

2

3

6

1

13

34

101
17

1

83

83

83

38

1

1

12

172

45

6

121

833

5

5

10

10

796

22

1

12
5

7

5

2

4

4

1

140

58

3

3

13

13

24

20

4

1

1

15
2

13

13

2

2

65

6

6

4

4

19

19

1

1

1

12

12

4

4

4

6

6

13

8

5

6

6
4

1

1

11

5
11

4

2

2

244
8

3

7

7

2

1

7

1

5

5

1

10

6

8

4

1

2

2

164

104

8

8

8

53
1

4

4

9

9

1

1

11

11

4

3

4

22

6

16

1

15

1
2

1

3

3

6
39

11

11

11

8

8

6

2

4

8

2

2

6

2

2

2

1

1

1

1

9
493

12

12
2

5

5

1

1

3

170

1

1

1

5
7

1

1

1

7

3

3

6

6

4

4

36
89

2

5

4

1

5

4

6

6

1

11

3

10

44

44

8

5

3

1

1

1

5
44

4

4

4

27

2
8

2

4

15

1

5
14

2

1

1

7

12
1

1

1

1

10

1

3

2

1

6

217

1
207

196
7

5

119

6

3

2

54

10

10

10

14

5

5

9

9

9

6

6

6

6

5

5

3

2

2

108
6088

34
495

332
10

12

2

268

1

13
39

3

18

5

13

13

9

9

9

90
4

6

9

28
37

9

1

11

22

17

6

11

11

11
4

7
5

2

17

17

17

4474
165

22

1

2

19

4
1240

1216
1100

116

2

1

5

2

10

822
8

181

79

31

1

5

4

30

3

23

1

4

578
633

55

8

11

1

35

1

1

9
17

6

6

2
1

1

561
22

18
406

66

28

79

60

155
3

36

2

1

1

7

19

86

4

129
2

1

14

16

1

7

12

5

71

4

4

15

1
17

16

4
1464

2

1447
1441

1

5

11

2

1

8

17
2

3

2

10

10

40

1

39

2

2

22

20

2

9

9

3

3

6

6

1

5

5

38

3

1

34

15

19

14

2

2

2

1

1

28
254

4
44

39

1

1

1

1

52
111

35
14

7

3

11

11

1

1

11

11

2

2

16

16

4
52

3

11

6

7

3

10

2

6

120
20

39

38
7

29

2

1

41

30

10

1

20
6

12

1

1

28

5

5

23
8

7
5

2

8

581

42
1

4

37

124
238

6

13
17

4

73

18

5

5

43

43

3
152

149

10

10

10

91
26

57

8

153

10

1

9

9

16

16

1
53

8

8

1

43

3

3

31

18

1

9

9

3

1

1

13

5

5

4

4

4

4

3

3

22

22
1

6

10

2

3

1

1

12

12

12

12

5

5

7

31

69

6
69

23
24

1

39

12
39

6

21

21

1011

14
990

6

6

6

6

144

1

1

140

61

61

73

5

68

68

6

6

3

3

2

1

7

7

6

1

1

227

3

1

2

2

2

2

2

2

160

34

22
34

12

10

2

81

2

2
61

13

13

4

42

4

4

14

14

45

10

10

1

1

28

28

6

6

62
2

11

11

11

36

1

1

10

10

25

7

18

18

12

12

12

1

1

1

8

8

8

538

15

15

14

1

523

16
520

4

4

488

488

2

3

1

2

2

2

1

1

1

31

10

2

1

1

1

16

15

1

1

4

4

1

3

9

9

1

1

8

1

1

1

1

21

21
1

4

4

1

1

3

3

14

9

1

1

1

8

8

5

5

5

5

2

1
2

1

1
2

1

138

24

6

7

5

95

1

205

24

2

179

581
15579

1

1

1

1

1

1

24
1

23

2

2

2

1

1

21

21

21
1

1

12

6

1

8

1
7

6

1

1

1

1

1

97

97

10

10

10

10

10

7

7

7

7

7

80

80

80

80

67
10608

15

15

15

15

15

20

20

8

8

8

8

12

12

12

10416
715

256

4

4

252
3

2

1

1

1

1

46

1

1

28

16

4
7

3

1
21

1

10

2

7

6

1

3
8

1

1

1

1

1

1

1

1

1

1

14

31

1

1

10

9

1

106
2

33

33

1

17

2
14

12

15

1

17

5

1

1

1

1

1

1

1

1

14
1148

1
680

1

56
166

4

17
84

1

2

9

27

12

1

15

7

11

2

2

1

1

512
164

1

3

2

1

3

1

1

1

1

1

16

4

7

1

3

40

140

117

8

36

36
3

7

7

1

18

7

5
344

16

16
5

11

44

6

3

23

1

5

5

3

1

1

1

39

39

39

9
240

1

1

2

6

6

184
26

4

53

3

1

81

4

11

1

2

2

17

11

6

1

1

17

64

4
64

43

17

10

5
10

5

23

20

3

320

5
320

6

6

20

5

15

1

1

1

1

2

1

1

1

1

22
7

4

4

1

9

1

1

1

260

1

1

1

258

21

4

4

2

2

17

17

17

97

97

4

4

2

2

1

1

84

84

84

6
5

1

1

1

1

1

1

524
18

127
3

1

1

4

4

23
20

3

3

39

12

12

27

1

1

56

52

4

58

3

3

13
55

42

321

4
318

1

33

16

1

13

2

264

3

3

4083

4083
44

8

7

1

3946
796

5

14

67

7

10

7

2

45

45

3

82

1

8

1

104

3

1

29

29

29

2

22

210

8

6

1

190

93

47

118

204

12

127

10

15

32

12

2

1

1

3

1

1

9

145
788

1

1

17

1

41

1

6

37

1

32

1

8

53

100

12

4

23

64

1

1

3

1

7

21

5

181

5

15

508

34

2

3

120

62

5

16

12

84

65

2

1

1

61

1

1

20

20

1

1

1

1

11

11

3

3

8

8

1565

2

1

1

1563

1563
245

55

54

1

269

655

339

8

8

4

4

4

3

1

5

5

1

1

4

4

4

54

9
54

9

7

1

1

28

1

1

27

1

1

7

5

5

2

1461
36

37

37

37

5
1134

2

2

2

3

3

964

964

9

2

1

1

7

1

1

150

148
9

2

1

1

11

1

47

2

1

1

71

1

1

1

11
1

10

10

5

5
1

4

3

3

3

7
198

5

4

4

1

3

1
3

2

10

10

2

1

1

1

1

99
7

2

2

88
1

1

6

2

8

67

1

2

1

1

1

1

16

16

46

42

3

1

1

4
1

3

3

1
3

1

1

2

2

2

8

8

5

4

2

2

1

1

4

2

2

1

1

1

1

17

1
4

1

1

1

1

1

1

9

2

2

5

5

2
1

1

1

1

1

1

1

114

21

21

5

8

1
8

6

1

16

16

16

18
4

1

1

1

6

6

1

1

6

5
6

1

59

4
41

1

3

1

1

1

3
21

16

1

1

1

8

2

2

1
6

4
2

1

1

1

1
2

1

2
6

1

1

2

2

2

8

8

8

6

1

1

2

2

2

70

70

3

3

2

1

11

2

1

8

56

2

2

54

52

52

2

2

1

1

1

1

1

1

1

1

16

6

10

233
2

14

2

2

2

12

12

13

13

13

4

4

4

2

1

1

1

1

1

1

1

35

35

32

22

22

22

22

10

4

4

6

6

1

1

5

129

1

1

7

11

109

2

2

2

2

2

2

2

746

45
733

4

4

4

4

4

21
329

1

1

1

25

5

5

1

1

19

19

19

32

32

32

6

6

6

5

5

55

5
4

1

50

50

43

6

1

27

1

1

26

26

26

1
157

155

3

141

3

3

8

1

1

3

2

1

1

1

185

42

32

32
1

31

1

9

6

6

1

1

1

1

1

1

1

1

143
18

39

4

4

5

5

5

3

3

2

1

27
1

26

56

1

1

39

5

5

34

13

13

13

3

3

1

2

1

1

1

28

8

8

8

1

1

19

19

19

1

1

144
17

72

5

5

67

20

47

42

1

4

1

1

1

1

1

1

4

4

4

4

14

14

13

1

12

1

6

6

1

1

5

9

9

9

20

7

7

13

1

1

1

11
1

2

1

1

5

1

7

5

2

19

19

19

19
1

18

2
3

1

10

2

2

2

8

1

7

3281
24

44

44

1

1

1

43

3

3

34

34

6

6

367

1

1

1

1

1

1

1

154

154

154

154

20
211

55

55

1

54

11

9

9

2

2

3

3

19
35

1

1

4
6

2

2

3

3

6

6

6

5

5

5

5

6

3

3

1

1

2

2

13

12

4

5

5

1

2

1

1

1

1

60

50

33

17

1

8

8

8

1

1

1

1

2

1

1

1

1

30

30

4

4

4

4

26

17

17

9

9

69

25

24
4

1

1

1

1

16

16

3

3

1

1

1

1

29

29

4

4

5
25

20

20

15

15

15

15

6
2743

526

2

2
1

1

4

3

3

1

44

44

3

3

1

2

20

9

3

3

3

3

3

1

1

2

291

9

9

282
1

9

13

11

1

1

253

6

138
2

25
14

11

12

12

4

4

1
3

2

20
16

3

3

1

72

43

29

47

47

47

7
2211

9

1

1

1

1

7

7

1
882

129

129

725
1

15

11

687

10

1

27

26

1

1

1024
18

3
6

3

3

1

1

4

4

4

1

1

14
804

2

1
110

65

27

38

44

265

214

23

8

20

3

2

1

3

119

1

281
3

1

1

73

4

13

1

82

103

3

1

1

4

8
16

8

1

1

3

3

12

12

133

132

1

20

20

20

85

85

85

41

5

1

1

1

1

1

34

8

26

124

22

22

5

17

1

1

101
2

2

10

4

4

13

57

28

7

1

4

1

2

1

2

4

7

9

30

2

2

9

9

1

18

18

18

1
6

1

1

1

1

1

1

2

2

3

2

2

2

2

1

1

1

4

1

1

1

1

19

1
19

5

1

5

7

1
46

11
13

2

32

30

30

30
11

19

10

9

2

2

8

8

8

8

2

2

6

6

2

1

1

1

1

1

1

141

19

19

19

1

1

1

1

1

39

39
1

38

2

1

1

1

12

11

10

1

1

14

14

1

1

4

5

1

1

3

10
82

1

54

17

1

1

1

1

237

5
103

2
30

1

1

1

19

6

3

2

2

1

1

1

63
1

16

16

11

11

5

5

33

14

14

14

5
19

7

7

3

2

1

1

1

3

3

3

12

12

8

8

4

4

1

1

1

2

2

2

1

1

1

6

6

3
92

1

1

1

5

5

5

5

5

30

30

2

2

2

14

2

2

1

1

1

1

5
2

3

5

5

9

9

9

5

5

44

3

1

40

9

1

1

1

1

8

8

7

7

1

1

1

2

2

2

9

9
4

1

1

3

25
1

3

21

14

14
5

9

9
4

5

7

4

4

4

3

1

1
2

1

4581
356

21

20

1

1

1

1

19

19

19

19

1

1

1

1

2275
1

2271
74

16
918

536

532
1

1
500

4

4

1
4

2

2

2

1

1

494
5

65

2

2
1

1

63

3

1

1

1

1

1

1

1

1

1

1

1

1

60

12

12

5
12

7

45

2

2

5
30

16

6

1

1

1

1

5

5

5

4
8

2

2

1

1

1

1

3

1

1

1

2

2

1

1

90

90
5

31

1

1

1

1

1

1

1

1

1

1

1

1

1

28
1

1

1

1

1

2

2

2

1

1

1

1

1

1

1
16

1

1

1

1

9

1

3

2

2

1

1

1
4

3

1

1

1

1

1

1

1

1

1

4

2

1

1

1

1

1

1

1

1

1

1

3

3

1

1

2

2

2

1

1

1

1

1

1

15

1

1

1

1

1

10

10

10

10

10

1

1

1

1

1

1

1

1

1

1

1

1

1

39

1

1

1

1

1

13

13

13
4

6

6

3

3

3

3

3

19

4

1

1

1

2

2

2

15
1

2

2

1

1

5

5

5

3

2

1

3

3

3
1

2

3

3

3

3

6

6

1

1

1

1

1

5

5

5

5

5

328
1

193

162

162
1

1

1

160

160

18

1

1

1

1

1

3
5

1

1

1

1

11

11

11

1

1

1

13

13

13

13

13

134

19

19

1

1

18

18

18

1

16

1

56
1

49
1

4

2

2

2

2

1

1

40

40
5

1

1

3
1

1

1

2

2

2

1

1

1
5

4

1

1

15
3

11

1

5

1

4

2

2

2

1

1

1

1

1

2

1

1

1

1

6

4

1
4

1

1

1
2

1

2

1

1

1

1

59

1

1

1

1

1

1

1

1

3

2

2

2

2

1

1

1

54

54

54

51

3

1

1

1

1

1

1

31

31

8
31

1

1

1

1

1

1

1

1

5

1

1

1

1

1

1

1

1

1

1

1

1

1

1

9
1

6
2

4

1

1

3

2

2

2

2

2

2

2

2

2

2

2

2

4

2

2

2

2

1

1

1

1

2

2

2

2

366
2

91

1

1

1

1

1

1

86

86
38

46

38
45

1

3

3

3

3

3

1

1

2

2

2

2

4

2

2

2

2

2

2

2

6

6

6

6

6
1

1

1

3

3

25

1

1

1

1

1

1

24

11

11

11

11

9

7

7

7

2

2

2

1

1

1

1

1

3

3

2
3

1

242

3
109

1

1

1

1

98
4

8

8

8

8

8

8

6

6

6

59

3
26

6

2

2

2

2

10
8

1

1

3

3

33
5

22

22

3

3

3
1

1

1

13

9
13

1

3

3

7

7

2

2

2

2

1

1

2

1

1

1

1

28

28

28

26

25

25

1

1

2

2

5
105

56

1
18

1

1

1

5

1

1

1

1

2
1

1

1

1

1

2
1

1

1

9

1

1

8

8

8

33

1

1

1

1

6

6

6

6

26

1

1

25

7
25

18

18

1

1

1

1

4

4

4

3

3

1

1

1
44

1

1

1

1

1

5

1

1

1

1

1

1

1

1

1

1

1

1

1

1

1

2

2

1

1

1

1

1

1

37

6

1

1

5

5

2

2

1

1

3

1

1

1

2

2

2

1

3

1

1

2

1

1

1

2

2

1

1

1

1

1

7

7

7

7

4

4

4

4

1

1

3

3

4

2

2

2

2
1

1

1279
10

3

3

3

3

1

1

1

1

2

2

2

2

4

4

4

4

4

13

7

7

7

6

1

4

2

2

2

2

2

1

1

1

1

1

12

2

2

2

2

2

2

2

7

1

1

1

1

1

1

6

6

6

5

4

1

1

1

3

3

3

3

3

3

1198
1

187

187

187

187
49

17
67

41

6

2

1

3
1

2

63

1

62

2
5

3

1

1

1

609

3

3

1
3

2

2

606

2
606

1

1

1

1

15

15

4

11

2

2

2

48

46
48

2

2

538

1
324

2
316

1

313

5

308

7

1

2

4

214

214
36

13
9

4

12

153

401

401
2

364

23
364

50

50

291
9

92

148
66

32

50

42

42

35

35

35

35

39

39

3

3

3

3

3

3

19

7

4

4

4

3

3
1

1

1

1

1

1

1

1

1

3

3

3

2

2

2

2

3

3

3

3

3

3

1

1

2

2

17

17

17
13

3

1

2

1

3

3

3

2

2

1

1

1

96
14

2

1

1

1

1

1

1

1

1

31

10

10
2

1

1

1

1

1

5

5

1

1

14

14

14

14

7

7

7

1

1

6

5
49

11
42

24
5

18

6

6

6

12

12

5

5

7

1

1

1

1

1

1

1

6

6

6

6

1

1

1

1

1

1

1

1

1

13

9

9

9

9

4

4

4

4

4

36
1

11

11

11

11

11

11
2

9

24

1

1

1

1

16
1

15
1

5

2

2

2

2

3

3

3

1
9

2

2

2

6

2

2

4

4

7

7

7

7

7

35

5

5

5

5

5
1

1

3

2

2

1

1

1

1

1

1

28

13

13

11

11

6

6

6

1

1

1

4

3

2

1

1

1

1

1

1

2

2

2

2

15

15

15

8

8

8

7
4

3

3

3

356

75
1

6

2

2

2

2

4

4

4

4

5

5

5

5

3
5

1

1

5

5

5

5

5

1
25

10

9

9
1

8

1

1

7

7

7

1

1

1

14

11

11

1
11

1
3

2

2

4

3

3

1

1

3

3

3

3

3

3

3

3

3

33

31

10

3

1

1

1

1

1

2

2

2

5

5

5

5

2

2

2

2

2

21

21

21

18

18

18

18

3

3

3

2

2

2

2

2

2

260
2

1
40

17

17

8

8

8
3

5

5

9

9

9

9

7

7

2
7

1

1

1

4

4

1
15

10

5

5

5

5

2

3

3

5

5

5

5

5

5

3

3

2

2

1

1

1

1

1

1

1

1

1

1

1

1

1

1

1

211
3

8

8

6

6

2

1

1

24

3
24

2
1

1

1

2

2

2
17

4

3

1

7

1

1

1

1

175

175

149

149

26
8

1

5

3

4

3

2

2

5

3

2

2

2

2
1

1

1

1

1

1

2

2

2

2

2

2

2

2

2

21

7

7

7

7

7

7

5

5

5

5

5

5

9

7
8

1

1

1

1

1

1

1

1

1

1

1

1

10

10

10

5

5

5

5

5

5

5

5

4

1

1

14

1

1

1

1

1

2

2

2

2

2

2

11

11

11

11

11

1368

131
1368

13
654

269
9

25

25

25

25

235
6

10
68

10

10

10

7

7

7

20

20

3
20

9

8

21
3

4

4

14

14

161
13

1

1

1

1

1

1

4

4

4

4

4

4

7

7

7

4

4

4

17

7

7

10

10

10

110

106
1

74

20

11

11

1

1

3

3

28
372

43

33

6

6
4

2

27

27
1

26

10

5

5

5

5

14

14

14

14

2

2

2

2

228

228

1

1

8

8

8

3

3

6

6

45

45

165
4

5

5

1
156

1

1

118

118

19

16

56

56

18

7

11

38

38

38

1

1

1

1

35

35

35

35

35

25

25

25

25

25

25

13

4

4

4

4

4

9

6
9

3

3

116

116
9

73

73
8

42

42

23

2
34

20

20

12
4

3

3

3

5

44

44

44

44

44

44

50

50

50

50

29

21

30

30

30

30

37

37

37

6

4

2

31

30

1

5
232

10

10

10

10

10

1

1

9
135

123
2

110

110
66

5

9

1

20

9

11

11

11

3

3

3

3

81
6

6

6

6

6

6

69

59

2

2

2

1

1

1

56

56

37

19

19

1

1

2

2

1

1

1

1

7

7

7

259

1

1

1

1

1

1

1

1

258

5

5

12

12

12

12

12

12

12

2

10

241

241

241

241

14

13
14

1

227
6

137

137

3

1

2

1

1

1

1

75

75

5

2

1

1

3

1250052
