# Supplementary material for: Metagenomics of African Empogona and Tricalysia (Rubiaceae) reveals the presence of leaf endophytes
Source: PeerJ. 2023 Aug 4;11:e15778. doi: 10.7717/peerj.15778 (PMC10405798; doi:10.7717/peerj.15778)
Supplement: Data S1 [file peerj-11-15778-s003.zip › Tricalysia hensii.html]

Javascript must be enabled to view this page.

members
magnitude
magnitudeUnassigned

kaiju.out

2000000

394104
41

1
562

3

1

1

1

2

1

1

478

475

475

475

1
475

32

1

1

1

1

31
12

10

9

3
442

273

273

124

124

11

1

1

9

7

2

7

7

6

6

1

1

1

17

5

12

11

1

3

3

3

3

3

3

3

3

24

24

24

24

24

6

3
2

1

3

2

2

1

1

18

16
11

1

1

1

1

1

1

1

1

1

1

1

53

53

53

53

53

27

2

2

2

7
2

1
5

3

1

5

5

5

7

4

3

1

1

1

2

2

2

2

2

2

1

6

5
6

1

9

1

1

1

3

3

3

4
1

1

1

1

1

1

1

3

3

6
8

2

2

2

1

1

1

3968
393501

1
260

28

5

5

5

5

1
4

3

2

1

1

1

21

13

2

1

1

1

1

1

1

1

11

1

1

4

6

6
2

4

8

3

3

1

1

2
1

1

1
5

2

1

1

1

1

1

2

2

2

2

2

1

1

1

1

1

1

1

1

1

1

1

1

1

9

9

219

2

2

2

2

1

1

1

1

19

19

19

198

198

198

518
11445

3
1544

303

11
1

1

1

1

1

1

1

1

1

8

8

8

8

4

4

4

4

4

11

11

11

11

11
1

1

1

3

6

1
147

145

9

2

2

2

2

2

2

2

2

2

5

4

4

4

4

4

1

1

1

136

136

136

2

2

2

134

134

134

134

1

1

1

1

1

1

130

105
1

98

1

1

1

97
1

95
2

90

90

89

1

1
3

1

1

1

1

1

1

1

1

1

1

1

5

5

5

25

16

16

16

1

1

1
11

1

1

4

4

5

5

4

4

1

2

1

9

9

9

9

9

25

5

5

5

5

5

5

14

14

14

14

14

14

5

1

1

1

1

1

3
4

1

1

1

1

1

1

1

1

3
1213

18

10

4

4

1
4

2

2

2

1

6

6

6

6

8

8

8

8

8

9
844

14

1
14

5

5

8

3

3

2

769

2
769

1

1

39
5

2

6

9

9

7

5

1

1
2

1

2

722

721

1

2

2

3

3

3

8

8

8
3

5

5

44

3
44

33
4

7

3

3

16

5

5

1

1

2
1

1

1

5

5

2

2

3

3

343
14

22

1
22

19

19

19
11

2

2

6

2

1

1

1

1

1

1

26
270

22
230

22

9

9

9
13

2

1

1

2

179
99

12

12

12

39

39

39

29
8

5

3

12

1

7

7

7

1
14

11

1

1

4
33

1

1

1

1

1

1

12

12

2

2

2

2

1
9

6

6

6

2

2

2

2

1

1

1

1

1

15

4

4

4

4

4

4

10

10

10

10
2

2

6

6

1

1

1

1

1

1

1

1

1

1

1

2

2

1

1

1

1

1

1

1

1

1

1

1

1

1

1

1

1

95
5

1

1

1

1

1

1

1

1

1

1

62

1

1

1

3

3

3

3

3

3

58
2

5

5

5

5

1

1

1

1

1

1

1

1

3
44

1

1

1

3
40

6

6

6

23

8

7

4

4

1

2

1

1

5

5

5

5

26

8

8

8

8

3

3

3

3

15

2
15

2

1
2

1

4

4

7

7

27

2

2

1

1

1

1

1

1

6

6

6

1

1

1

5

2
5

3

19

9

9

1

1

1

1

8

8

5
1

2

2

2

2

1
3

1
2

1

1

10

10

10

6
2

4

4

3

1

4

4

4

7400
4

8

8

8

2

2

2

6

6

381
7388

5171
90

3887
5

29

11

11

11

11

6

6

6

1
6

1

1

1

1

3

2

2

1

1

1

1

1

1

1

5

5

5

2
5

3

5

5

5

5

5

5

14
3853

121

121

121
49

4

2

2

2

1

1

1

1

1

1

16

1

11

1

10

2
4

2

2

9
2

2

2

1

1

4

4

8

8

8

8

5

1

1

1

4

2

2

4

2

2

2

2

1

1

5

1

1

3

3

3

1

1

9

2

2

1

1

1

1

1

1

2
1

1

1

2

2

2

1

1

1

1

1

11

1

1

1

5

5

1

2

2

2

2

2

2

2

2

2

1

1

1

34
3718

13

13

13
3

2

2

1
4

2

2

1

1

1

1

1

1

2

1

1

55

55
4

6

6

1

1

5

5

13
3

3

3

3

3

3

4

4

1
6

3

2

2

2

5

5

5

5

8

8

8

13
6

1

4

4

2

2

180
3616

782
10

4
323

14

14

14

14

17

17

17

11
17

6

288
16

4
19

6

1

6

2

18

18

18

18

235
50

13
89

3

14

14

14

9

15

15

12

9

18

9

9

9

9

26
4

6
5

1

3

3

4

7

7

2

2

14
5

4

4

1

25

5

20

13

13

13

449
7

9
90

4
16

3

3

3

4

4

4

2

2

3
2

1

1

6

6

6

6

10

10

10

10

12

5
4

1

5
7

1

1

1

37
7

3

3

3

5

5

5

3

3

3

6
2

2

2

5
1

2

2

2
1

1

8

4
8

1

3

20
352

13

13

13
10

3

48

40

9
3

2

4

26

2

1

1
5

4

18

5

5

8

8

8

262
12

156
57

6

1

1

2

2

1

3

3

3

3

3

2

2

1

1

4

1

7

1

1

6

6

1

1

1

1

1

20
1

1

1

5

4

4

4

5
4

1

1

1

1

1

2

2

5

1

3

1

1

1

1

1

2

1

1

12
1

3

2

2

1

1

5

5

1

1

2

2

1

4

3

1

7

7

7

2

2

14
85

41

1

5

1

7

3

3

1

1

1

1

1

1

1

1

3

1

9

9

9

1662

1662
82

14
407

63

19

19

19

19

44

44

44

19

11
6

3

3

2

2

8

8

8

8

48
2

18

18

18

13

13

13

15

15
8

2

3

2

16

16
1

10

10

5

5

5

247
19

11

6

6

6

5

5

3

3

3

3

92
9

1
17

1

1

7

6

1

5

1

3

3

1

3

3

9

4

4

4

5

5

5

3
13

4

4

4

3

3

3

3

3

3

3

9

4

4

4

5

5

35
1

4

4

2
7

5

5

5

1
7

3

1
3

2

3

2

2

2

4

4

3
5

2

7

7

7

2

2

2

11

11

11

11

1

1

1

1

5

5

5

5

5

5

5

5

4

4

4

4

13

10

10

3

3

2
48

13
3

1

1

1

5

5

5

4

4

3

3

3

17
2

5

3

2

3

3

3

7

7

7

7

7

7

6

6

6

8

8

8

12

12

12

12

6

3

3

3

3

3

6
280

9

9

9

9

20

20

20

20

2
86

60
16

9

9

16

16

16

5

5

5

2
4

2

10
1

9

15

15

15

15

9

9

9

9

31

31

31

31

12

12

12

12

12

2

2

2

16

16

16

16

10

10

10

10

10

21

21

17

17

17

17

26

26

6

6

6

6

18

18

18

18
893

34
678

34
296

24

24

24

24

24

18
38

4

4

6

4

6

28

28

28

28
18

2

8
7

1

30
58

9

2

6

11

11

62

62
1

61

21

21

21

21

327
11

25

25

48
9

20

19

94

94

31

31

31

51

51

51
31

20

67
3

24

39
40

1

1
53

14

14

14

38

38
7

6

6

13

7

7

5

5

40

30

30

30

30

10

10

10

10

2
71

7
50

12

12

7
31

6

5

13

19

19

19

19

33

33

33
22

11

22

22

22

22

22

22

12
878

299
9

82

82

1

1

1

1

71
2

68

1

8

5

3

1

1

161
19

19

11
5

6

8
1

3

4

4

4

9

9

9

16
3

2

2

11

6

5

5

17

3

3

14

14
3

3

3

1

1

7

7
18

3

2

2

1

1

3
1

1

1

3

1

1

1

1

2

1

1

11

4
11

1

1

3

2

1
15

2

2

2

4

8

8

8

2

2

12

4

4
3

1

8

8

8

8

8

6

6

6

9

9

9
3

1

2

3

1
47

13

4

4

9

18

18
3

8
15

1

1

1

4

15

6

6

6

9

9

9

21
567

133
4

1
11

5

5

5

5

5

5
3

1

1

1

10

10

1
10

3

1

1

5

5

7

7

7

7

7

16

16
2

3

3

3

3

3

3

4
8

4

4

61
1

2

2

2

2

2

48
1

2

2

45

38
35

3

3

4

6

6

6

4

4

3

1

24

3
7

4
2

2

2

2

2

2

15

2

2

2

3

3

3
4

1

1

6

3

3

3

3

3

5
302

16

7

4

4

3

3

3

9

4

4

5

5

55
1

43

43
8

4
2

1

1

3

1

1

1

3

3

2

2

5

2

3

3

6
3

1

1

1

1
6

1

1

1

1

1

1

1

4

4

11

3

3

3

5
8

3

20
226

22

20
8

2

3

3

1

1

5

1

2

2

7

7

7

6

6

6

6

36
2

5

5

15
4

1

2

2

1

1

4

4

3

3

3

3

3

3

3

3

3

3

2

2

3

3

2
27

13
4

3

2

3

1

3

3

3

6
1

2

2

2

1

2

2

1

1

1
76

1

1

3

3

71
14

2

2

2
6

2

1

1

2
5

2

2

1

1

3
1

1

1

1
5

1

3

2

2

2

8
13

3

2

5

5

4

4

3

3

5
1

4
1

1

1

1

1

1

4
1

1

1

1

11

11
8

1

1

2

1
21

2

2

5
2

1

1

1
2

1

13
2

5

5

2

2

2

1

1

111

2
111

9

9

9

10
29

7
11

2

2

2

8

8

8

18
5

7

1
7

2

4

6
1

4

1

1

25

6
17

11

1

2

1

2

4

1

8

8

19

9
1

2

1

1

3

1

1

10

10

2

2

2

2

7

7

7

92

92
5

40

1

1

1

1

1

1

1

1

38

3
38

33

1

1

2

30
22

6

2

2

2

1

1

1

43

43

43

43

43

4

4

1

1

3

3

3

2

2

1

1

5
1194

2
853

2
205

6

6

2

2

2

2

1

1

3

3

197
2

16

16

16

10

10

1

9

12

1

1

11
6

1

4

4

146

137

3

131

1

130

1

1

2

2

1

1

1

1

2
9

1

1

3

3

3

11

11

11

433
5

130

119
1

62

62

62

62

3

1

1

2

2

3

1

1

2

2

2

1

1

1

1

1

1

5

3

3

2

2

2

2

1

1

1

1

1

3

3

3

3

1

1

1

1

1

1

1

5

4

4

4

1

1

3

1

1

2

2

1

1

1

9

9
1

1

1

2

1

2

2

4

4

1

1

2

2

1

1

2

2

1

1

1

5

3

1

1

2

1

1

1

1

6

3

1

2

3

3

3

1

1

1

1

1

1

1

1

1

1

9

1

1

1

3

3

3

1

1

2

2

1
3

2

2

1

1

2

2

2

1

1

1

1

296
1

2

1

1

1

1

1

1

1

8

1

1

1

1

1

1

6

2

2

2

4

4

7

3

1

1

1

2

2

1

1

1

1

3

1

1

1

1

2

2

131

1

1

1

3

3

3
1

1

1

90

90

90

90

31
1

2

2

1
5

3

1

1

4
23

19

19

1

1

1

1

3

1

1

1

2

2

2

2

2

1

1

1

135

31

30

30

30

1

1

1

101

98

71
98

27

27

3

3

3

3

3

2

2

2

2

2

6
1

2

2

2

2

3

3

3

3

4

4

4

4

4

2

1

1

1

1

1

1

1

1

1

213

213

1
213

212
1

211

211

8

8

8

8

8

1

1

6

130
2

1
103

1
60

50

50

50

9

9

9

1

1

1

1

19

19

11
19

7

1

18

18

1
18

17

2

2

2

2

2

2

2

1

1

1

1

17

1

1

1

1

16

14

1

1

1

6

4

2

5

5

5

1
2

1

1

2

2

2

8

8

8

1
8

3

4

4

198

16

2

2

2

12

12

12

3

4

5

2

2

2

2

180

151
180

18

12
18

2

2

2

2

2

2

11

11

1

10

10

2

2

2

2

2

2

1823
28

33

16

6

6

6

3

3

3

3

3

9

1
9

8

8

3

2

2

1

1

1

1

1

1

5

5

5

3

3

3

2

1

1

1

1

12

7

7

3

3

3

4

4

4

4

5

5

5

5

5

5

5

5

5

5

3

3

3

3

2

2

2

2

124

124

64

64

56
64

7

5

2

1

1

6

6

6

6

6

6

3
54

3

3

3

15

15

15

6

9

15
1

6

6

6

8

8

6

6

6

4

2

9

9
1

4

4

4

4

3

3

3

3

1589

2
226

3

3

3

1
3

1

1

8

8

2

2

2

2
6

1

1

1

3

3

213

213
1

154

83

83
29

43

5

6

71

8

4

4

56

56
1

53

2

2

2

5

5

26

24

24

2

2

3

3

3

3

29

24
29

1

1

4

38

38

38

3
38

3

1

2

4

1

3

1

2

1

1

1

5

4
3

1

1

16

15

1

1

1

1

1

2

2

2

2

1325

1325
2

1172

1172
1

308

308

6
863

461
323

63

75

29

29

367

151

147

147

147

4

4
1

2

1

16

16

10

10

10

6

6

28

12

2

2

2

2

3

3

3

3

4

3

1

16

16

16

13

3

13

1

12

1612

1612
107

36

36

36

36

2
102

1
35

4

4

1
2

1

2
1

1

1

29
1

1

1

1

1

21

4

1

1

1

1

2

2

2

1

1

17

17

8

8

9

6
1

5

5

1

1

1

1

1

7

7

7

7

7

58
4

7

7

7

7

47
1

38

38
24

1

1

4

6

2

8

8

8

27

27

27

1

1

26

26

1

1

6

6

6

6

6

45

45

45

45

32

13

17
1023

693
7

9
675

61
4

3
24

13

13

8

8

2

2

2

25

24

24
6

13

5

1

1

6

5

5

1

1

28
605

27
2

12

12

13

1
13

12

502

4

4

3
498

16

16

452

1

26

6

6

6

18

2
18

15

1

7

7

7

17

17

17

11

11

11

11

1

1

1

1

1

28
312

3

3

1

1

2

2

59

53

3

3

50

50

50

6

3

3

1

1

2

2

1

1

1

1

59

59

11

4

7

47

47

47

1

1

162

2
162

34

34

8

8

7

1

115
6

89
3

20

52

52

12

2

20

20

1

1

2

2

10

10

10

9

9

9

1

1

6

1

1

1

1

1

5

5

249

7
248

47

21

21

10
26

10

6

6

6

194

194
4

157

157

33

1

1

1

1

2

2

2

2

2

58

51
2

11

11

11

11

11

38

1

1

1

1

1

1

2
37

22

1
7

2

2

4

2

2

15

15

15

2
13

10
2

2

2

2

6

6

6

1

1

1

7

7

7

7

7

7

7

53

6

4

4

4

1
4

3

2

2

2

2

46

46

46

46

46

1

1

1

1

1

1

110

1

1

1

1

108

1

1

1

1

107

107

1
107

106

1

1

1

1

18

18

18

6

6

6

6

12

12

12

12

12

8

2

2

2

6

6

6

6

10287
377828

90

1

1

72

15

1

1
22

18

14

2

2

12
2

2
1

1

3

3

1
5

4
2

1

1

4

4

4

1

1

2

2

2

1

1

1

1

1

1

1

6

2

1

1

1

1

1

1

1

1

4

4
1

2

1

1

1

1

1

1

1

5

5

1

1

1

1

1

2

2

2

2

1

1

1

1

1

1

1

4

4

4

30212
390

2

2

3

3

447

36
442

10
65

1

1

1

13

1

1

12

12

1

1

4

4

2

20

12

1

1

1

3
9

1

1

4

1

8
1

7

2

2

2

16

1

1

1

1

14

8
1

2

1

1

1

1

1

3

3

3

3

2

1

1

1

1

1

1

1

115

10
75

1

1

1

25

1

1

18

2

2

16

4

4

4

2

2
1

1

16

1

1

14

10

4

1

1

1

1

1

1

1

1

21

4

2

2

2

2

2

15

15

15

3
40

5

2

2

1

1

2

2

2

2

2
1

1

30

30

30

113

1

1

106

2

1

2

1

1

1

1

108
5

19

1
19

1

1

14
1

13

2

2

10

3

3

7

1

1

6

6

1

1

1

1

1

1

1

9

9
5

1

2

1

28
1

24

24

24

2
3

1

1

33

1

1

1

32

1

31

31

2

2

2

2

2

2

2

1

1

1

1

1

5

1

1

1

4

4

9

3

2

2

2

2

1

1

1

1

6
3

1

2

4
174

4

2

2

2

2

2

2

2

2

2

74
2

1

1

1

1

57

10

1

12

10

10

10

1

1

8

1

7

7

1

1

1

1

1

32

17

2

1

1

8

1

1

7

1

2

4

4

1
4

3

3

3

3

3

15

15

15
1

14

1

1

1

3

2

2

1

1

1

1

1

1

1

14

8

5

2

2

3

2

1

3

3

6

6

29

1

28

82
24850

214

2

2

2

2

212
3

44

20

18

6

33

3
33

9

14

7

3
114

2
98

7

78

71

7

11

13

13

18

2
18

5

10

1

15

1

1

1

1

1

14

6

2

2

4

3

1

2

1

8

8

8

24456
1486

26

7

7

7

19

19
2

3

6

8

57

2
57

39

39

1

1

4
15

1

2

2

4

1

3
2

1

99

99

6
99

35

16

4

15

3

6

5

8

9

4

11

12

3

3

3

3

18

18

12
2

1

1

2

1

1

2

1
2

1

5

2

2

1

1

1

3588

3588
57

2
29

1

6

3

9
1

1

1

3

2

1

2

1

1

4

3

3

3456
515

19

2

1

1

1

1

1

64

2

1

1

1

8

1

2

2

25

75

2

1

11

1

186

3

3

1

1

1

1

1

1

2

1

1

6

1

2

1

1

2

1

1

1

1

1

1

1

2

1

1
2

1

5

3

1

1

3

1

2

1

1

74

5

53

1

2

1

2

3

4

1

2

2

1

2

1

1

2

1

1

1

1

1

2

1

2

1

2

1

2

3

1

1

3

3

1

1

2

2

1

2

1

3

1

24

141

1

1

1

1

1

2

1

2

1

1

1

56

1

1

1

1

1

2

1

948

1

1

1

1

8

4

2

1

2

3

1

1

1

1

2

1

1

1

2

1

1

1

1

3

1

1

1

109

1

2

1

1

1

2

117

1

1

2

1

1

2

1

1

153
696

1

1

1

1

1

2

1

2

1

1

3

3

1

1

1

1

1

1

1

105

1

2

3

1

1

1

1

1

5

1

1

1

1

1

1

1

2

3

1

1

1

1

2

1

4

1

1

1

1

1

2

1

2

2

1

1

171

1

2

3

1

7

1

1

2

1

1

1

1

1

1

3

1

1

1

1

1

1

1

1

2

2

1

1

2

1

1

1

1

1

1

1

1

1

4

1

1

1

1

1

1

1

1

1

2

2

1

1

1

3

2

2

2

1

1

1

1

2

2

1

1

1

1

4

1

37

1

1

1

1

3

2

1

1

2

1

4

2

2

2

1

1

2

1

1

1

2

2

1

1

1

2

1

2

1

1

1

1

1

1

1

2

1

1

83

7

3

1

1

10

2

1
5

2

2

36
5

2

3

1

4

3

3

3

2

10

4

4

2

2

1

1

2527

2527

2527
15

2318

194

133

61

53

53

1
23

3

2

4

6

6

7

4

4

26
8

1

8

2

2

1

2

2

126
2

1

109

1

1

1

8

1

2

31
1561

257

178
257

1

1

1

1

1

1

4

1

3

1

1

1

1

1

1

1

1

2

2

1

4
3

1

1

1

1

2

5

35
18

1

1

1

1

3

1

1

4

3

1

1

3

1

1

1

17

12

12

5

1

3
4

1

3

3

132
707

6

4

2

69
14

1

41
23

2

14

2

1

1

11

19
115

1

2

7

1

2

3

1

1

1

2

5

3

1

2

2

2

1

4
20

2

3

4

1

1

1

3

1

1

1

3

1

3

2

1

1

1

1

4

2

1

1

3

1

4

1

1

5

1

34
367

1

1

4

3

3

253
11

1

1

1

1

2

2

1

4

1

1

6

1

3

1

1

1

1

1

1

2

1

1

1

2

173

1

1

1

3

1

1

4

1

1

4

4

1

1

3

1

1

1

1

4

2

1

1

1

2

1

1

7

7

4

2

1

6

2

1

1

1

9

2

2

2

1

1

1

2

2

1
12

1

4

2

1

2
1

1

1

1

1

2

1

1

3

1

1

10
5

1

3

1

7
3

1

1

2

1

3
95

4

4

80
20

3
14

1

6

1

1

1

1

1

2

1

1

2

1

3

3

2

1

1

2

2

1

2

1

11

1

1

2

3

2

1

1

7

6

6

1

10

10
4

1

1

3

3

1

424
2

185
34

1

1

2

8

2

2

2

2

2

1

1

4

1

1

3

3

1

23
1

4

1

1

1

2

3

2

1

1

1

4

1

1

4

3

1

1

8

3

1

7

2

1

1

2

1

3

1

1

1

3

5

2
1

1

1

1

1

1

1

1

2

3

4

1

1

1

2

2

1

2

2

7

5

13
1

8

3

5

4

7

7

60
193

1

2

3

1

1

4

86
16

3

7

1

12

1

1

1

2

2

3

2

3

2

1

5

1

1

1

2

3

1

1

1

1

5

1

1

1

2

1

1

6

1

3

1

4

5

2

5

2

2

4

24
7

1

2

1
3

1

1

1

10

12

12
5

1

1

1

2

2

4

1
4

2

1

7

7

7

5986
137

27

7
2

1

1

2

2

1

20
3

1

1

2

8
2

1

3

2

2

1

2

16

14
1

1

4

1

2

3
4

1

1

2

2

834
31

1
2

1

4

4

3

2

1

18
9

1

1

1

1

1

1

3

1

2

1

2
4

1

1

1

2

1

1

10
269

1

2

234
9

1

1

7

1

1

2

1

11

1

4

1

1

2

1

2

1

2

1

1

1

1

2

4

1

2

1

2

33

1

1

1

1

123

4

2

2

1

2

3

1

1

1

2

1

2

1

2

3

3

2

2

3
1

1

1

1

9
1

2

2

1

1

1

1

1

1

1

7
42

17

5

13

13

2

2

1

1

411

410

1

13

6
1

1

1

2

1

2

4

1

16
2

1

1

2

1

1

5

1

3

1

2

1

15

4

4

1
2

1

1

1

1

8

2

6

16

16

11

5

1

1

1

7

7
2

2

2

1

1

1

2

2

1

1

2

2
1

1

60

56
4

4

1

1

6

1

1

1

1

1

1

1

35

1

1

2

1

1

3

3

6

1

2

3

22

5
22

1

1

1

1

3

1

1

2

1

1

1

2

2

1

14

11

4

7
2

2

1

1

1

3

1

2

40

24

24

2

2

2

2

6

3

2

2

1

6

2

2

4

2
52

3

3

16
5

1

2

3
2

1

1

2

2

1
4

2

1

1

1

6
12

1

1

1

3

1

2

3

3

2

2

1

1

1

1

1

2
8

2

4

386
4086

2

2

2
22

6

5

7

1

1

1

4

2

4

3

3

1

23
9

1

5

2

2

1

1

2

2

3

6

6

1
3

2

3

3

3

5
4

1

1

27
4

6

6

5

4

8

14

13

13

1

2
3

1

10
3

4

2

1

16
75

2
3

1

6

10
9

1

1

1

1

38
7

4

2

3

1

4

1

4

1

3

3

1

1

2

1

2
5

2

1

3

3

11

3

8

2
20

4

14
5

2

1

3

1

1

1

22
4

1
2

1

13
16

1

2

25
63

2
27

21

4

11

29
8

1

3

1

1

13
5

1

1

1

1

1

1

1

1

2

1

1

1
8

2

5
1

3

1

3

3

51
5

2

1

13
31

1

1

1

1

1

2

1

1

2

1

1

1

1

1

1

1

1

6

1

4

9
46

5

15
32

1

5

2

2

3

1

1

2

13

7

6

1

5

18

6

5

1

2

1

1

3

88
584

7

288

201

1

1

10
1

5

4

10

10

4
73

28

1

1

1

3

1

3

1

1

2

3

3

2
20

1

1

1

1

2

2

1

1

1

3

1

1

2

1

2

2

7

7

9

5

5

4

16

10

10

6

46

37

8

1

1
13

6

6

306
62

2

1

1

1

19
142

3

1

1

4

1

1

2

2

1

1

2

3

1

2

1

2

1

1

1

1

53

5

2

1

1

1

4

1

2

1

1

1

2

1

1

1

1

3

1

1

2

1

1

1

1

1

3

1

5

2

1

2

1

5
4

1

1

1

4

2

1

2

1

3

1

1

1

3

2

1

3

5

1

2

2

1

1

3

3

1

1

1

1

1

1

1

1

1

1

21

2

1

4

4

8

3

3

5

2
14

3

5

4

4

3

3

5

2

1

1

2

1

13
2

3

2
4

1

1

1

2

1

8

4

4

4

4

4

5

3

2

2

1

1

65

13

9
52

1

5

9

17

11

4

4

4

663
1985

83

34

2

3

31

505
1081

2

1

7

12

3

102

2

3

4

5

9

1

17

12

3

1

3

2

3

3

2

9

8

3

2

1

4

12

4

9

1

2

15

31

2

10

8

8

1

16

4

4

17

63

1

1

5

1

2

32

1

1

8

3

2

16

9

6

1

3

3

11

1

12

2

2

2

1

2

2

15

10

78

19
1

1

1

1

3

1

1

1

5
9

1

3

7
200

9

9

3

3

26

26

54

3

51

36
4

5

12

8

7

10
13

2

1

2

2

2
16

7

7

7

11

11

10

3

7

13
6

1

1

6

29
298

3

3

9

9

22
2

1

3

10

3

7

2

3

1

7

3

2

2

13
2

1

10
1

1

1

2

4

1

36

6

1

1

4

10

16

4
2

2

9

9

23

5

5

5
9

1

1

2

4

7

7

66

66

6

1

5

8

6

2

2

11

4

3

4

2

2

5
27

3

1

1

1

1

3

1

8

4

3
20

6

11

2

9

38

28
12

1

3

1
4

1

2

1

1

1

1

3

1

3

3

7

7

113
2

12

1

1

3

1
4

3

3

1
2

1

1

1

2

2

5

1

1

4

89
15

3

1

2

1

2

1

1

2

1

1

1

1

1

42
3

1

2

2

1

2

1

1

2

2

2

2

2

1

3

1

2

1

2

2

1

6

2

1

3

7

1

18

18

7

7

11

11

1072
1

44

41

3

1023

1023
126

251

2

344

145

10
148

3

1

1

3

2

35

2

77

7

2

2

2

1

4

1

2

4

4

686
136

11

11

11

5

5

5

13
192

3

3

23
9

2
7

1

1

2

1

1

1

1

1

3

2

2

66
13

9

4

1

1

2
10

1

4

1

2

3

1

2

1

2

1

2

1

2

2

2

3

6

2
14

1

1

1

1

2

2

4

4
11

5

2

2
9

2

2

1

2

1

1

6
1

4

1

9
7

1

1

1
4

3

1

1

4

4

1
8

1

1

5

4
3

1

1

3
1

2

6

6

1

1

5

5

248
3

226
23

1

1

2

1

3

4

2

2

2

1

2

2

2

19
1

1

1

3

1

1

1

2

1

1

1

3

1

1

5

1

1

1

3

1

3

1

2

2

127

1

1

1

1

3

2

1

2

7
1

2

2

2

1
4

1

2

8

8

94
4

6

2

2

1

1

1

7

1

2

4

6

6

53
10

3

1

2

2

4

1

1

1

1

10

1

1
13

1

2

2

3

2

2

1

2

5

5

2

2

3

3

2

2

1

1

4

4

1

1

1
295

290
38

68
13

7

2

6

2

1
17

7

1

2

3

2

1

2

2

1

1

3

1

6

1

2

2

5
61

6

5

10
28

6

4

7

1

5

5

4

3

8
96

4

2

1

2

11
65

1

4

4

2

1

3

3

3

3

3

2

3

1

2

2

3

2

6

4

1

1

1

1

5

6

2

6

2

4

2
21

1

5

7

6

4

4

4

2177
25

92
1722

783
135

3

6

3

2

1

7

7

7

2

4

7

1

12

1

10

2

13

1

4

9

3

5

1

1

2

3

6

11

3

1

6

3

3

5

8

6

12

3

6

10

5

8

1

1

3

7

5

108
308

2

13

6

6

13

5

3

1

2

1

3

10

2

1

3

1

6

3

3

1

3

1

5

4

3

6

5

1

3

1

2

5

1

9

3

1

4

1

2

20

5

2

9

4

3

6

6

4

1

7

10

4

3

9

13

2

4

3

10

4

5

3

1

3

3

3

7

2

9

7

28
80

4

2

2

8
24

1

2

1

3

2

1

2

3

1

3

2

2

1

6

2

1

1

2

19

18

1

1

6

6

115
2

100

2

2

11

3

3

4

4

3

3

5

5

13

13

30
584

14

49

14

35

7

227

20

12

20

185

14

6

15
8

4

3

2

1

104

104
25

5

2

13

1

2

2

37
4

1

2

2

1

2

2

1

3

1

2

8

3

1

4

1

1

3

7

3

1

1

310
16

3

3

2
14

1

1

2

8

5

1

1
2

1

1

1

1

14

6

8

4

4

3

3

6

2

4

4

3

1

1

1

183
20

8

5

23

13

13

1
16

6

9

29

6

36

14

4

3
4

1

4

4

2
17

1

2

1

1

1

3

2

1

1

4

2

6
1

1

4

2

2

9

5
3

2

4

4
1

3

9
5

2

2

16

16
2

3

7

4

29

29

2
29

6

16

5

3769
7

3388
139

1
23

4

7

6

5

10

1

9

2
13

4

7

2
14

9

3

3

10

10

14
27

1

1

3

1

2

3

1

1

2

1

1962
533

276

300

361

361

44

44

257

191

7

7

5

5

6
36

5

4

8

3

5

2

8

3

7

3

4

18
91

4

7

1

21
1

3

4

1

2

6

2

2

7

6
5

1

8

2

1

8

1

2

4

1

12

9

3

3

45
12

1

4

2

2

3

3

1

3

3

8

3

2

6

6

6

230
53

7

1

2

3

5

2

2

2

5

5

6

1

1

2

5

1

2

2

1

1

1

5

1

3

4

5

2

35
1

1

1

1

2

5

9

1

2

1

1

1

1

6

2

3

2

1

1

7

3

1

5

1

1

1

3

1

2

8

7

9

2

4

3

1
14

4

4

2

3

3

5
21

2

5

7

7

2

78
569

11

9

23

2

9

37

14

22

25

7

1

1

13

11

9

5

23

20

8

55

164
35

4

1

13

1

15

2

6

1

2

16

2

15

1

17

4

3

2

14

1

1

8

11

12

4
7

2

1

1

5
15

6

6

4

6

6

6

6

6

41
5

2

6
1

1

1

1

2

1

1

5

2

5

3

8

2

1

6
15

4

2

3

4

4

5
29

1

1

5

5

1

2

2

1

3

2

1

5

12
2

2

2

5

3

4

4

8

8

374

374

19

12

12
1

3

1

1

1

6

7

7

7

617

27
617

6
15

1

5

2

1

2
17

6

2

4

2

1

1

1

2
18

1

11

4

3

3

17

3
12

3

3

3

2

1

2

3
1

1

1

5

1

1
4

1

1

1

7

7

4

4

5

5

6

6

7

6

1

277
24

3

5

5

1

3

4

2

1

4

6
36

6

3

1

2

2

1

4

3

8

3

1

3

4

3

1

1

147

3

1

3

3

2

3

3

4

2

2

5

2

2

3

3

2

1

3

3

2

2

1
12

1

4

2

2

2

5
23

5

3

6

6

4

1
18

4

6

7

9

9

19
3

11

4

1

3

3

98
31

1

2
3

1

2

1

1

1

2

1

1

2

3

1

1

1

1

1

10
30

2

1

1

2

3

1

2

1

1

1

2

1

1

1

1

1

1

2

1

2

1

2

1

1

1

2

2

8

8

20

20

20
12

1
2

1

1

1

2

2

4

4

4

1

1

1

1

146

1
146

6

2

4

11

11

11

24
103

6

11

7

10

15
14

1

12

18

16

11

6

2

2

1

2

3

3

9

2
9

3

4

67
1

3
47

1

15

1

1

14

14

1

1

1

1

1

1

3
14

1

1

1

1

4
1

2

1

1

1

1

1

1

1

1

2

2

1

1

1

1

1

1

1

1

1

1

1

2

2

19

1

2

12

4

30

6

1

1

9

6

1

1

1

1

1

2

23

1

1

1

1

1
22

20

19

2

16

1

15

1

1

1

1

1

1

1

22

21

6

3

3

1

1

2

2

1
6

5

2

2

1

1

3

3

4

4

2

2

2

1

1

8

2

2

2

2

1

1

1

1

4

4

4

3

1

1

1

1

1

4193
16

47

24

14

1

2

2

1

1

10

10

10

2

2

1

1

1

1

6

6

11

11
1

2

1

1

8

2

6

6

12

12

12

12

6

1

1

1

2

1

7
3296

594
1

66

66
1

65

3

3

3

312

312

19

19

274

15

1

3

32

1

31
1

4

4

4

4

1

1

1

15

1

8

180
3

5

5

1

1

9

9

2

2

2

1

1

5
7

2

2

139

37

102

1

1

11

11

76
2695

17
1

16

16

1528
3

1

1

2

1

1

1

1

1

2

2

2

2

2

2

1

1

1

1

14
1364

1

1
101

100

40

60

24

1

1
725

1

6

1

1

1

2

4

482

166

21

38

1

219

3

270
3

3

111

146

7

2

3

1

2

2

2

1

1

1

1

96

96

10

3

5

1

1

1

1

1
7

3

3

2

1

29

16

16

7

6

1

5

3
4

1

1

199

199

197

2

765

459
3

408

17

4

27

173

173

131

131

1

1

1

1

14
2

2

2

1

1

3
1

1

1

5
6

1

11

1

1

1

1

8

6
8

1
2

1

2

2

2
80

1

1

1

2

2

1

1

1

1

1

1

1
5

1

2

1

1

1

3
1

1

1

13
65

1

1

25
1

1

1

1

4

8

1

3

1

1

1

1

1

4

1

1

1

1

1

1

10

1

1

1

1

1

1

1

44

44

43

1

1

1

35

35

5

5

2

2

1

1

51

51

48

46

46

1

1

1

1

1

3

3

3

3

730

3

1

1

1

1

1

1

1

1

1

1

218
21

1

1

4

2

1

1

1

1

1

1

1

1

1

1

8

1

1

7

7

86

83

82

1

3

3

37
17

1

1

1

1

1

6

6

6

2

2

6
5

1

1

1

3

3

1
7

1

1

1

2

1

1

2

1

1

1

1

1

1

19

3
15

1

1

1

1

4

1

1

2

1

3

1

1

1

2

2

1

1

1

1

1

1

1

1

1

1

1

32

4

4

1

1

2

2

1

1

1

1

1

1

4

4

13

11

1

1

5

5

1

1

1

507

505

505

505

1

1

1

1

1

1

1

2

2

2

2

2

1

1

2

2

2

2

2

2

41

40

11

11

11

1

10

10

28

28

28

28

1

1

1

1

1

1

1

101

101

99

99

99
40

1

1

1

1

1

1

1

1

2

2

1

1

8

2

1

2

2

2

1

1

10

2

1

1

1

1

1

1

1

1

2

1

2

3

1

4

2

1

2

2

2
1

1

88

1

1

87
2

1

1

1

2

1

1

1

1

1

1

1

1

1

26

21

18

18

1

1

2
1

1

1
5

1

1

1

1

1

1

1

1

4

4
3

1

2

1

1

1

1

1

16

4
16

12

2

2

1

1

2

1

1

3

3

21

6
21

1

2

1

9

1

1

6

6

2

2

2

2

4

4

4

4

4

2

2

2

2

1

1

1

1

1

1
165

1

1

1

1

1

73
4

68

1

1
88

3

1

1

1

1

1

1

1

1

1

4

3

1

80

5
80

10
1

8

1

2

2

3

3

31

2

1

21

1

7

5

3

4

1

3

4

19

4

2

2

1

5

2

5

2

2

2

4

2

1

1

2

2

1

1

1

2

2

1

1

1

1

1

1
31

19
5

13

1

1

1

1

1

1

1

10

10

10

2
10

3

1

2

1

1

2

3

3

3

3
2

1

244

2

2

1

1

8

2
8

1

4

1

1

1

2
122

1

1

1

1

29
1

17

2

3

1

1

2

1

1

84
2

40

40

22

22

3

3

9

9

1

1

4

4

4

1

1

18

1
18

2

1

1

8

7

7

1

3

3

2
4

2

13

9

1

2

1

1

1

1

1

1

2

1

1

1

2

2

4

1

1

1

1

1
2

1

11

11

2

2

1

1

2

2

1

1

1

1

4
1

1

2

6

1

1

2

2

2

2

3

2

1

1

100
7

13

4

4

2

2

1

1

1

1

1

1
2

1

7

7

5

5

1

1

1

11

11

7

7

4

4

4

29

23

1

1

20

3

1

2

8

8

7

2

2

5

2

1

1

2

2

2

6

5

1

39

7

1

27

1

2

1

1

1

1

1
11

1

1

9

3

1

1

1

1

1

1

1

1

1

6

1

1

2
5

3

3
1

1

1

330905
6303

1

1

41
1

5

1

2

2

29

1

1

1

22

22
1

20
16

2

1

1

1

1

1

560

17

1

1

16

1

1

14

10
1

1
8

7

7

1

2

2

2

1

1

1

1

1

1

1

543
10

46

6

1

1

1

1

1

1

1

1

1

1

1

2

1

1

40

3

3

3

3

25

3

22

22

1

1

1

7

1

1

5

1

1

1

1

1

19
1

13

1

1

1

11

8

2

1

1

1

1

1

5

2

1

1

1

3

3

1

1

1

119

32

22

5

3

2

12

12

5

2

3

5

5

5

3

3

3

2

2

2

2

1

1

1

1

1

1

1
78

7

7

7

4

3

23

5

3

2

3

1

2

10
7

3

5

5

47
2

30
2

1

1

5

9

12

11

1

2

2

5

5

8
1

1

1

6

7

1

5

1

4

4

2

2

2

1

1

1

1

5

1

1

1

1

1

3

1

1

1

1

1

1

1

1

9

8

8

1

1

1

1
290

286
2

1

1

6
273

262

2

260

1

1

1

1

1

9

9

1

1

7

1

1

2

1

1

1

1

1

1

1

1

39
3

1

1

18

1

1

1

1

5

4

2

1

1266
298419

160
11

2

1

1

1

4

1

8

3

1

3

1

1

91

1

1

1

5

2

4

2

9

1

3

1

1

32

32

3430
296036

125900
288175

348
972

16

2

21

9

23

28

17

17

22

17

9

5

18

14

5

23

11

47

17

7

28

6

2

80
2

19

3

2

12

38

4

170

2

8

32
4

7

5

10
3

3

2

2

2

1

1

1

1

36386
5715

373

60

273

97

818
1612

794

24

136

516

81

115

306

48
52

4

186

387

86

555

111

31

404

167

10

94

82

160
255

95

100

206

122

362

130

132

110

7328
660

132

39

288

271

26

11

30

102

113

19

34

27

76

337

190

55

22

67

58

68

53

266

5

14

64

76

105

679

10

52

463

301

129

331

244

69

330

181

155

159

225

287

3

114

321

12

38

17

7

170

68

653

712

160

99

116

265

6

344

139

146
138

8

326

86

519

232

64

132
127

5

128

234

251
271

16

4

364
351

13

195

270

104

29

126

5391

164

99

270

328

50

728

1903

136

106

123

96

158

190

67

137

27

230

5

1

4

5384
50498

12359

3990
404

209
224

6

9

461

70

24

17

115

71

65

46

23

430

8
19

11

722
736

1

8

5

27

289

12

82
123

41

73

7

142

333
368

2

30

3

8

236

92

164
158

6

13007
1226

17

11

12

3

4

9

10

91

172

316

22

1

22

11

3

116

20

30

1

4

134

8

6

30

10

2

2875

2

1

3

6

3

1

3

2

69

3

3

22

497

1

46

70

3

242

196

87

6

48

5

26

10

3

18

18

3

43

3

2

93

6

11

502

381

43

1

4

3

340

187

496

1

5

1304

9

2

5

10

74

2

148

105

2

111

18

23

4

10

1

6

2

1

29

267

3

16

28

4

1

485

5

136

6

31

60

2

2

25

1

28

65

74

8

12

31

1

2

81

494

199

321

54

15

4945

150
707

3

243
289

2

1

2

4

30

4

2

1

1

2

108
89

19

2

4

1

9

23

1

48
40

6

2

33

28

5

1444

293

2914

4759

401
398

3

12

27

3
308

145

160

7

5

5

2

1194
63

277

95

163

215

157

157

9

101

114

4
106

35

50
67

17

1

1

70685
16761

1294

1656

141
142

1

227

2926

3815

432

360

2628

3358

163

3327

857

1400

1073

2765

2636

3812

145

3699
189

18

713

14

2765

2572

2237

1141

3913

378

1363

4809

796

6

6

62

7

10

24

21

9

9

2

2

3
7

4

1

3

7

7

7

8

2

2

1

3

2

16

7

7

9

452
165

5
91

5

30

14

4

9

15

2

7

106
102

2

1

1

8
11

3

5

41
57

15

1

9

8

209

209

529
1303

27
29

2

46

11

6

28
32

4

25
211

1

2

15

4

2

19

16

2

2

4

15

9

27

10

3

18

14

7

1

11

4

12

102
99

3

21

83

87

15

14

74

31

1
46

21
3

15

4

11

3

3

18

18

18

6

6

80
1384

23
93

3

15

9

2

6

1

1

3

1

6

4
16

1

5

3

1

2

4

3

15

1

7

1

4

2

7

7

4

2

1

14

1

1

3

10

8
12

4

4

4

4

4
56

2

2

6

20
42

3

4

2

1

3

1

7

1

1

1

2
14

1

1

11

395
71

4

3

3

16

261
68

4

3

5

5

1

16

8

10

1

2

4

9

4

3

1

7

4

3

2

11

6

3

2

10

4

11

52

2

37

4

4

4

3

3

1

2
35

4

1

2
28

1

3

5

7

7

3

5
76

59

4

1
7

3

3

1

17

15

11

4

2

23
6

1

16

5

6

5

52
3

47

2

15

8

8

7

52
6

1
39

3

3

15

1

5

3

8

7

18

6

11

1

4
64

12
29

2

2

2

2

1

6

2

7

12

1

5

1

1

4

2

2

5

124
1

1

1

12

4

1

4

5

7

5
89

12

5

2

1

6

9

2

17

3

5

5

17

36
7

3
28

3

10

1

1

1

8

1

1

14

13

1

1

23

23

4

3

15

1

3

3

14

1

13

3

1

2

12

12

14
95

15

1

2

1

5

39
10

1

1

1

1

2

3

2

1

1

2

5

4

1

4

8

3

4

1

2

2

2

1020
50

80
14

1

1

2

8

13

2

34
2

1

1

10

7

5

5

3

5

9

4

4

3

2

5
3

1

1

1

8

4

4

7

7

2
8

6

293
41

1

47
49

2

2

1

16

11

1

2

1

9

8

7

4

48
4

8

4

3

8

21

2

18

31

17

8

3

8

1

3

1

18

2

16

7

7

1

1

1
40

12

17

10

7

10

21

7

14

14

1

1

33

10

19

18

1

4

6

6

1

1

11

11

11

1

1

1
77

25

13

10

6

4

28

3

3

10

10

77
288

49

3

3

1

8

1

9

17

1

17

14

17

69
17

4

3

3

12

1

6

2

4

5

2

4

6

2

13

13

29
8

3
16

1

5

7

5

102
1425

9

9

7

7

23
83

7

15
19

4

8
25

5

5

5

2

9

94
11

9

68
24

1

2

4

5

8

6

10

2

3

3

5

1

2

1

1

1

12

1

6

1

4

4

55

3

12

28

12

255
26

1

2

4

7

3

5

6

5

4

10

7

131
28

1

3

8

3

3

6

5

9

1

3

8

5

3

1

9

5

7

5

5

6

3

4

3

3

12

1

3

1

3

2

1

7

2

4

2

70
11

3

2

4

6

2

4

3

4

14
31

3

1

2

4

5

1

1

19

19

2

4

6

2

1

4

306
109

13

7

10

3

7

131
28

26

14

5

10

1

10

18

2

6

9

1

1

2

5

1

2

5
6

1

10

55

13

13

28

14

10

4

4

3

3

137
30

1

5

7

6

88
23

5

1

1

9

11

5

6

3

1

2

8

2

1

4

1

5

190
4

8

1

7

11

17

18

100

8

6

75

11

15

9

9

4

2

3

10

10

431
20

16

12

12

4

12

12

7

1

1

2

1

1

1

5

5

3
18

3

5

6

6

1

1

1

1
49

24

24
2

5

4

2

4

7

29
9

1

2

9

1

7
1

5

1

27

5

1

21

5

13

3

3

3

1

1

1

1
81

4

4

4

2

13

6

47
6

13

10

16

2

32
10

7

7

4

3

4

4

8

3

2

2

3

2
50

5

2

41

11

19

10

1

10

1

1

4

5

1

1

4

1

1

1

2

27

17

2

8

17

2

8

7

2

4

1

17
2

11
3

4

4

2

2

3
125

3

1

1

1

1

3

3

40

1

1

8

4

3

5

1

28

2

14

2

6
377

10
300

9
27

8

1

9

1

8

6

3

3

19
2

1

16

64

58
8

7

2

2

6

23

11

1

6

2
4

2

2

10

5

5

6

6

2
8

2

4

2

2

17
23

2

1
4

3

4

4

25
6

4

1

2

1

5

6

6

2

21

9

9

5

7

43
17

10

6

10

19
6

8

3

5

5

8

4

4

5

2

3

3

4
71

1

1

1

2

2

1

12

12

5

4

1

10
11

1

1

1

7

7

3
12

1

1

2

1

2

2

2

6
15

3

6

1

1

1

1

284
22

113
6

57

3

9

1

40

2

3

2

14

4

7

1

1

6

4

13

4
5

1

8

11
1

1
6

1

1

3

3

1

2
26

3
14

1

7

1

2

1

4

3

2

37

4

1

3

33
1

29

28

1

1

1

1

109

13

4

4

9

4

4

8

2

1

5

4

4

7

5

2

1

1

4

4

40

40

8

4

4

3

3

18

1

1

3

4

1

8

1
3

2

57

3

3

3

42
16

14
1

3

6

3

1

2

10

12
2

1

9

9

206
9

37

1
3

2

31

2

3

26

26

3

3

3

3
1

1

1

12

7

7

3

3

3

1

1

1

1

3
52

12
1

4

1

1
4

1

1

1

2

3
32

8

1

4

3

1

16

4

5

3

2

25

5

3

3

2

6

6

12

12

2

2

6
3

1

1

1

7

5

5
2

1

2

1

1

1

1

2
55

2
5

3

3

6
1

2

1

1

3

9
4

4
5

1

6

1

1

4

4

27

27

189

189

189

69
189

6

5

108

1

9334
244

824
22

244
7

2

2

1

1

1

1

26
4

4

1

1

9

8

1

1

1

12

12

9

1

8

1

1

1

1

1

1

1

1

7
21

1

11

2

4

4

2

1

1

6

2

1

1

3

2

2

6

2

1

2

1

1

20
79

7

1

2

1

4

1

7

4

2

3

3
27

2

2

6

4

5

3

2

5

4
1

1

1

1

1

1

1

3

1

2

2

1

1

1

7

6

6

1

2

2

1

1

1

3
1

2

7

4

3

3

11

1

3

6

1

6

6

2

1

1

1

11

11

12

4

4

7

2
3

1

4

1

1

475
16

6

3

3

3

4
16

7

7

5

1

1

2

2

1
2

1

1

3
15

2

3

1

2

1

1

2

22

16

6

1

1

3

3

6

6

11
87

16
2

5

1

1

3

3

1

4

3

2

5

16

4

6

3

1

1

3

4

2

1

3

2

1

1

2

1

1

3

3

6

6

3

3

7

7

13
3

7
10

1

2

1

1

1

3

3

28

10
9

1

18

9

9

1
2

1

1

8

3

1

4

4

13
1

7

7

5

8
95

1

84

1

1

2

2

3

3

12
4

1

1

4
3

1

3

3

1

1

15

15

1

1

4
13

1

3

3

2

1

2

4
41

1

1

3

33

10
1

2

1
7

1

5

1

1

1

1

13

12
13

1

36

17

13

4

4

19

17

7

5

5

1

1

8

8

5

3

27

11

5

2

4

1

1

2

1

58
2542

2370
99

2

2

2

2

7

7

1

1

1

4

4
1

2

1

15
1

1

2

4

4

2

1

1

3

400
1916

3

3

3

6

28

124

1

9

3
1

2

7

5

8

1

1

10

1

2

3

18

2

3

1

6

22

113

10

2

5

4

2

1

7

5

16

4

10

7

7

3

25

13

1

6

2

4

9

6

6

4

1

14

3

3
2

1

3

25

4

2

8

16

3

4

4

1

13

1

5

3

4

7

18

8

2

4

17

5

1

2

12

3

10

11

1

9

1

1

3

120
738

13

2

4

3

4

6

1

2

1

3

6

5

4

14

1

6

2

2

6

1

1

1

11

4

1

2

1

1

3

1

2

13

1

1

4

2

1

5

3

1

2

1

6

1

1

9

2

1

3

2

1

2

1

1

1

23

2

2

3

4

4

6

1

3

17

4

6

3

1

9

2

4

2

1

1

1

8

10

3

1

3

2

4

6

3

3

3

15

2

6

1

21

1

3

3

5

1

12

14

3

172

4

2

2

3

5

6

5

1

5

2

3

2

3

18

2

1

1

14

1

2

8

3

1

7

4

2

2

3

1

1

3

3

23
99

3

10

2

1

1

1

2

18
37

1

3

1

1

1

1

3

1

1

1

2

1

1

1

3

1

1

1

1

1

3

1

6

1

1

3

3

5

5

1

1

7

7

3

2

1

1

2

2

12
127

3

1

2

6

3

1

2

1

1

2

3

65
11

1

2

2

5

1

3

1

3

1

1

1

2

3

4

1

1

4

1

1

1

3

2

3

2

1

4

3

2

2

1

1

4

8

2

2

8

2

4

2

5

5

5

10

6

4

27
6

3

3

7
12

1

1

1

1

1

5

1

13
1

10

1

1

9
101

2

2

8

1

2

1

3

1

19

1

1

10

2

1

4

1

1

1

1

16

15

1

20

19

9
1

2

1

1

1

1

1

1

2

1

7

1

1

1

6

1

1

1

1

2

4

1

1

2

6
1

3

1

1

6

3

2

1

2

2

1

1

1

1

1

1

1
617

12

12

7

4

4

1

3
8

3

2

596
30

2

2

1

1

1

1

1

1

1

1

4

1

3

1

1

1

1

1

4

3

3

1

2

2

1

1

1

1

15
63

2

1

1

3

1

14
5

2

1

1

1

4

1

3

1

3

1

1

1

2

1

2

2

1

1

1

1

3

1

1

1

6

2

2

4

2

1

1

1

8

8

1

6

1

2

2

1

1

1

1

1

1

3

3

1

1

2

1

1

1

1
5

4

6

6

6

8

2

2

3
1

2

1

2

2

2

1
15

3

10
1

9

1

1

1

1

3

1

1

1

4

4

1

2

1

166

166

1

1

13

2

1

1

1

8

4

2

1

1

1

1
15

9

9

1

4

2

2

1

1

1

1

1

1
6

1

4

3

1

2

1

1

1

4

1

2

2

1

6

3

1

1

1

2

1

1

1

4
8

4

7
1

2

2

4

2

2

1

2

2

1

1

1

1

3

3

4

4

1

1

4

3
4

1

1

1

1

1

1

5
1

4

3

1

11

11

11

4

4

4

2

2

2

2

2

3

1

1

1

7

5

1

1

3

3

3

3

12

1

2

7

1

3

1

2

1

1

5
1

2

2

2

1

1

36

4

5

3

24

6

2

2

4

1

1

6

6

6

7
2

3

3

2

2

1

1

8
1

6

1

1

2

1

1

1

1

1

1

1

4

3

1

1

1

3

3

3

6

6

3
10

1

4

2

1

1

1

1

1

1

1

1

1

8

2

2

6

2

2

1
2

1

1

2

2

2

2

98
3692

11

11

3

4

4

4

5

5

5

3
68

4
20

1

15
2

2

11

19

17

17

2

4

1

1

3

3

3

5

1
5

3

1

8

1
8

2

2

3

6

5

1

54
8

1

2

1

1

1

1

2

1

2

2

23

3

5

1

211

211
23

120

68

573
25

3
18

5

2

3

9

1

1
8

4

3

1

1

2

3

3

13

4

8

2

1

5

1

6

6

5

5

435
81

2

1

1

1

1

2

3

11

10
86

7

2

3

1

1

6

2

3

1

2

1

2

1

9

2

1

3

1

1

2

1

1

1

3

2

2

1

4

1

3

1

1

2

2

1

1

209

1

8

4

1

2

5

11

1

2

9
53

1

43
7

1

6

1

1

1

2

5

1

6

1

6

1

2

2

3
7

2
4

1

1

14
69

1

1

2

2

40
7

4

1

1
5

4

19

1

1

2

1
2

1

1

5
10

1

1

2

1

1

1

1

16

1

1

1

1

1

1

1

1

6

6

5

1

3

3

1

1

1

2

2

18

18

2

3

7

6

4

4

4

8

5
1

1

1

3

3

3

372
2

22

2

4

1

2

5

4

2

2

314
16

1

1

2

4

1

1

5

38
273

1

1

1

1

2

2

1

1

1

1

3

8

1

182

1

3

1

6

1

1

1

2

3

1

7

1

1

2

2

1

1

4

1

1

1

1

1

1

1

1

1

3

3

3

1

1

1

1

1

1

4

4

4

1

1

1

1

1

1

4

4

4

6

1

5

3

1

1

7

2

3

3

1

1

4

1

1

2

2

1

1

17
640

7

1

4

1

1

2

2

80
12

2
46

1

1

1

23

3

3

13

13

2

22
3

7

1

1

1

1

3

4

1

1

6

1
24

2

3

6

12

12

505
35

2

2

39
15

8

3

3

2

1

1

1

10

9

1

8

1

1

419
84

1

1

3

2

1

2

1

1

4

2

1

3

4

1

49
47

2

4

1

1

1

1

1

4

1

1

1

120
53

1

6

1

1

1

1

1

3

1

2

3

5

1

1

3

1

3

1

2

1

1

1

2

1

3

1

1

2

1

1

1

1

1

1

2

2

2

3

1

2

117

1

1

1

2

2

2

2

2

3

1

2

2

33

33

33

65
3

4
15

1

9
1

6

2

1

1

1

4

1

3

5

1

2

2

2

1

1

1

1

2
23

1

1

14
6

1

1

1

2

1

2

1

1

2

1

5

5

5

2

2

76

1
9

5

4

1

3

12
1

10
3

3

3

1

1

9

7

7

2

5
12

4

3

8

8

16

11

5

5

7
10

3

3

6

6

6

2

2

2

2

83
1108

9

5

5

4

18
79

2

3

1

1

2

2

5

4

10

3

5

3

1

14

3

2

4

1

2

2

2

3

1

2

2

906
267

5

1

7

409
145

3

1

7

5

4

1

10

1

1

10

2

1

6

2

3

17

1

1

4

1

1

1

1

22

1

7

4

1

5

3

1

1

4

1

2

2

2

7

1

18

5

4

3

4

5

3

1

16

5

1

1

1

4

1

4

22

14

1

1

2

36

3

3
1

2

11

5
6

1

2

20

13

1

10

19

1

7

10

7

5

5

1

33

8

5

10

1

29
8

3

6

6

3

3

158
2

3

3

9

5

4

59
44

15

20

2

3

14

14

1

27
1

4

18

16

2

4

15

2

13

13

11

11

2

10
1

6

1

1

2

6

1

1

1

1

1

1

2

2

1

1

6

3
6

2

1

1

1

1

2
80

6

2

4

2

2

4

4

51
2

1

5

2

17

9
15

2

1

1

1

1

6

1

2

11

4

1

3

2

1

3

5
1

2

1

1

1

1

5

5
1

1

1

3

2

1

158
3

4

3

1

5
151

41
2

3

4

5
26

2

1

3

15

1

1

1

3

3

2

1

13

2

1

1

9

1

1

4

3

31
8

4
14

1

7

1

1

1

1

1

3

1

2

6
57

3

2

1

7
44

2

3

1

1

1

1

1

4

1

1

1

1

1

16

2

1

1

1

9

9

2

2

3

2

1

1

4

4

7
103

53

4

1

1

3

2

1

1

2

1

1

1

2

1

11

1

1

3

1

3

1

1

23

4

4

4

4

1
19

1

1

1

1

1

14
1

13

1

1

1

1

1

1

1

1

1081

1

1

1

1

1

1072

2

2

1069

1069

1

1

8

1

7

2

2

2

2

6

6

6
3

3

15

3

3

3

1

2

12

1

2

2

2

7

2

2

6

6

6
3

2

2

1

18

1

1

1

1

11

4
8

1

3

3

1
3

1

1

1

6

6

6

6

516
16011

49

2

1

1

1

1

1

3

3

16
2

5

6

6

3

3

2

2

1

1

1

20
1

2

1

1

7

6

1

1

6

4

2

4

4

1

1

1

2

2

2

126

1
126

3
109

3

1

5

93

1

3

3

4

4

4

8

2

6

369

270
3

5

1

1

2

1

11

1

3

1

2

1

3

3

1

1

1

1

88

88

1

1

30
160

1

1

1

1

2

5

1

2
15

1

1

3

3

1

4

3

80

2

6

1

1
3

2

2

2

3

1

2

1

1

1

1

1

1

1

16

2
1

1

1
2

1

2
12

8
2

6

1

1

2

1

1

1

1

1

1

5

5

1

1

3

3

5

3

1

2

2

2

2

2

1
2

1

1
66

2

2

2

2

2

2

24
1

3

8

1

1

6

4

1

6

1

1

1
7

4

2

3

3

16

5

2

3

2

1

2

3

3

2

2

2

2

2

1

1

1

2

2

2

1

1

2

2

122

30

1

1

1

1

1

1

2

2

1

1

1

1

1

9

5

4

2

2

1
12

2

2

1

2

3

1

2

92

83
5

3
72

69

2

4

9

9

1

1

1

1

338

10

5
1

3

1

5

1
5

4

6

1

1

4

4

4

1

1

1

2

1

1

1

1

50

1
2

1

1

48

48

48

193

1

1

192

1

1

189

1

1

10

113

64

1

1

8

8

2

2

4

1

1

66

1

1

1

5

5

1

1

1

4

4

4

3

2

1

7
44

2

6
19

1

2

1

2

1

4

2

1

1

1

1

2

1

7

2

2

2

2

2

3

1

1

1

1

1

1

3

2

2

1

1

1

1

118
1

43

11
2

1

8

1

1

3

3

1

1

4

3

3

1

2

2

2

4

1

3

12

5

2
7

1

1

2

1

1

1

4

2

2

2

3
60

8

2

4

4

2

1

1

3

2

1

7

1

1

2

1

1

3

1

1

4

4

4

3

2

1

1

4

4

1

1

1

1

1

1

10

3

5

2

2

2

6

6

1

1

1

1

2

2

1

1

1

3

3

1

2

1

1

6

4

1

1

3

3

3

1

1

1

1

178
7266

480
2

3

3

447

446

1

1

2

2

4
21

8

5

1

2

1

1
4

1

1

2

1

1

11

6

6

1

1

4

451
40

15

6

6

9

19
3

4

1

11
1

3

7

204
309

2

4

7
8

1

7

3

8
10

1

1

1

1

20
65

1

2

1

2

1

3

1

14

1

2

2

2

1

5

6

1

1

1

3

3

2

2

49
7

2

4

2

3

2

2
16

1

2

5

6

9

4

1

6

6

10

10

258

7

2

1

1

3

7
1

1

1
4

1

2

1

4

4

4
240

2

233

1

165
4

2

2

3

3

15
5

3
1

2

2

5

2

2

14
102

5

6

2

2

1

1

64

8

2

2

1

9

3

3

6

22
1

17
5

11

1

3

1

6

6

6

5658
117

2

1

1

2

2

4
1

2

1

3
12

1

1

1
8

3

4

1495
11

1468
1374

94

1

2

2

2

8

2

1

759
5

730
1

29

679
12

76

1

368

213

8

1

16

1

4

1
24

5

1

3

1

5

1

7

4
2

2

1
16

12

3

9

3

7
37

3

3

23

1

1

1

5

5

36
5

30

1

1

1

1

1

1

5

4

1

1

1

1

9

1

8

1228

574

2

34

40

15

4

48

1

11

400

8

11

654
644

10

1

8

1

15
1909

1851

43

1

27

14

1

11
4

5

2

2

2

1

1

1

29

29

29
22

7

9

4

3

2

5

5
4

1

1
22

3
15

6

6

4

4

2

2

6

1
6

4

2

2

2

1

1

28

19

19
9

1

2

3

1

1

1

2

9

5

5

2

2

2

1

1

1

1

4245
12

1
2104

504
2082

14

6

3

2

2

4

7

5

7

7

3

1

4
118

8

8

2

6

7

85

5

4

1

1

9

5

1

2

1

1

1

1
13

12

12

1

3

3

2

2

4

33

3

18

2

10

3

1

2

1

5

3

6

2

2

4

3

79
373

3

1

7

2

1

4

5

1

3

3

3

1

6

1

1

4

1

3

8

10

2

1

4

2

2

3

4

1

5

4

1

1

4

1

3

2

6

2

1

2

1

2

3

1

7

1

2

2

5

1

1

2

1

2

1

7

1

3

1

3

3

5

9

2

2

2

1

5

2

1

14

1

1

8

4

9

4

2

3

2

1

4

3

1

1

3

4

2

1

4

1

6

1

7

1

1

4

2

1

4

76

1

2

4

3

12

2

6
450

1

2

243

7

2

129
128

1

1

59

1

1

28

7

2

2

13

9

4

1

1

6

1

3

5

1

5

281
15

19

3

1

2

14

2

8

190

190
66

124

124

42

1

5

1

1

3

2

2

16

8
14

5

1

2

2

3

3

3

3

3

3
2126

5
1

1

3

3

3

14
4

1

9

8

1

2092
78

1

13
5

1

1

1

1

1

1

1

1

1

2

1

1

1

1

7

369

2

1

2
1614

7

1605

6
1

5

2

2

1

1

3
919

916
3

1

1

1

435

6

6

1

9

409

10

9

1

10
472

1

2

1

71
5

66

66

66

89

2

1

219
1

36

180

2

1

2

1

12

1

2

3

1

3

2

1

17

10

2

1

18

3

4

2

2

3

1

104
4

61

1

2

5

3

1

1

2

1

13

1

1

1

1

1

1

1

1

1

1

1520
8

24
420

42

1

4

1

1
28

14

1

9

2

1

8

4

1

1

2

3

3

6

6

6

2

2

1

1

2

2

1

1

5

3

3

2

56

5

2

47
14

1

3

2

1

1

1

6

6

11

1

2

26
273

3

19

7

1

7

7

3

2

1

10

39
41

2

9

131
3

13

3

1

11

7

1

5

1

21

5

1

11

5

25

4

14

6

1

1

1086
16

3

1

1

1

86
6

2

4

1

6

1

3
51

39

2

1

1

2

3

1

1

3

1

1

2

1

1

3

1

1

1

562

561

1

270
82

42

1

1

2

1

1

76

16

1
3

2

1

1

4

2

8

24
28

4

2

11
50

1

26

25

1

1

8

1

1

1

1

1

1

2

3

20

20

1

1

39
5

2

2
7

1

1

2

1

1

1

6

5

5

5

2

28
1

1

1

1

1

7
21

1

1

4

4

4

1

1

7
1

3

3

1

1

1

3

3

6
3

1

1

1

13

13

1

1

12
10

1

1

1

153

153
2

2

1

1

1

1

1

3

3

130

3

7
127

120

120

2

2
1

1

1

1

5

5

1

1

4

4

31

5

2

2

2

2

2

1

1

1

1

4
2

2

5

1

1

1

1

3

1

1

1

1

4

4

3

1

2

1

13

1

1

1

7

5

4

1

2

3

3

3

1

1

1

1

21

1

1

1

1

1

1

1

1

1

1

9
1

1
2

1

2

2

4

4

4

1

1

1

8

4

1

1

1

2

1

1

1

1

1

1

1

1

1

66

3

1

2

2

2

2

3

3

1

1

2

42

1

1

2

2

2

3

2

2

1

6
1

5

5

1

1

1

1

6

4

2

2

9

7

1

1

1

10
1

1

8

8

3

2

1

16

16

16

6

1
4

3

2

2

2

1

1

1

1

1

302
40

1

1

1

1

127

1

1

4

56

4

57

1

4

1

1

1

5453
1

2

1

1

1

5436

6

6

6

1

1

5

5

5

3

3

3

2

2

2

1

1

3

3

3

3

1

1

2

2

5422
46

3

3

1

1

1

2

2

1

1

1130

74
1123

38

2

1

1

1

36

36

1

1

162

9

9

9

25

2

23

20

3

128

128

848

1

1

519

519

328

328

7

1

1

1

1

1

3

3
1

1

1

1

1

1

1

1

1

1

636
1

1
635

1

1

1

41
2

1

1

1
6

5

31
3

1

1

12
24

1

2

1

1

3
6

1

1

1

1

1

1

1
2

1

1

403
3

1

3

2

1

1
3

2

2
1

1

1

1

1

1

1

1

1

1

1

1

1

1

1

1

1

324

81

196

47

4

4

4

1

2

1

1

1

1
39

1

16

2

1

1

17

1

2

1

2

1

5

3

1

1

1

1

1

1

1

1

1

1

1

1

1

9

8

8

1

22

22

167

159

159

5

5

2

1

1

1

1623

55
1623

2

1

1

4

1

1

1

2

2

1

1

1

58

50

50

2
5

2

1

1

1

1

1

1

1

1

1

1

1

4
267

1

1

1

1

4
36

3

1

5

2

2

3

4
11

5

1

1

1

1

1

2

2

118

118

118

1

1

1

89

89

1

1

1

8

1

5

1

1

1

3

1
3

2

1
5

1

1

1

1

1

1

1

1

1

2

2

2

14
1232

2

2

1
3

1

1

199
1199

5

1

1

6

294

1

1

1

2

2

3

3

2

6

3

1

1

1

1

3

4

2

1

4

6

1

3
5

2

2

3

10

1

1

1

3

7

18

593
61

2

6

5

1

3

3

1

3

3

20

1

1

26

26

282

2

1

3

33

1

5

3

4

3

8

6

56

9

9

4

2

1
9

1

3

1

1

1

1

2

1

3

1

1

1

1

1

1

1

1

25
5

1

1

1

3

8

1

1

1

1

1

1

1881

1881
1

5
1868

1

1

18
1

2

5

1

1

1

2

1
4

1

1

1

1

1

1

2
17

2

11

2

1

1

2

1

2

1

1

1

1

1

1

135
2

1

131

1

1

1

1689

1689

1
12

1

8

1

1

78

78

4
78

1

1

1

1

13

1

1

5

7

7

7

7

2
1

1

1
4

1

1

1

1

1

2

2

3

2

1

1

1

1

1

1

1

1

1

27
8

2

2

1

1

1

4

3

2

1

2

1

1

1

1

1

1

1

1

8

7

1

2

2

2

2

2

2

2

1

1

9

3

3

1

1

5

5

1

1

4

2

2

1

1

1

1

1

1

1

2

1

1

1

1

1

1

1

1

1

1

1605896
