# Supplementary material for: Metagenomics of African Empogona and Tricalysia (Rubiaceae) reveals the presence of leaf endophytes
Source: PeerJ. 2023 Aug 4;11:e15778. doi: 10.7717/peerj.15778 (PMC10405798; doi:10.7717/peerj.15778)
Supplement: Data S1 [file peerj-11-15778-s003.zip › Tricalysia lasiodelphys.html]

Javascript must be enabled to view this page.

members
magnitude
magnitudeUnassigned

kaiju.out

2000000

1532363

467637
23

467249
3659

281
9527

26

2
1

1

1

1

1

19

19

1

1

18

18

18

5

5

5

5

5

3
2

1

2

19

19

19

9

9

9

9

10

10

10

10

10

590
3

1
129

46

16

11

11

11

4

4

4

1

1

2
6

1
4

1

1

1

5

5

5

5

5

30

4

3

3

1

2

2

1

1

1

24

24

2
24

3

3

3

19

19

19

2

2

2

3

3

3

3

3
1

1

1

1

4

1

1

1

3

3

3

3

5

4

4

4

4

1

1

1

1

70

4

4

4

4

4

4

65

8

3

3

2
3

1

5

3

3

3

3
1

2

2

2

2

57

57

1
57

2

2

2

54

54

54

54

1

1

1

17

1

1

1

1

1

1

6

6

6

6

6

6

1

1

1

1

1

1

9

9
8

1

1

441
2

37

9

8

5
8

1

1

1

1

1

1

1

1

1

1

1
14

1

1

1

1

1

1

8

8

7

1

1

1

1

3

3

3

3

3

3

3

1

1

1

1

1

4

2

2

2

1
2

1

1

1

1

1

1

1

1

1

1

1

1

13

13

8

7

7
4

3

3

1

1

1

5

5

5

5

1

1

1

1

1

4

4

4

4

11

4

2

2

2

2

2

2

2

2

7

7

7

7

7

6
387

328

328
5

5
22

2

5

2

1
2

1

2

1

1

3

1

1

298

298

1

1

1

1

1

33

5
33

1

1

2

2
23

12

1

6

2

2

2

9

9

2
9

4

4

3

11

11

4

4

7
1

4

1

1

1161

1161
84

10

10

10

10

10

1
100

3

3

3

3

3

60
5

3

3

3

3

52

5

5

5

47

47
27

1

8

1

10

2
36

1
25

5

5

5

1

1

4

4

3

3

1

1

1

15

1

1

1

1

1

1

13

13

3

10

10

1

1

1

1

1

1

1

1

1

7

7

1
2

1

1

5
1

3

1

35

35

35

35

9

4

4

4

4

4

5

2
5

3

3

1

33

33

33

33

15

18

11

11

11

1

1

10

10

10

164

164
6

113

1

1

3
112

23

86

86

2
45

16

16

27
7

9

9

9

11

675
17

298
16

44

44

16
1

11

4

28

28

28

1
63

54

49

49

49

5

5

1

1

1

7

4

4

3

2

1

165

165
4

30

30

7
109

12

12

90
1

13

17

1

58

58

2

2

4

4

1

1

15

15

15

1

1

1

1

9

9
1

6

6

2

2

2

360
6

13
338

62
7

7

7

7

5

5

5

20

20

20

14

6

23
3

8

8

1

1

11

11

25
263

3

3

3

3
27

17

17

7

7

7

20

20
1

19

7

7

7

18

18

18

163

161
5

16

12

12

128

2

2

1

1

1

1

1

15

11

11

11

4

1

3

1

1

38

38

38

35

35

3

1

2

9

3

3

3

3

3

1

1

1

5

5

5

5

56

1

1

1

1

5

5

49

47

47

47
1

46

2

2

1

1

1

1

1

1

1

18

9

4

1
4

1

1

1

1

2

2

2

2

2

2

5

5

5

1

1

1

2
4

1

1

1

1

1

1

3
1

2

1

1

1

1

1

1

6

6

6

6

6
2

1

3

4
76

1

1

1

1

1

51
3

3

3

3

3

3

3

1

1

44
4

2

2

2

2

32
3

1

27

2

2

2

16
15

1

9

9

6

6

3

1

1

1

1

1

1

1

5

5

5

5

20

12

3
12

3

3

3

3

3

3

3

4

4

4

4

4

4

4

4

54

7

7

7

7

7

7

7

47

2

2

2

2

1

1

1

1

1

36

35

18

1

1

9

9

9

8
1

1

1

6

4

2

1
17

10

8

8

8

2

2

2

6

6

6

1

1

1

1

1

1

9

9

9

9

9

1
7232

7219
178

21
1060

22

9

2

2

2

2

2

3

3

3

1

1

1

2

2

4

1
4

3

3

2

1

8

2

2

2

2

2

2

6

6

6

6

6

6

5

5

5

4

2

2

2

2

1

1

1

66

66
1

30

30

30
22

3

1

2

2

5

1

4

1
35

7

7

2

2

5

5

3

3

1
3

1

1

1

1

1

1

9

6

6

6

3

2
3

1

1

12

12

12

5

7

2

2

2

6

6

2

2

2

4

4

33

12

5

4

1

1

1

1

1

1

1

1

5

5

1

1

20

20

20

19
20

1

904
2

60

60

60

19
60

1
2

1

1
14

9

3

1

1

1

1

5

1

3

1

2

1

2

2

14

13

1

1

1

2

2

273

6

6

6

6

4

2

10

10

6

1

1

1

5

5

4

4

4

257

7
257

11

1

1

10

10

2

2

2

2

14

9
14

4

4

1

222
5

109

1

1

9

2

5

2

1

1

98

95
1

81

13

3

107

107
32

66

6

3

1

1

1

1

1

1

1

569

1
569

54

53

53

53

1

1

514

514
1

100

100

412
4

148

15

15

113
245

52

80

1

1

8

8

8

8

4

4

4

4

4

4

4

4

12

12

5969
54

10
5175

15

2

2

2

2

6

6

6

6

4

2
4

1

1

1

1

1

1

1

3

3

3

3

3

3

5150
18

5036
47

61

61
3

7

7

7

7

4
13

8
3

1

1

1

2

1

1

1

12
2

1

1

2
3

1

6

6

10
3

3

3

3

2

2

1

1

1

1

1

5

5

5

11

11

11

11

40

40

9
40

8

1

7

5

2

3

3

10

1

8

1

1

6

1

1

5

2

2

2

259
4888

92

3
92

3

1

1

1

1

1

1

2

2

2

47

47

47

47

47

39

1

1

1

1

38

38

1

1

1

33

1

1

26
32

1

5

2

1

1

1

1

2

1
2

1

2253

5

5
1

1

1

1

2

2

1

1

1

1

1

143
2248

28
992

33

11

11

11

11

12
22

7

7

3

3

29

6
29

11

11

11

12

12

70
640

14

9

9

9

5

5

2

2

2

11

11

11

9

9

9

9

10

10

10

10

105
5

13

13

13

1
45

17

17

15

15

15

1
8

4

3

4

4

4

11

11

11

11

1
21

4

4

4

8

8

8

8

8

10

10

10

2
35

17

17

16

16

16

10

10

10

10

16

16

16

16

11

11

11

14

14

14

14

18

18

18

22
1

11

11

10

10

268
25

3
85

48

48

48

34

34

34

26

13

13

13

13
1

12

6
74

3

3

5

5

5

18
5

4
2

2

2

3

3

6

19
9

10
5

2

2
1

1

1

3

3

4

4

16
9

4

4

2

1

29
2

4

4

9

3

6

4

4

1
10

2

1

1

6

29

8

8

6

6

6

11

11

4

4

4

4

4

11

11

11

11

14

14

14

14

4
87

27

27

27

38

38
18

7

8

5

18

18

18

175

27

27

27

27

148

148

148

11
403

18

18

18

18

5

5

5

26

26

26

26

25

25

32

32

32

28

28

28

28

14

14

14

14

14

30

30

18

18

18

18

18

3
135

24

24

24

24

28
85

11
2

4

5

8
1

7

14

14

14

14

14

10

10

10

23

23

23

23

21

21

21

21

15

15

15

15

25

25

25

25

21
710

70
4

13

13

13

13

53
6

10

10

3
37

13

14

7

38

23

23

23

23

15

15

15

15

85
2

57

57
16

18

6

6

12

12

5

5

26

26

26

28

28

16
28

5

5

7

16
468

16
180

15

15
1

14

1

1

17

17

28
10

1

4

4

4

9

9

9

9

27

27
1

26

27
19

4

4

24
40

4

3

6

6

3

10

10

10

10

7
262

66

66

9
43

16

18

37

37

32
37

5

23

23

23

3
67

23

40
41

1

19

19

1171
19

585
9

34

34

34

27
34

7

22
521

60
17

17

12

5

9

45

45

45

45

4

2

2

2

2

390
88

40

7

33

9
33

2

2

7

7

8

8

3

4
3

1

21
152

15

32

16

6

18

18

27

27

17

3
22

6

2

2

2

9

25

25

25

30
5

14

14

11

11

21

21

21

21

2

2

2

2

2

565
11

17
437

1
47

1
33

8

8

9

2

2

5
3

1

1

2

4
15

6

5

13

13

13

14

14

10
14

4

20
350

6

6

17
108

2

1

1

1

2

5

3

35

4

1

15

2

3

5

2

1

1

1

1

1

6

3

3

3

86
213

13

4

4

4

3

3

2

2

1

2

3

2

2

1

1

1

1

1

1

1

25
4

3

3

8

8

1

4

2

3

1

1

2

2

4

4

5

1

1

1

1

8

5

5

1

2

1

1

1

1

1

2

2

4

4

4

6

1

5

1

7

7

1

4

4

1

1

2

1

1

2

2

2

5

2

3

3

3

9

9

9

8
117

13

13

13

13

19

3
6

3

13
10

3
1

1

1

44
7

1

1

1

9
1

2

1

1

2

1

1

3

12

12

12

3

2

2

1

1

6

6

6

6

2
6

2

1

1

6

6

6

6

9
27

2

2

4

4

4

8
7

1

1

4

4

4

8
1086

15
398

2
69

18

14

14

14

4

4

4

31

31
12

1

17
11

1

1

1

1

1

1

1

18
1

4

4

13

15
177

20

6
12

2

4

8

2

6

6
1

5

15
3

9

7

7

2

3

3

18

10

10

3

3

5

5

2

8

8

16

16

16

16

6

6

6

5
10

5

13

13

10
13

1

1

1

14

3
14

1

1

1

2

5

1

7

7

7

10

5

2
5

2

1

5

5

2

2

2

9
29

9

1

4

4

1
4

3

2

1

1

1
5

2

2

2

2

2

2

2

2

137

137
5

8

8
1

7

1
116

2

113

7

4

1

2

1

1

680
24

136

5
136

9

9

9

2
25

1
8

4

4

3

15

14
5

6

2

1

1

6

6

6

6

37

13
20

5
1

1

1

1

1

2

17

17

12
1

3

3

1
8

3

1

3

9

9

9

33
7

6

6

6

20
13

4

4

3

185
5

13

1
5

2

2

2

2

2

2

8

8
5

1

2

2

3
54

6
2

4

4
3

1

1
41

15

9

9

9

6

6

6
7

1

1

5

4
5

1

13

13

13

4

4

4

23

23

23
1

9

4

4

9

9

2
71

9

9
1

6

2

5

5

5

5

5

2
53

48

2

4

42

3

3

2

2

2

4

4

4

4

4

15

4
15

7
3

4

4

4

4

4

5
335

2
82

1
15

8
6

2

6

6

6

65

13
65

1

2

1

1

23
10

2

1

6
7

1

1

1

1

1
5

2

1

1

1

5
1

2

2

2

4
7

1

2

1
4

2
1

1

1

1

4

4

10

2
4

1

1

1

6
1

3

3

2

2

238
13

31
4

4

4

3
6

1

1

2

3

3

5

2
5

1

2

1

1

1

4

4

4

4

4

4

4

4
3

1

6
34

9

1

1

3

3

2

3

3

3

3

4

4

4

1
9

3

2

2

3

2

2

2

4

4

4

4

21
4

4

4

1
12

1

1

6

6

4
3

1

1

1

31

3

3

12
28

1

3

3

2

2

2

2

2

1

1

1

1

1

3

98
1

1

1

1

12
91

19
2

1

3

4

1

1

3

1

2

1

3

3

1

1

3

3

1

1

7

1

2

1

3

5
13

2

6

3

3

3

6

2

2

2

4

4

1

1

4

3

1

13
5

2

1

1

4

4

1

1

1

3

3

1

1

27

27

27

27

27

27

96

96

96
22

13

1
2

1

1

1

1

1

1

1

1

1

1

1

1

1

1

4

4

4

3

1

2

2

2

1
6

1

1

4
1

3
1

2

11
2

2

1

1

1

1

2

2

1

1

1

1

1

1

1

1

1

1

3

1
3

2

6

1

1

4

4

1

1

2

2

2

2

2

3

2

2

1

9

2

1

1

3

3

4

4

4

11

11

11

11

10

5

5

1

1

4

1

4
1

2

1

1

3
740

170
3

8

8

8

1
8

1

1

3

1

2

2

33

3

3

3

3

30

1

1

1

29
5

1
2

1

1

8

8

8

4

3

1

6

6

6

4

4

126

4

4

2

2

2

2

10

10

10
1

1

8

18

18

13
18

5

3

3

3

3

2
90

67

67

66

1

1

21

21

21

1

1

1

1

1

4
459

113

3

3

3

3

3

3

3

3

1
102

7

4
7

1

2

2

7

7

7

83
1

10
2

2

2

5

1

2
72

2

2

1

1

1

5
4

1

62

62

3

3

3

1

1

1

5

5

1

1

2

2

2

2

2

286
5

164
1

1
61

2

2

2

2

13

11

11

11

2

2

1

1

42

1

1

41

41
24

16

16

1

1

3

3

3

3

1

1

1

1

1
10

1

1

1

1
8

1

1

4

4

1

1

1

1

1

2

2
1

1

1

1

3

1

1

1

1

2

2

2

2

7

2

2

2

2

2

2

3

3

3

2

1

1

1

1

2

2

2

1

1

1

1

74

28

3

3

4
1

2

2

1

2

2

19
5

12

12

2

2

2

2

1

1

1

1

1

1

2

1

1

1

34

34

34

34

1

1

1

1

2

2

2

2

2

2

2

2

2

2

2

2

3
117

3

3

3

3

3

5

5

5

5

5

4
88

8

5

3
5

2

1

1

1

2

1
2

1

2

2

2

5

5

1

1

3

3

1
3

1

1

1

1

8

8
1

2

3

2

2

1

1

5

4

2

2

1

1

1

1

4

1

1

1

2

2

1

1

1

7

6

5
6

1

1

1

2

1

1

1

1

1

23

23

23

23

1

1

1

8

6

2

4

1

1

1

1

6

2

2

2

1

1

1

2

2

2

1

1

1

1

1

17

5

1

1

1

1

4

4

2

2

2

2

4

1

1

3

3
1

1

1

4

4
1

1

1

1

2

2

2

3

3

1

1

1

2

2

2

1

1

1

56

56

56

56
1

55

55

6

6

6

6

6

1

5

102

17

6

6
1

5
2

2

1

10

10

10
1

3

1

1

5

1

1

1

85

59
85

4

4

3

3

1

21

2

2

8
19

3
2

1

1

2

2

3

3

3

3

1

1

1

1

1

12

12

12

10

10

2

2

2

5

5

5

5

5

1
184

105

75

75

75

4

4

1

1

1

1

1

1

1

1

1

1

1

1

1

1

1

24

2

2

2

2

1

1

1

1

1

1

1

1

1

1

20

20

1

1

1

1

1

5

2

1

1

1

1

1

3

3

4

4

56

3

1

2

1

1

1

1

1

1

1

1

1

51
1

10

7

5

5

5

2

2

1

1

1

3

1

1

2

1

1

1

1

1

40

26

7

7

7

3

1

1

2

1

1

16

1

1

1

1

2

2

11

1

10

10

8

8

1

1

1

7

7

6

4

4

1

3

3

2

1

1

1

3

2

2

1

1

1

1

1

1

3

3

7

1

1

6

6

453879
10500

193

1

1

192

2

2

2

1
2

1

1

2

1
2

1

1

1

1

9

1

8

1

4

1

1

1

1
6

1
5

1

1

1

1

17

17
1

3

13

5
4

1

3

3

1

1

2

1

1

7

7

6

1

66

66
8

58

54

13

6

2

1

1

1

1

1

1

1

1

1

1

41
10

3
5

1

1

2
1

1

1

10

10

2
1

1

1

1

1

1

1

2

2

2

1

1

1

1

2
1

1

1
2

1

1

1

4

4

1

1

2

2

1

1

2

1

1

2

1

1

2

2

123

14

1

1

1

104

1

1

3

3

3

2

2
1

1

1

1

2

1

1

1

1

1

1

1

25

7

5
1

1

2

1

2

2

2

1

1

1

1

18

18
7

1

10

3

3

3

3

1

1

1

1

1

1

1

5

5

1

1

1

1

4

4

1

1

3

3

29

5

3

1

1

24

1

1

1

1

1

6

6

1

1

3
5

1

1

17

15

4

4

7

1

1

1

5

5

1

4

4

2

2

6175
1

6051
14

12

12

12
1

10

1

1

1

6

1

5

3
2

1

1

1

1

1

11

3

1

1

1

8

5

1

1

1

4

1

1

2

1
3

1

1

1

1

10

10

10

10

8

8

1

1

1

1

1

183
6003

11

11

2

2

2

6

1
6

1

1

3

3

1

1

2

1

1

3
1499

1496
25

1

1

1

1

1

1

1

1

1

11

9

1

1

3
136

11
2

5

3

1

1

1

1

1

1

2

2

1

1

24
98

2

4

1

2

1

2

2

1

1

2

43
8

2

1

1

3

1

3

1

14

1

1

1

1

1

1

1

1

1

1

1

1

2

1

2

1

2

2

7

1

4

1

1

14
2

3

2

2

1

1

1

1

2

1

1

1

1

1

1

1

1

1

1

3

3

1

2

1

1

1

1

1
3

1
2

1

80

4

4

1

1

1

1

1

1

1

12
1

1

2

2

1

1

1

1

3

1

6

1

1

2

1

1

1

52

50

2

1

1

2

1

1

74
1212

79

78

78

1

13
52

1

6

5

10

1
10

1

1

7

7

3
15

1

2
7

1

3

1

2

2

7
3

1

3

4

4

1

1

1

1

6
20

6

8

8
1

6

1

2

2

1

1

21

1

1

17

1

1

6
26

1

1

1

2

1

1

1

1

1

5

1

3

1

1

4

4

3
13

10

857
228

22

10

22

19

25

16

11

18

17

17

14

7

13

14

3

39

25

319
48

15

14

12

23

100

16

2

15

47

27

20

15

4

4

1

1

7

7

11

1

4
10

2

3

1

3

1

2

1

1

14

1

1

4

3

3

1

9
1

2

4

2

5

2

2

1

1

1

1

1

1

1

110
4

6

3

1

1

1

1

1

1

1

1

72

1

1

2

6

3

2

1

1

1421

1414
2

7
1323

92
16

1

2

3

1

1

1

2

5

2

1

1

1

1
42

2

3

3

3

2

1

2

3

1

1

1

3

2

1

1

2

2

1

3

2

1

1

1

2

2

3

1

1

3

7
1

1

4

1

2

1

1

1
4

1

2

9
105

2

1

1

1

1

1

1

8

3

1

61

6

1

5

1

3

17

3

1

17

1

1

1

1

1

1

1

1

1

2

3

2

1

1

2

1

1

5

5

978

978

2

2

5

5

4

1

116
6

1

9
1

3

2

1

1

1

2

2

1

2

91

1

1

3

3

3
86

1

1

54

1

2

14

3

7

2
7

4

1

1280

1

1

1247
77

29

1

1

26

26

1

1

1

1

173
1

18

18

18

36

32

4

28

3

1

118

118

968

376

376

1

1

589

589

2

1

1

7
32

2

2

2

2

2

7

4
7

1

1

1

1

1

1

1

1

6

6
2

1

1

2

1

1

1

1

1

1

1

1

1

1
5

4
2

2

1

1

453

1

1

452
1

39
2

18
2

2

1

1

1

3
13

1
4

1

1

1

1

1

1

1

2

4

4

1

1

1

1

1

2

11

11

37

1

1

1

1

1

34

34

11
370

1

1

1

3

3

6
96

1

1

1

1

1

15

1

1

1

2

1

1

1

1

59
1

1

45

1

1

7

2

1

1

1

1

1

1

1

1

1

1

3

2

1

1

1

1

1

1

1

1

1

1

1

1

1

1

4

4

1

1

1

1

10

4

3

1

1

1

1

1

1

1

4

1

1

1

1

1

1

1

1

1

1

1

1

14

13

13

1

3

3

1

1

1
3

1

1

2

2

2

1

1

6
1

1

1

2

1

1

1

2

2

181

71

85

25

1

1

4

1

1

1

1

1

1

1

1

1

5

1

3

1

4

4

1

1

1

1

2

2

1042

1042

1042
87

13

13

32
4

2

4

8

3

3

8

3

2
17

8

4

4

6

1

4
140

8

27

63

26

26

12

127
1

121

1

2

2

1

1

45
4

16

16

7

7

10

8

4
77

30

43

23

20

7

7

6

1

7

7

81
18

15
1

1

1

1

2

1

1

1

1

3

1

1

2

1

2

1

2

2

1

3

6

1

2

1

2

1

2

1

2

2

4

1

2

3

1

1

2

12
1

4

3

3

4

36
3

5

6

1

10

11

4

4

3

4

4

4

102

102

14

14

19

3

2

4

4

6

3

1

2

22

22

22

12

2

1

1

1

5

3

4

1

3

3

3

2

2

2

2

27
2

4

10

2

2

6

6

4

1

1

1

11

11

31
4

9

1

6

2

4

4

7

11

6

5

1
4

3

69

69

4

4

8

8

7

7

7

4

1

1

1

1

1

1

1

1

1

2

2

2

2

2

4

2

1

1

1

3

3

1

2

1

1

1

2

1
2

1

2

2

103
2

1

1

60

60

11

11

49

17
1

4

4

8

1

6

1

29

29

3

3

6
38

1

2

1

4

1

1

22

2

2

2

2

2

4

4

3

1

4

3

3

1

1

1

1

14

14
1

1

1

1

1

1

1

3

3

1

432
33413

2

2

78

1

1

77

13

13

11
2

1

2
1

1

2

4

1

1

1

50

50

1

1

12
49

1

1

4

1

2

5

1

1

1

1

2

1

2

1

1

1

4

3

1

1

1

1

1

3

14

14

2

2

12

7

7

5

49
779

11

1

10

10

8

8

2

2

6

6

2

2

1
6

1

1

4

4

1

1

3

3

3
261

14

14

14

14

14

8

8

8
2

3

3

3

171

74
2

17

17
2

7
3

3

1

1

1

6

55

23
14

5
1

1

3

4

7
21

3

11

11
6

3

2

97

3
97

65

65

29
10

7

12

12

48

48

17

17

9

4

2

1

1

5

5

49

14

14

14
4

10

2

2

33

6

1
6

2

2

3

5

5

5

1

1

11

11
1

5

3

2

4

1

10

4

6

20

11

7

3

1

2

1

1

2

2

1

1

4

4

9

5

1

1

1

1

1

1

1

81

2

2

2

10

10

10

10

5

5

5

5

69

2

2

2

64

64

3

3

17

16

15

1

1

1

1

1
265

2

1

1

1

1

1

1

1

1

54

1

1

192

1

1

1

3

5

5

5

5

5

5

1

1

1

1

1

652

46
637

1
141

42

3

3

13

3

2

1

1
3

2

2

1

1

1

1

1

2

2

2

2

1

1

1

1

1

1

16

16
1

15

1

1

1

4
8

1

1

1

1

1

1

2

2

98
12

5

5

5

5

1

1

1

28

8

5

3

2

3

16
7

9

1

1

3

3

1

1

1

3

3

3

3

4

1

3

2
33

8

7

4

2

1

1

1

6

6

6

1

1

1

2

2
1

1

1
13

2

2

8

1
2

1

1

12

7

7

7

3

1

2
1

1

2

2

2

4

4

3

2

1

1

1

1

6

6

6

6
1

1

4

268
34

1
12

11

2

4

4

1

2

1

1

2
23

2

2
1

1

10

8

8

1
2

1

1

1

1

1

1

1

6

6

11

11
1

1

1

2
7

1

1

1

2

1

2

2

2

2

7
104

2

2

1

1

1

1

1

90
19

2
1

1

1

1

3

1

2

3

1

7

7

1

5
50

1

2

1

1

1

8

1

1

1

1

5

4

12

1

2

3

2

1

1

1

1

3

3

3

57
2

4

4

21

2

15

1

3

11

4

30
18

1

7

7

4

4

4

4

18
5

1

1

7

7

7

5

2
1

1

3

1

1

1

6

6

6

6

6

42
2

1

27

1

2

6

2

1

1

1

1

1

2

2

2

2

14
121

1

1

2

2

2

4

4

1

3

3

2

2

2

2

1

1

1

4

1
4

2

2

1

13

12

11

1

1

9

1

1

1

1

9

2

2

7

7

7

35

1

1

30

23
2

1

2

1

1

2

4

1

2

1

6

2

1

1

3

1

1

1

3

3
2

1

1
36

5

5

30
2

1

1

1

1

25
3

10

3

2

1

1

1

1

1

2

1

1

9

8

7

1

1

1

1

5

5

5

51

1

1

1

1

1

1

49

1

1

1

1

4

4

4

2

2

1

1

1

1

6

6

6

1

1

5

5

38

38

38

38

3249
15

534

1

1

184
21

1

1

1

5

5

1

4

4

2

1

1

1

1

1

29
7

2

1

1

1

4
5

1

1

1

1

1

2

1

1

1

8
1

6

6

1

3

1

1

1

2
5

2

1

1

1

1

1

1

1

1

24

2

2

18

18

2

2

2

2

4

1

3

39

1

1

7
34

8

5

1

1

1

1

5

1

1

1

1

1

1

1

6

1

4

1

1

1

1

3

3

30

1

1

1

3

2

1

1

1

14
1

11

2

2

1

1

1

1

1

1

1

1

1

1

1

1

2

2

2

2

1

1

1

1

1

1

1

11

4

4

2

1

1

1

1

1

2

1
2

1

1

3

3

3

5

2

2

2

1

1

1

1

1

1

7

2

1

1

1

1

4

3

1

1

344

2

2

1

1

1

1

342

342

342

1

1

1

1

1

3

3

3

3

3

45

45

1

1

1

1

44

40

1

1

39

1

1

1

1

2

1

1

2

2

2

2

2

47

1

1

46

44

4

4

3

3

37

37

1

1

1

1

1

1

7
2545

443
1

2

1

1

1

1

22

21
1

1

1

1

6

8

1

1

1

1

2

2

2

2

1

57

57

55

2

2

257

251
1

1

23

218

1

4

3

1

1

2

6

6

104
4

2

1

1

1

1

6

6

1

1

16
10

1

1

1

2

2

1

12

12

2

1

1

59

28

31

88
2095

14

1
13

1

7

1

1

1

1

1

1

4

2

1
2

1

2

2

2

2

117

117

117

695
2

59

1

58

4
512

448

5

1

21

1

1

30

2

118

116

1

1

4

105

1

1

1

2

1

1

1

1

1

5

1

1

1

1

1

11
93

3

1

4

1

6

44

7

2

1

2

1

1

1

1

1

12

1

1

6

1

2

1

1

1

1

2

1

3

1

1

1

1

1

2

6

1

1

1

1

1

1

1

1

2

1

1

30

1

1

2

2

18

18

2

2

1

1

2

2

2

1

1

1

1

1

1

1

1

14

2

2

1

1

8

8

1

1

1

1

1

1

1

1

1

1021
19

96

1

1

1

94

1

1

3

2

1

2

1

1

9
3

1

1

5

2

2

2

2

1

1

1

1

3

3

1

1

4
2

1

1

11
833

2

1

1

1

1

1

9
75

66

41

25

1

1

1

5
361

3

4

1

140

4

1

1

1

2

122

26

1

50

1

8
172

5

46

3

110

11

1

1

1

1

188

1

1

1

1

1

10
1

6

2

2

1

1
18

1

1

10

6

1

1

1

2

2

1

1

1

1

1

1

3

3

1

1

1

1

1

1

2
1

1

7

6

1

1

1

3

1

1

1

5
48

3

3

3

3

12

12

1

1

3

1

2

1

1

3
2

1

1

5

5

5

28

4
11

1

1

2
1

1

1

1

1

2

1

1

1

1

17

3
1

1

1

1

14

14

11

1

1

4

1

2

1

1

2

1

1

2
30

4

4

4

4

4

4

4

4
8

2

2

11

3

3

3

3

8

8

8

8

1

1

1

1

1

14

2

2

2

12

245
28120

85

2
81

6

6

6

13
31

18

8

8

4

1

5

6

6

6

16

11

5

16

1

1

14

14

1

4

4

1
4

3

21
1

7

7

7

1

6

6

1

1

1

1

9

9

2
9

1

1

4

2

3

3

3

3

19
1

1

1

17

5

5

5

12

7

2

2

5

5

5

5

155

13

1

1

12

12

5

5

7

142

53
2

26

26

25
2

1

5

3

6

2

4

6

2

89

89

89

1518

28
1478

303

20
303

63
84

13

8

155
153

2

44

426
1

47

92

286

92

6
92

26

26

34

8
581

450
42

137

189
1

91

97

82

123

123

48

48

40

40

40

40

1534
26019

3166

3166
45

32

2

1

3

4

1

12

4

4

4

2

3

1

3

7
2

3

2

5

5

496
3044

3

1

2

1

2

1

1

78

2

2

1

2

3

1

1

2

1

1

1

1

1

1

139

1

1

1

1

1

1

57

1

1

80

2

2

1

1

1

1

1

1

867

1

2

2

4

2

1

2

5

3

7

2

1

2

11

1

1

1

1

2

4

1

585
123

1

1

2

1

1

2

1

1

1

2

2

1

1

1

1

76

2

1

1

1

1

1

8

1

1

2

2

1

1

2

1

1

1

1

2

1

1

1

2

1

4

2

4

1

1

2

3

1

1

1

1

1

1

16

1

1

7

2

1

2

1

1

1

11

2

1

3

1

1

1

1

2

2

1

1

1

87

1

2

5

1

1

1

1

2

2

1

1

2

1

1

2

1

1

2

1

4

1

1

1

1

2

1

2

1

1

2

4

1

2

1

2

1

5

2

1

2

1

1

1

3

1

1

1

1

2

1

2

3

1

1

1

1

2

2

1

1

1

1

1

1

1

1

1

1

1

1

1

3

2

1

1

1

1

1

2

1

1

1

1

3

1

1

2

1

1

2

1

2

1

7

3

1

1

1

2

1

1

1

1

1

1

1

2

2

2

1

1

1

1

1

1

1

1

1

1

2

1

1

2

1

1

2

1

1

1

1

1

8

1

2
1

1

1

2

1

1

1

2

2

2

1

1

39

1

1

2

5

1

1

1

2

82

2

2

1

1

1

2

91

36

10

33

1

2

5

2

2

1

3

1

1

2

1

2

1

2

1

25

1

1

2

2

1

28

1

1

1

1

1

1

2

3

1

2

1

1

2

1

2

1

2

105

4

1

1

1

1

1

1

1

1

3

1

1

1

1

1

1

1

7

4

1

6

2

3

1

1
3

2

1

2

8

1

5

5

28
3

2

1

1

1

2

1

1

1
11

3

2

1

1

1

2

4

1

114
779

5

5

5

298
3

12

12

3

3

31
265

2

1

5

3

1

1

9

2

1

1

2

3

2

1

130

2

1

5

1

3

4

2

2

2

4
43

1

1

2

1

4

3

1

1

4

3

1

2

1

2

3

1

4

2

1

1

1

2

2

4

3

1

11
6

1

2

2

13

13

13

3
98

1
4

1

1

1

3

3

4

4

4

4

59
17

1

1

1

1

1

3

4

1

2

1

3

1

1

1

1

2

2

3

2

13

2

2

2

2

6

6

9
1

1

4

2

2

3

2

2

23
251

5

4
5

1

10
16

1

2

2

1

2

77
19

1

1

2

1

2

3

1

1

3

2

3

1

3

1

1

1

14

1

4

1

1

1

2

1

3

2

4

1

1

1

2

4

2

15
5

3

1

3

3

1

1

5
2

1

2

25
4

1

2

2

4

3

7

4

4

4

2
5

1

1

1

3

3

31
10

4

1

1
11

4

2

3

1

4

1

7
1

3

1

2

2

1

2

2

1

1
5

1

1

2

1

1

25
7

2

3

2

6

1

4

9

8

8

1

20

20

10
20

2

1

1

1

5

71

71

71
10

20

23

18

4

4

4

4

94
2614

1
37

1

1

31
14

3

2

2

1

3

4

2

2

2

4

4

6

6

1

4

1

1

18

2
18

1

1

2

1

2

1

1

1

2

1

2

1

9

3

1

2

1

5

5

5

1
5

3

1

67

4
65

43

2
9

1

3

1

1

1

3

1

1

1

1

2

2

2

6

3
6

3

252
24

32

1

30
11

4

1

2

5

1

5

1

1

2

2

11

2

2

9

8

1

3

4

1
28

7

4

1

2

11

3

6

1

1

9

9

14

10

10

4

29
3

4

2

17
2

3

1

2

1

2

6

3

17

17

4
9

3

2

1

1

3
25

2

3

1

3

2

1

1

3

6

3

3

8

4

4

11

7

2

2

24
1

2

2

7

4

3

3

3

2

4

66
2

2
21

7

1

2

3

3

5

1

7
1

1

4

1

2

1

1

4

4

16
3

3

2

1

2

1

1

1

4

1

1

3

3

3

2
10

6

1

5

2

2

2

73
834

3

3

5

4

4

1

3
5

1

1

1

9
2

2

3

3

2

2

2

12

7

5

4

1

7

3

4

1

1

1

1

6
3

1

2

2

4

1

3

3

58
7

1

28

1

3
18

2

1

2

2

1

1

1

3

2

1

2

1

1

5
22

6

8
4

1

2

1

1

2

3

3

10

4

6

1
7

4

2

1

1

11

3

8
2

1

1

3

1

2

2

2

3

3

4

1

2

1

19
5

1

13

6

7

2
1

1

1

3

2

1

1

2
9

3
1

1

1

3

1

13
1

4

3

1

1

2

1

1
4

2

1

1

7
2

2

1

2
1

1

1

1

6
1

3

2

7

1

4

2

280
72

1

1

2

1

1

1

2

1

1

3

1

1

1

1

3

5

7

1

1

132
19

1

1

1

1

3

1

1

1

3

1

2

3

2

1

1

2

28

1

2

1

3

4

4

2

1

1

2

2

1

1

1

1

2

1

1

4

2

1

4

1

1

1

1

2

3

1

3

1

1

3

1

1

2

2

1

4

1

2

2

3

2

1

2

6

1

1

2

1

2

1

3

5

4

1

12

2

1

2
7

2

1

1

1

2

2

1

1

4

4

32
7

1

1

1

18
6

1

1

6

3

1

1

1

1

1

4

4

9
60

3

21

27

4
2

1

1

10

2
7

1

3

1

2

1

1

1

2

2

2

3
1

1

1

3
25

3

16
3

2

3

2

1

1

1

1

2

1

2

4

1

3

9
49

1

2

1

29
12

1

2

1

1

2

1

1

1

1

1

1

1

1

2

1

6

3

1

2

2

10
1

1

1

1

2

1

3

1

5

3

3

1

1

33
2

8
1

2

1

1

2

1

2

23
3

2

3

10
5

1

4

1

2

1

1

1

1

1

28

3

3

3
25

2

1

1

2

3

9
5

1

1

1

1

4

25
1

5

5

6

3

3

3

4

4

9

3

4

4

2

83
1

1
2

1

1

11

8

3

6
1

1

4

4

10

10

10

10

16

5

5

2

4

4

4

4

4

3

3

4
6

1

1

10

7

3

874
31

1
16

1
7

2

2

1

1

1

1

3

1

1

1

1
10

2

2

5

2

1

1

1

6
15

1
2

1

1

1

1

2

1

1

3

3

1

2

1
28

6
1

1

1

1

2

5

5

2

8

1

2
6

1

1

2

1

5

1

2

2

9
243

3

3

1

1

4

1

1

1

3

3

1

2

1

2

1

2

1

1

2

200
8

2

1

1

1

3

4

3

1

1

1

4

2

1

1

1

1

1

1

2

7

1

1

51

1

1

1

1

1

1

2

1

2

1

1

1

61

1

2

1

3

1

8

2

7

2

1

1

2
3

1

1

1

1

1

1

1

2
4

1

1

2

2

496

3

493

5

1

1

1

1

1

1
147

2

2

118
25

3

1

1

8

2

5

34
3

2

3

2

3

1

1

2

1

3

1

1

2

1

1

1

1

2

1

2

2

2

1

4

3

1

2

1

2

2

1

2

1

1

1

1

4

2

3

3

2

2

8
1

2
6

4

1

11

4
1

2

1

3

3

1

5

1

3

1

11

11

2

1

1

2

2

2

2

1

4

2

1

1

14

10

10
2

4

2

2

2

2

2

2

1
309

10

10

10

298
17

9

4

5

19
120

14

2

3

2

4

10

3

2

5

7

1

4

3

11

5

4

2

19

2

2

3

1

5

2

4

8
77

2

2

1

1

7
54

1

3

6

1

2

3

1

1

3

6

5

3

4

1

1

4

2

2

1

2

5

3

3

24
3

5

7

6

3

5
48

4

4
18

3

7

4

6

2

1

2

5

5

605

605

605
4

528

73

55

18

978

1

961

1

1

2

1

1

3

6

1

3

3

3

1

2

120

120

8
120

29

13

9

7

14

10

10

11

9

7

17
16

1

5

5779
12

163

163

248
5604

68
10

3

2

9

4

4

9

1

7

5

2

3

11

3

4

4

11
28

1

1

4

3

2

5

4

1

1

6
1

4

1

7

7

12
4

1

1

2

5

7

7

4

4

4

28
2

12

2

9

1

2

2

3

2

3

2

10

10

16
7

4

5

5

14
49

3

1

1

4

4

6

2

1

3

2

2

1

2

1

1

3

2

74
328

1

1

4

4

3

5

1

55
3

1

1

2

12

8

4

4

1

8

1

1

9

3

4

13

1

1

5

1

1

2

5

7

7

6

7

1

1

5

2

2

14

3

4

1

2

1

2

10

7

4

5

5

6

1

3

2

7

3

4

2

3

5

8

4

11

11

7
33

10

9

7

7

10

10

10

14
106

4

12

2

24
3

1

14

1

1

3

1

4

5

8

3

2

2

5

9

2

2

2

6

23
3

3

6

2

9

9

549
2882

64

38

2

33

61

49

51

37

45

36
40

4

574
117

10

40

1

8

16

17

5

7

79

42

53

37

54

21

14

46

3

4

38

18

320

1

39

1

750

26

38

32

20

20

21

35

4

4

4
13

3

6

6

14

2

12

5

5

65
10

2

3

4

5

2

5

3

7

5

1

7

1

10

2

1

3

2

1

1

12
1

4

7

16

5

5

11

20
12

1

4

3

3
7

3

3

1

1

1538
402

117

268

268

171

52

52

305

223

2
30

6

8

6

8

41

41

20
1

1

1

2

2

1

1

10

1

1

1

1

21

21

20

20

13

13

7

7

83

1
16

5

1

5

4

3
67

4

1

1

2

1

1

1
30

1

1

1

1

26

26

1

1

1

1

1

3

3

1

1

1

1

1

1

1

21
4

1

10
7

1

1

1

1

1

1

1

1

2

1300

1300
83

10

10

3
14

3

2

4

2

11

11

7

7

17

17

7

7

37
10

13

6

8

20

9

11

11

4

4

8

8

1

1

5

3

2

5

2

3
2

1

711
127

25

3

1

6

5

13

1

14

15

6

6

10

10

11

4

5

1

24

4

4

21

6

13

19

6

7
6

1

8

2

16

3

6

157

6

124
12

6

8

1

14

15

3

10

18

22

15

22

4

2

2

9

9

11
7

1

2

1

17

7

5

5

29
3

8

7

4

4

7

54
175

1

1

57
5

3

2

1

1

1

2

4

3

3

9

1

2

2

2

1

1

1

2

1

2

3

1

2

2

3

1

1

1

2

2

1

1

1

1

1

2

1

3

1

1

1

1

1

1

1

1

1

1

1

1

1

1

2

1

2

1

3

2

1

4

2

1

1

1

2

3

2

2
18

1

2

3
8

1

1

2

1

5

22

7

5

10

11

11

6

6

8

8

2
31

7

5

10

7

2
19

9

1

2

2

3

15

3

3

3

12

1
12

4

3
4

1

3

37
3857

154

29
154

5

4

3

10

5

4

4

5

12

10
52

2

5

4

1

5

2

5

2

2

7

6

1

7

3

1

1

2

2

4

2

163
3186

6

2

2

4

5

5

8

8

3

3

95
19

2

2

3

8

2

1

1

10
45

4

1

4

1

2

2

8

5

2

2

4

2

5

3

1

1

2

2

5
120

64

11

11

40

25
7

4
8

1

3

10

349
1942

9

271
662

1

17

1

4

4

5

1

10

1

7

1

1

4

8

6

2

2

1

1

3

2

7

8

1

15

5

13

4

11

22

3

3

9

9

1

12

13

4

1

6

5

2

6

2

5

17

9

21

3

7

3

3

43

11

11

1

9

1

3

27

13

12

1

10

33

1

10

10

5

11

5

5

75

15

4

36

11

2

4

8

5

25

8

3

38

3

10

21

2

4

3

7

9

3

3

1

17

15

5

1

7

10

5

78

11

13

3

5

7

4

2

88

10

8

19

9

13

15

13

6

32

7

7

7

15

12

7

4

19

12

3

781
76

47

16

194
2

168

24

19

69

25

42

28

27

238

29

29

7

7

27

4
27

2

3

9

6

3

453
42

15

10

5

5

1

4

4

8

8

4
11

7

1

1

3

3

4

4

6

1

5
2

2

1

33
283

42

24

19

5

33

9

50

49
48

1

11

12

8

12

2

2

8

1

2

2

3

2

1
5

4
1

1

1

1

1
7

6

1

1

2

2

20
6

1

1

3
7

1

2

1

1

1

3

8
3

1

4

2
8

2

1

3

11

7

4

3

3

61

61

37
5

2

1

1

1

8

1

2

9

2

3

3

4

4

20
2

5

4

3

5

5

1

1
51

41

4
41

5

14

12

6

9

9

9

17

17

9

2

1

2

1

1

1

2

8
1

4

1

2

3425
69

7

7
1

2

4

22

22

107
1

10

6

4

4

17
86

4

2

6

10
1

1

1

2

1

3

1

4

1

3

2

3

1

3

1

1

3
1

1

1

1

7

3

1

5

1

5

2

7

7

3

3

19

3
19

3

3

1

2

1

1

1

1

5

1

8
479

43
194

2

1

4

3

2

7

5

6

7

3

2

2

23
93

1

2

1

1

2

2

1

1

3

1

1

1

1

2

4

1

4

2

1

3

1

6

4

1

1

3

1

3

5

2

7

1

1

2

5

5

1

45
228

1

1

1

2

1

1

3

1

1

2

2

2

2

1

2

3

2

2

8

1

3

1

1

1

2

37
1

2

2

3

3

2

2

1

4

2

2

1

1

7

2

2

2

1

2

4

1

12

1

2

1

1

10
9

1

1
2

1

1

3

1

1

2

5

1

2

1

2

1

1

4
3

1

3

1

1

4

1

1

1

2

1

1

1

3

9

1

3

2

4

4

3

3

4
22

3

4

4

2

1

1

7

20

13

7

6

7

582
2611

64
535

1

7

1

4

2

2

13

98
22

2

14

1

1

2

5

3

12

15

8

2

2

1

8

8

19

10

1

3

3

5

7

6

3

1

25

8

19

25

3

1

1

2

2

4

3

2

1

6

22

3

2

3

2

3

3

11

7

7

4

1

3

1

3

3

3

4

1

3

10

13

7

1

12

2

8

3

5

10

2

4

3

3

2

1

7

2

5

6
54

1

3
2

1

4

1

1

14
36

21

1

2

27
7

1

1

3

2

1

3

4

4

1

7

7

10
3

2

2

2

3

244
1389

16
1

12

1

2

18

1

1

3

4

3

28
5

5
4

1

3

3
2

1

7

2

2

1

4

2

3

2

1

2

2

4

4

5

8

8

25

1

6

2

2

3

2

82

3

2

12

4

4

5

2

3

1

5

2

1

1

1

8

1

1

3

1

3

66
7

2

1

7

4

2

4

7

3

2

3

5

1

6

1

5

3

1

2

777
52

5

5

2

3

1

7

1

2

1

1

1

7

2

11

1

18

3

3

3

1

1

3

1

2

7

2

1

13

2

4

3

6

2

5

2

1

1

5

1

1

2

1

1

1

2

6

6

2

4

3

1

10

156

4

6

3

1

3

7

5

251

1

2

5

1

1

19

3

3

1

10

5

1

1

3

2

10

13

1

1

3

1

4

8

1

7

1

1

17

9

9

8
3

3

2

77

77
18

1

1

1

1

2

16
7

1

1

1

4

1

1

1

1

1

1

1

2

1

1

1

1

1

1

1

2

1

2

1

2

2

1

1

1

1

11

1

13

4
13

2
1

1

3

1

1

2

4

4

4

131

2
131

17

16

2

1

9

4

1

38
5

10

1

6

4

2

6

4

7

5

2

20

20

20

47

31
46

8

7

1

1027

27

2

25

5

5

995

116
995

165

5

2

221

385

1

86
11

3

26

2

3

1

3

1

4

4

23

5

3

5

5

1

5
58

1

1

1

2

3

4

1

4

1

29

1

1

4

2

2

2

2

378
26

22

9

1

1

1

3

1

1

124

44

1

1

1

86

2

2

1

2

1

1

3

1

1

1

13

6

3

2

4

1

1

3

2

1

1

4

401497
7772

5
3

1

1

16

8

3

1

1

1

5

5

2

2

3

5

2

2

2

3

3

3

1

1

1

1

1

2

2

2

2

1831
363658

27
340

4

115

4

1

3

6

2

4

1

9

3

3

10

1

4

4

5

16

3

10

10

2

17

13

1

1

5

2

6

13

2

6

5

4

1

1

1

10

1

1

2

1

4126
360155

236
1954

4
174

47

24

14

2

7

3

21

7

18

13

17

32

12

14

6

6

8

30
121

2

82
22

1

4

2

6

4

1

14

11

6

10

1

7

54

13

35

6

16

16

66
359

15

8

18

10

9

1

5

15

4

198
20

10

17

5

4

50

7

16

8

3

29

29

10

45
163

2
18

6

2

3

1

1

3

40

33

27

11

11

3

6

1

1

44

23

7

9

5

456
61

5

7

3

10

2

12

2

30

4

2

9

6

262
60

29

18

11

1

9

7

7

7

11

7

18

7

6

7

12

6

7

1

1

10

2

7

1

3

7

1

8

1

4

5

5

8

1

2

2

1

3

13
2

3

2

3

3

3

3

3

6
40

12

14

14

8

58
9

3

29
14

2

12

1

1

1

2

1

4

4

4

1
30

4

2

2

6

14

5

162
31

5

6

4

4

2

106
24

2

1

16

18

2

3

1

1

16

1

4

3

12

2

4

81
1338

14

14

27

12

5

10

9

9

8

8

37
5

5

27

12
40

1

9
8

1

4

14

10

1

3

28

24

4

4

14

5

9

9

402
47

10

8

30

20

8

22

9
63

7

2

7

8

30

21

32

11

1

67

16

1

21

3

1

5

12

3

1

1

67

18

25

24

2

22

79
279

6

1

8

2

5

3

1

2

12
84

8

5

39

3

3

3

2

1

6

2

17

5

44

7

1

4

10

12
61

19

2

2

1

1
10

3

2

4

6

6

3

10
46

4

14

5

9

18

27

13

5

5

9

7

7

1

1

8

8

7

11

11

11

5

5

13
112

27

21
7

6

6

2

7

1

43

15
46

25

4

2

350156
157488

4

4

2

2

6
339

159

174

4
20

16

8

8

37
121

3

1

2

1

5

1

12

5

26

4

2

2

20
10

6

1

1

2

1
3

1

1

1

1405
683

11

128

44

12

1

26

156
9

8

11

9

15

20

2

63

8

11

16

33

48

16

2

9

8

1

21

10

12

11

21

25

18

9

36

14

10

11

9

4

16

13

3

120
512

35
26

9

8
90

43

12

4

1

14

2

6

18
33

15

187
195

2

1

5

16

23

584
1623

22

8

197

6

10

84
99

15

4

94

5

462
49

4

6

27

2

16

22

13

3

3

7

24

9

4

8

211

21

5

25

3

51
50

1

32
26

6

10

27

12

110

36

20

54

10

10

8

8

8

32

11

6

15

15

78015
17656

518

204
200

4

2367

5236

1154

1101

1252

185

4071
139

12

828

30

3062

112

2450

1535

300

3497

4850

2087

2455

383

712

526

2002

3245

5949

2561

328

3832

2350

5097

2

2

1415
82

249

249

232

311

74

173

15

200

79

7740
70381

499
540

41

115

1653
276

2

69
54

7

8

36

185
152

33

951
800

8

2

1

1

2

8

3

1

4

3

4

113

1

7

30

5

4

29

28

21

10

6243

279

1003
15873

2

23

13

5

242

37

2

2

13

33

3

10

7

7

12

4

100

1

7

1

129

580

3

7

47

130

1

9

9

7

173

16

81

200

6

138

243

7

15

12

16

13

26

104

638

89

96

76

10

9

415

103

52

13

3

9

85

7

18

2

4

16

2784

16

10

2

18

5

18

6

5

26

3

23

19

471

22

2

3286

63

10

5

30

18

479

40

4

14

13

9

4

9

12

4

9

1650

3

663

110

2

8

31

12

24

1

3

11

229

8

7

48

420

4

6

23

11

10

6

5382
977

1

98

396
313

3

77

3

26
5

19

2

10

113

199

62

437
460

12

7

4

34

680

7

70

888

204

316

33

172

11

301
319

10

8

46

138

122

22719

2510

161
152

9

3636

102

3427

1

7
112

35
87

52

18

38391
7669

92

28

315

63

82

223
218

5

36

125

221

110

258

15

179

305

153

24

151

93

523

42

72

194

40

40

30
34

4

401

244

74

61

2899
1246

1653

495

153

147

238
229

9

57

109

108

160

105

11

121

329

119

3813

115

41

662

14

371
7315

129

69

401

299

6

483

440

16

128

254

284

465

359

72

182

1

22

29

72

10

228

831

33

63

313

155

117

62

6

30

1

198

147

29

97

86

16

78

20

41

353

173

23

32

91

226

151

382

222

163

105

85

356

23

275

61

130

29

139

69

67

64

198

181

452
609

2

155

329

171

152
148

4

192

4088

86

182
183

1

61

564

152

1

1

146

146

175
13

5

8

4

2

1

3

1

66

4

11

7

1

1

7

5

3

14

2

1

11

4

1

1
57

2

2

8

8

40
2

2

2

4

4

24

24

8

6

1

1

5

5

103
1812

5
119

23

11
78

18

10

1

1

3

4

5

9

14

1

1

2

1

5

5

8

8

27

24

24

3

6

5

1

6

3

3

3
62

1

15

5

3
23

3

1

5

2

3

4

1

1

15

20
96

2

2

1

2

6
13

7

4

52
8

21

8

12

3

136
30

105

1

45
2

1

5

3
37

7

1

11

2

5

3

5

8

8

5
115

17
2

2

2

2

9

3

7

83

5

3

2

2

449
86

300
56

3

1

3

2

19

12

1

1

15

1

2

4

67

3

6

4

15

2

15

1

13

1

1

2

9

1

2

1

6

10

11

10

3

42

4

14

10

10

1

1

28
138

5
65

1

15

7

9

7

10

2

1

3

4

1

2

12

1

5

3

1

5

13

2

1

89
10

1

10

9

59

3

16

14

8

2

1

3

2

8

2

23

3

6

1

4

9

6

6

2
7

1

4

4

33

31

11

7

9

4

2

2
1

1

4
1

1

2

2

11
15

2

2

1

1

23

2

17

7

10

4

3

3

7

7

18

11

11

7

2

2

2

1
82

1

14

4

2

5

55
5

2

1

15

3

1

9

6

13

9

1

8

7
11

4

9

9

37
3

9

25
3

8

4

8

2

1
17

3

3

10

3

67
3

14
64

11

1

17

1

1

3

2

1

1

7

1

3

1

12

6

4

2

537
31

1

1

1
36

16

2

10

4

5

14

10

8

8

2

15
49

31

1

11

3

2

8

6

1

1

1

4
36

3
31

1

2

3

6

12

4

1

51

11

1

7

4

21

12

2

4

3

7

18
1

12

4

1

1

4

4

17

2

2

3
13

4

2

2

1

5

69

57
4

35

3

6

1

8

12

11

5

1

5

1

1

3

28
1

9

16

2

3

10

1

2

22

18
6

3

3

1

5

4

18
7

3

1

5

2

8

3

4

1

13

8

5

73
5

19

47

2

13

4

28

2

27
1

1

1

12

2

11

355
18

11

8

8

3

1

2

2

23
32

5

4

4
44

6
3

1

1

2

10

7

3

3

12

1

10

1

12

5

7
2

1

4

26

9

4

5

5

8

8

6
5

1

1

3

3

4
84

3

3

42

42

10

2

3

2

1

5

7
5

2

2
8

6
2

4

10

4

6

88
6

3
26

2

20

8

4

1

7

1

8

3

3

5

3

3

1
45

4

3

2

7

4

1
22

1

1

8

10

1

2

10

10

4

6

42

3

3

1

1

38

1

32

32

5

109

2

2

2

1

1

96
6

2
77

17

13

11

4

18

8

4

11

2

4

4

4

4

4

2

2

11

11

11
3

3
8

5

33

33

400
30

9

9

9

140

4

4

17

10

7

6

6

3

3

1

1

1

28

1

27

3

3

42

1

41

7

7

9
1

1

1

3

4

1
20

4

3

5

4

3

7
168

12

1
12

11

16
98

3

36

3

8

10

2

1

2

10

7

7

6

23

29
4

1

1

1
19

3

3

3

2

7

3

1

22
1

3

1

17

1

8

6

2

1

1

52

2
47

42
1

7

2

27

1

4

3

1
5

2

2

424
3

39
276

3

3

24
5

6

7

7

6

5

5

4

1

3

3

53
27

2

17

7

13
18

1
5

1

3

5

1

4

3
33

1

1

3

26

25

1

8
16

5

3

3

16
9

1

1

5

1

6
2

3

3

1

1

1

39

6
34

1
6

5

13

3

3

3

2
5

3

10

7

3

3

3

2
142

2
3

1

9
1

8

8

2

2

2

2

2

18
4

13

1

53
13

1

5

12

12

1

4

7

2

5
6

1

2

6

6

3
2

1

1

14

14

8

8

18

1

17

16

4
16

4
12

2

5

1

487
15317

1551
77

58
753

16

13

13

3

5

4

1

2
6

2

2

2

16

9

9

7

14

14

5

5

52
163

1

4

6

2

3

3

2

1

4

5

6
35

2

1

1

3

3

5

1

2

4

7

1

16

3

8

3

1

4

2

7

9

9
2

7

3

3

4
12

2

6

6

13

13

1

1

1

1

1

19

19

2
5

3

3

6

6

6

6
16

6

6

4

23
9

2

6

4

3

1

2

3

3

50

37

20

17

13

28
16

6
3

1

1

1

5

1

4
14

9

1

1

6

6

9

9

2

1

1

14
4

2

5

5

3

16

16

2

2

1

1

3
6

1

2

3
1

2

6

6

10

10

1

1

2

1

1

1

2

2

4

4

7
20

2

1

1

3

2

2

2

2

1

1

115
39

1

5

1

1

1

1

2

1

48

1

3

1

2

1

1

1

7

1

2

2

6

6

1

1

38
6

3

18
3

2

8

5

11

1
96

3

32

11

2

27

1

1

12

6

432
12

2

2

23
102

1

3

6

12
11

1

1

8

3

5
31

3

2

6

1

1

1

3

5

4

6

3

5

19

4

15

5

5

8
1

1

1

5

5

1

4

4

1

1

2

9
1

3

5

5

2

3

21
26

3

3

2

24
4

5

1

2

1

1

5

1

2

6

1

16
37

8

10

1

2

2

5

5

6

6

3

1

1

2

6

6

6

3

3

28

1

1

27

7

2

2

1

2

3

3

5

5

1

1

5

5

15

15

4

3

1

33

5

21

1

5

1

2

2

3

3

4

4

15

9

6

6

5
7

2

3

3

5
2

1

1

2

3

2

1

2
1

1

6

2

4

1
2

1

46

2
30

17
5

4

8

11

3

3

13

13

4

4

2

1

1

2
119

83
3

1

56
3

27

8

18

23

34

11

11

23

24

4
24

14

2

1

1

3

11

1

10

11

11

1

1

7
10

1

1

1

1

5

1

1

4

4

3

1

19

19

4

1

3

1

1

3
13

1

2

2

4

1

1

1

1

1

1

1

770
9

7

1

1

1

1

3

59
735

1

1

2

2

2

2

2

1

1

1

1

1

1

4

2

1

1

2

1

1

1
6

1

2

2

1

1

1

1

1

1

1

1

1

15
3

1

10

8

1

1

1

15

2

1

3

5

4

1

1

2

1

1

1

1

1

1

7

1

6

1

1

2

1

1

1

3

3

10

1

4

4

5

4

1

3

2

2

1

1

1
9

1

1

1

1

1

1

1

2

1

1

28
1

2

5

1

1

18
4

2

2

9

1

2

1

1

1

31
1

2

1

12

3

1

1

6

1

3

2

2

1

1

1

1

3
25

4

5

4

5

5

4

4

2

2

2

1

1

5

5

8
24

1

2

2

1

1

8

2

1

73
12

4

1

1

1

1

1

7

2

2

2

3

1

1

3
20

1

1

1

1

1

1

1

2

4

1

1

1

1

2

1

1

5

4

1

1

1

2

2

2
8

5

1

1

20
2

7

4

5

1

1

3

1

1

1

1

20

4

5

5

1

6

3

1

4

2

1

1

2

1

1

1
12

3

8

7

1

1

1

1

1

1

2

2

7

6

1

2

1

1

3

1

1

1

1

1

4

1

3

1

1

1

1
10

1

1

8

1

1

1

1

4

1

2

1

1

1

1

1

13
2

2

3

3

1

2

1

1

7
2

2

1

1

2

1

2
1

1

5
1

1

3

3

1

1

1

1

2

1
2

1

5

5

38

7

3

4

31

5
1

4

1

1

1

1

63

1

1

62

1

1

4

1

3

2

1

1

1

5

3

2

1

1

2

1

1

1

1

1

1

1

1

1

8

1
8

7

3
1

2

2

4

1

1

17

12

12

4

1

5

1

4

7

1

1

1

1

1

1

1

6

1

1

1

1

2

1

1

1

1

1

1

4

2

2

3

8

1

7

2

2

1

1

1

1

1

1

1

14
5

2
6

4

3

4

4

1

1

5

5

7
4

3

3

1

1

1

1

1

9

3

6

6

19

19
1

3

3

10

5

13

13

13

9

4

4

5

5

1

1

2

1

1

2

2

4
1

2

1

1

1

1

1

1

1

1

2

2

2

1

1

52
2193

98
8

1
25

3

3

1

2

21

2

16
10

1

1

1

1

1

1

2

1

2

2

17

1

1

2

8

1

3

1

1

1

1

3

2

2

2

2

1

1

1
6

1

1

3

13

1

8

2

1

1

2

1

1

5

1

1

1

1

1

1

1

1

1

14

13

1

2
27

15

9

1

2016
117

1

1

6

1

2

1

1

3

5

5

9

9
2

6

1

11

1

10

1

8

1

6

1

3

2

2

1

1

1

1

129
17

7

2

1

3

2

1

64
12

1

1

1

10

2

3

2

11

1

1

1

1

7

6

1

1

1

1

1

1

2

4

8

3

1

6

1

1

1

3

7

7

7

2

2

5

11
1

8

3

5

2

2

2

21

8

5

7

3

4

1

1441
306

4

5

1

138
604

6

2

7

3

2

8

14

1

4

6

1

1

1

33

1

2

10

1

9

7

2

1

2

5

2

2

1

5

1

2

1

3

2

5

4

1

5

2

1

15

3

3

4

2

3

18

8

4

4

2

2

1

6

1

4

5

5

1

4

5

1

5

9

1

3

1

1

11

6

1

5

1

4

11

1

6

2

2

5

7

3

3

1

1

50

3

1

2

5

1

3

2

2

1

4

8

1

6

4

1

1

2

1

2

6

3

4

1

14

24

3

4

10

5

2

2

9

24

10

3

7

3

4

13

4

16

2

5

3

5

8

5

4

37

13

1

2

4

6

6

6

1

16

1

7

7

1

11

5

3

5
4

1

2

2

3

2

13

1

1

8

1

14

1

12

1

29

16

2

15

1

3

9

3

6

4

8

2

10

3

2

3

7

5

4

2

9

1

1

8

1

1

1
8

1

1
5

3

1

1

16
1

12

5

7

2

1

148
5

1

9

1

1

22
74

1

2

1

1

1

3

1

6

5

1

1

1

2

4

1

5

1

2

1

4

4

2

1

1

1

6

2

1

1

5

3

2

5

1

1

2

3

2

1

3

6

12

4

2

2

2

8
46

1

26
2

1

10

1

1

2

1

1

1

2

1

1

1

1

1

2

1

7

7

3

4

1

1

10
231

1

3

1

138

1

1

1

1

1

1

1

4

1

5

1

1

2

1

7

1

1

5

1

7

3

1

9

1

2

6

2

3

2

5

181

10

3

4

3

1
171

7

7

46
2

1

4
27

1

15

2

1

1

3

10

1

3

2

11
42

2

2

1

2

1

4
12

1

1

1

1

4

1

5

1

2

1

1

3

3

2

1

1

5
62

4

2

1

2

1

1

7

8
39

1

1

1

1

2

1

1

1

2

1

13

2

1

2

1

1
8

7

1

5

1

29

8

1

1

6

1

5

5

1

1

21
2

1

1

5

5

5

7

7

2
4

2

1

1

1

1

1

1

1

1

546

1

1

1

538

536

536

2

1
2

1

1

2

2

2

2
1

1

5

5

11

1

1

10
4

3

1

1

1

3

3

2

1

36

10

3

7

5

2

1
21

6

6

2

2

10

1

1

1

4

1

1

1

3

2

1

2

2

2

9209
447

10

4
10

1

3

1

1

820
23

3

3

9
14

1

1

2
3

1

62
19

2

1

4

3

1

4
21

3

12

2

10

1

1

1

3

3

3

1

1

1

595
92

9

2

1

6

5

4

1

1

1

3

1

3

4

1

1

2

8

1

21

94
417

1

1

2

10

6

2

1

1

1

1

3

2

1

1

2

1

4

1

1

1

1

1

8

1

1

8

2

1

1

1

4

1

4

1

1

1

6

5

1

5

4

5

1

1

1

1

1

9

201

1

1

5

2

2

2

1

1

3

3

7

5

5

2

27

15

5

5

3

4

1

1

3

3

1
18

2

2

1

1

6

4

2

4

1

12
30

1

17

1

6

1

9

3

3

3

13
1

5

2

2

1

2

1

3

1

7

4

3

1

3

5

5

11

6
1

4

4

1

5

4

4

1

17
120

5

2

1

4

1

45

8

2

14

5

4

2

3

6

1

16

1

1

3

1

1

1

12

9

9

3

182
7

5

5

22

9

6

2

1

13

1
46

7

13
34

2

12

7

4

32
15

2

2
15

1

12

25
11

5

1

1

8

15
5

2

3

5

1

4

5

5

25
1

5

19
4

7

3

5

128
1376

50
22

2

17
10

7

3

6

1

2

3

232
899

3

5

1

12

37

3

2

3

3

7

61

6

2

3

3

3

2

2

1

1

5

4

4

7

250
37

10

1

16

7

3

2

20

2

1

1

1

8

1

1

1

2

1

5

3

29

5

1

2

1

5

5

2

1

1

10

1

2

1

1

2

2

4

2

1

1

2

1

2

2

1

7

3

5

2

3

3

5

3

4

1

1

1

1

1

8

1

2

5

4

2

3

1

1

11

162

2

6

26

3

7

7

111
20

1

1

1

4

82
21

14

3

4

1

3

1

1

1

7

1

1

2

1

3

2

1

3

5

1

3

3

2

1

1

76
25

9
1

1

1

4

1

1

1

1

40

19
3

9
16

1

1

5

1
38

1

3

26
3

7

5

6

5

3

4

4
47

14

29

77
5

29
9

6

1

12

1

1

1

29
14

1

2

1

1

1

3

1

3

3

1

1

5
4

1

4

4

1

1

4

4

63

3

3

1

1

1

2

2

6

6

3

3

12

5

5

7

5
1

1

2

2

1

1
19

7

11

11

4

4

1

1

5

2

3

3

6

5

5

1

19

19

19

46

37
8

3

3

2

1

6

16

1

9
3

3

3

53

53

53

7

7

7

50
1126

11
175

35
1

1

3
10

3

1

1

1

1

10

2

11

17
129

5

5

3

1

1

1

2

5

92
89

3

2

3

6

88
793

2

1

1

1

1

1

3
42

1

7

1

1

6

9

2

1

1

3

2

3

3

11
1

1

9

88
642

3

1

1

2

1

4

1

4

11

43

4

1

204
61

1

1

1

2

2

1

1

1

1

4

1

6

8

4

10

1

1

1

7

7

2

3

5

10

9

17

3

2

2

3

2

3

1

3

1

4

1

3

1

1

1

1

1

2

1

1

1

198

6

5

2

5

1

3

4

2

1

1

3

1

3

6
7

1

1

1

5

1

1

1

11

3

5

1

2
18

5

1

1

1

2

10

1

23

1

7

13

2
1

1

1

1

4

1

2

1

7
58

7

4

3

1

4

36

3

3

3

13

13

58

38
6

5

11

16

16

20

8

12

3
308

10
1

3

6

7

7

14
1

2

6

2

2

1

1

1

3

1

9
12

1

2

10

7

3

2

2

1

1

64
6

4

1

46
22

2

2

5

2

1

2

10

2

1

4

16
5

7

1

3

2

2

27
4

4

11

11

2

6

2

9

9

129
53

4

1

11
47

1

4

3

1

7

1

1

5

1

1

1

2

1

3

1

1

1

1

1

1

1

2

1

3

10

1

2

1

1

32

32
3

18

11

195
21

57
4

8

3

1
33

4

1

7

2

2

3

6

7

4

5

3

3

29

7
29

2

10

10

23
9

14
5

1

1

2

2

3

1
26

11

6

8

13

13

23
8

1

1

12

12

2

296
15

6

6

5
22

1

1

7

2

3

2

4

4

11

11

1
15

10

9

1

4

4

2

1

1

3
13

4

4
2

1

1

1

1

26
210

1

13

6

7

39
10

2

2

2

1

3

6

4

3

6

5

16

8

82

5

2

2808
269

25
100

1

1

23

12

2

19

10
8

1

1

8

2

2

2017
548

5

17

16

286
963

2

1

72

8

3

2

2

1

13

3

2

29

31

14

3

2

7

9

2

15

2

8

1

2

7

8

1

19

2

3

6

7

1

2

6

2

1

4

3

4

4

18

1

2

3

8

4

2

13

5

55

9

71

3

7

32

3

4

13

25

1

2

4

35

10

3

5

3

1

1

17

2

1

19

14

21

20

38

1

10

54

15

2

11

1

10

59

5

11

34

11

17

5

11

68

9

16

6

56
220

6

13

3

5

7

5

1

4

1

8

1

12

2

3

6

6

19

2

27

39
2

4

2

2

8

1

10

1

2

4

3

7
200

98

95

95

1

1

1

37

3
12

3

6

7

7

8

8

1

1

9

9

4

4

4

81

81
10

36

35

25

25

4

21

21

35
991

450
391

3

56

1
218

11

184

181

3

22

6
48

9

20

13

13

49

31

31

18

32

32

72
6

27

27

8

14

17

36

5

31

4
51

31
30

1

16

113
2

1

2

6

2

10

1

2

4

1

2

79

1

13

13

2

2

2

11

11
3

3

3

1

1

3

1

1

1

1

2

950

25
900

1

1

1

340

339
1

3

3

9

8

7

1

1

1

1

4

4

10
318

1

302

301

1

1

1

1

1

1

1

2

2

1

1

1

1

1

1

68

2
48

30

1

29

29

1

1

1

1

1

1

4

3

1

9

6

1

2

18

3

3

3

3

2

1

1

10

4

1

5

2

1

1

1

1

1

1

16

2

2

2

2

6

3

3

2

1

1

1

1

1

1

1

8

4

4

2

2

1

1

1

1

1

1

120
5

2

1

1

1

12

1

1

2

1

1

4

81

1

1

1

1

1

1

1

270
6

12

4

1

1

3

3

8

5

2

2

3

3

3

94
1

6

4

4

2

2

1

1

68
2

1
12

3
5

2

6

1
25

4

13

13

7

1

1

28

28

8

8

8

10

10

10

30

18

7

1

2

2

1
125

2

2

2

73
6

11

11

5

1

2

2

6
10

1

1
2

1

1

41
11

2

1

6

1

2

1

16
1

1

2

12

1

38
2

5

5

12

8

4

1
5

3

1

1

1

10
3

5

2

3

3

3

11

11

8

1

6

1

3

3

3

3

2

1

1

1

1

1

55

8
1

5

1

4

1

3

2

2

1

1

1

37

1

1

6

3

3

3
1

1

1

5
26

3

1

4

4

9

1

1

3

3

1

1

1

3
9

6

50

50

41

41

1

1

40

8

1

1

6

1

5

5

5

1

1

1

1

1

3

3

612
13631

33

13
5

3
6

2

1

1

1

1

1

1

1

1

1

1

1

9

1

1

1

2

1

1

1

1

1

3

3

1

1

1

1

2

2

2

7
2

5

86

84
5

5

5

2

2

4

2

2

2

24
2

7
22

15

1

1

1

3

3

1

1

1

1
18

1
17

1

15

2

2

2

2

7

5

1

1

10

8

8

2

2

2

2

96

3
96

5

5

1

1

1

1

4
1

1

2

67

66
12

54

52

2

1

1

1

2

7

7

3

3

2

10

10

10
1

9

3643
12

2313
1

1

1

9

1

1

8

4

4

558
2297

1

2

11

7

1

1

3

2

2
375

7

6

146

1

164

1

43

5

1

2

10

9

2

1

159

6

22

23

2

4

1

3

1

7

241

207

3

1

1

5

10

9

1

1

2

12

37

6

6

18

4

2

1

1

1

3

1

3

307
102

1

1

4

4

1

1

2

2

2

3

3

3

15

3

5

1

3

1

1

3

1

1

3

1

2

12

1

2

2

6

1

1

2

1

3

3

3

7

2

2

1

1

8

1

1

1

1

4

1

5

2

1

1

2

6

3

3

1

3

1

3

4

1

1

1

1

4

2

1

3

5

3

1

1

1

4

2

2

3

6
57

2

43

43

6

4

3

8

4

1

3

6

2

4

1

15

1

4

2

6

1

1

1

1

33

33

4

1

4

5

3

1

1

4

2

262
36

145

145
85

60

16

44

48

32
9

4

19

16

11

2

20

2

1

8

1

6

11

3

3

1

6

2

5

4

1

1

1
1308

11
1255

6

855
4

845

6

1

325

5

1

3

1

2

2

4

2

7

22
3

1

4

13

1

1

7

1

1

1

1

5

1

4

8

5

2

1

36
15

8

2

11

1

1

9

1

1

8

8

8

2

1

1

5

5

2

2

3

1

2

180

94

16

5

11

1

1

18

1

17

3

3

31
2

7

7

1

7

4

10

1

1

12

5

5

7

8

8

4

4

86

83
9

6

3

3
62

57

2

3

3

3

9

9
1

8
2

2

4

2

2

3
38

17

17

4

1

1

1

1

5

1

1

3

6
16

10

10

2

2

6

6

4

4

2

2

390

118

118
11

2

1

2
99

1

68

5

23

5

1

1

21
8

2

1

1

1

8
1

6

1

3

1

2

92

90

90

90

2

1

1

1

13

13

1
3

1

1

3

5

1

1

1

1
119

4
10

1

1

1

4

4

63
11

7

1

4

4

2

2

1
20

1

4

1

5

2

1

3

2

8

4

5

5

17

17

2

2

5

5

2

2

1

1

3

3

3

1

1

1

4

4

3

1

1

1

2

1

1

1

9
19

7

5

1

1

1

1
3

2

7

7

7

71
1

10
1

3
1

2

1

1

2

2

1

1

3

2

1

1

5

2
5

1

1

1

1

1
13

1

1

4

4

1

3

1

1

1

1

2

2

3

3

1

1

1
38

1
13

4

2
8

1

4

1

17

1

16

16

2
1

1

1

1

2

2

2

2

3

3

90

90

1

1

13

2

11

76

4

61

2

2

1

1

4

1

199
6

3

3

3

16

1

1

15

15

15

4

4

1

1

3

5

1

4

79
6

19

12

1

6

9

7

7

2

1

1

1

1

2

2

2

2

7
9

1

1

1

2

2

1

1

2

2

1
5

2

2

1
2

1

1

1

15
1

1
5

1

2

1

2

7

2

2

5

5

5

1

1

1

1

1

5
67

3

3

6

6

3
4

1

1

1

1

1

5

1

4

5
1

1

3

1

1

1

1

1

12
1

2

9

1

1

5

3

2

2

2

11

11

2

2

1

1

5

5

5

9

9

9

1259
13

881
21

2

1

1

11

11

1
39

9
13

2

2

21

4

10

10

86
15

1

2

2

1

1

1

2

1

1

2

1

1

53
4

7

1

1

1

4

35

1

1

13
98

3

2

2

7

1

1

10

1

1

4

1

4

1

4

36

4

3

1

5
4

1

24

1

16

6

1

105
22

57

57

3

15
4

1

3

1

6

7

1

51

3

2

1

3

1

1

10
35

15

1

1

5

1

2

5

434

434

3
25

5

17

340
7

1
48

13

5

4

1

1

2

5

4

16

9

1

1

7

7

10

1

1

1

4

4

5

5

4

4

1
67

1

5

1

9

3

2

45
10

2

1

7

4

6

1

1

2

2

1

8

5

5

6

2

4

4

13

10

2

1

1

1

49
157

67
4

1

9

9

12

4

7

4

2

8

5

2

6

6

2

5

1

14

7

6

4

4

2

3

3

8
550

1
62

1

1

1

1

3

1

2

10

3

7

10
18

1

1

1

5

2

1

1

1

2

2

2

1

1

2
16

6
3

2

1

1

1

1

2

3

3
7

2

2

2

7
407

9

9

3

3

231
34

1

1

1

1

3

1

1

85

1

1

1

1

1

4

3

1

1

10

1

1

7

55
12

4

1

3

1

4

1

8

1

1

14

1

4

1

3

6

5

1

2
30

2

6

1

15

2

2

1

1

7

5

5

2

2

11
1

1

5

1

3

6
99

93

3

3

1

1

5

5

1

1

1

6

3

2

1

1

3

1

1

2

5

5

5

19

19

12

7

7

5

5

2

3
1

2

1
17

11
1

3

4

2

1

1

3

3
1

2

2

1

1

19

3

1

1

1

16

1

1

6

1

8

1

1

1

5356
45

21

21

16
21

5

15
4

11

11

145
20

10
51

5

5

3
4

1

1

3

28
3

1

4

1

3

3

11

1

1

1

20

1

1

19

5

5

3

3
1

2

40
2

8

2

7

4

9
5

4

8

2

2

4

4
244

10
167

2

1

1

151

2

27
17

2

3

5

44

4

24
4

1

7

12

15

1

2

2

3

1

1

1

1

1

123
4268

15
1154

5

7

4

1047
1114

67

9

2

1

1

6

6

4

4

1

1

3
1

2

15
8

7

5

2

3

3

466
3

5
12

2

2

1

1

1

2

1
446

39

1

1

15

389
21

59

77

2

228

2

3

4

1

1

1
5

4

4
2

2

17

4

11

11

2

13
1524

1

6

1485
1488

3

16

8

8

1

1

1

1

1
23

4

3

15

3

1

2

2
1

1

907

469
483

5

5

8

8

1

424

13

2

12

45

3

1

2

3

303

20

20

15
183

23

12

12

8

3

7
1

4

4

2

3
23

3

1

2

16

1

4

4

17
88

45

15
4

2

9

6

1

3

1

6

6

2

2

15

15

15

26

11

11

14
4

3

5
7

1

1

1

1

406

13
19

1

4

1

3

3
1

2

8

5

5

15
338

323

33
27

6

13
148

1

4

1

83

6

1

1

1

1

2

4

1

1

7

1

1

1

5

1

1

1

4

1

1

1

1

2

759

5
759

5

5

1

1

1

1

1

1

493

3

2

2

470

8

8

8

2

11
248

11

3

6

1

1

1

2

1

3

2

20

1

51

19

1

1

10
53

40

40

40

3

63
1

25

4

28

5

2

1

1

5
3

2

2

91

1

1

4

1

1

5

5

5

1

1

4

2

2

1

1

28
2

7

7

3

1

2

2

13

6

7

3

3

3

11

1

10

12

1

11

18

18
3

1

1

1

6

6

2
5

1

1

1

1

1

1

44

4
12

6

2

9

9

9

9

5

4

4

23

22

22

1

1

2

2

1

1

1

1

1
17

1

2

11
3

2

1

1

1

3

2

1

1

1

1

1

1

1

1

1

3
809

12
222

3

1
3

1

1

1

30
1

7

7

1

1

5

5

1

1

3
1

2

19

18

1

1

1

6

6

5

3

2

1

1

3

2

1

1

1

1

1

1

1

55

52
1

5
41

8

8

3

1

1

1

1

1

6

6

1

1

1

8

2

4

1

1

1

9

9

8

1

7

2

1

1

1

1

3
1

1

1

1

1

1

1

1

28

25

4

4

21

16

15

1

1

5

5

5

2

2

1

1

1

93

1

1

6

74

4

1

2

1

1

1

1

11

11
3

2

1

1

1

1

1

1

7

1

6

2

1

1

1

1

1

1

4

3

3

3

1

1

1

13
559

3

1

1

2

2

1

1

1

1

1

9
450

115

8
115

4

4

31
15

7

7

9

32
2

14

16

11

5

1
26

12

9

4

14

4

4

6

6

9

5

4

99

21

14

4

2

1

78

2

2

13
1

12

12

6

5

1

5

2

3

3

2

1

1

19

19

14

1

4

2

2

1

1

2
5

3

4

4

1

1

3

3

9

9

182

9
182

20

20

24

24

25

25

8

8

6

6

9

9

20

20

15
47

14

9

9

9

14

14

36

32
3

2

2

4

1

1

2

4

1

2

1

1

1

8

5

1

2

2

2

1

1

2

2

4

4

1

1

4

1

1

1

1

2

2

41

9

3

3

6
1

3

1

2

2

2

2

3

3

5

1

3

1

24

24

24

24

52
2

1

1

1

1

1

1

40

1

1

2

3

3

3

3

3

3

3

3
1

1

1

1

1

10
649

21

20

20

7

7

13

13

1

1

7

4

4

4

3

3

10

1

1

9

9

9

1

1

1

224
1

8

4

1

3

54

21

11

11

10

10
1

7

2

14

14

19

11

11

8

8

2
161

155
9

21

1

1

3

12

1

1

3

4

4

5

5

3
32

1

1

5

11

12

4

4

4

4

19

1

9
16

2

5

2

17

1

6

10

3

7

2

2

2

2
1

1

1
28

2

2

3

1

3

1

11

2

1

3

3

1

1

4

8

8

4

1

3

1

1

2

2

2

373
26

302

6

2

10

1

1

1

1

1

1

3

4

1

7

1

4

1

10

10

1

1

1

1

9

1

1

1

2

2

2

2

6

1

1

1

1

1

4

4

365
1

1

1

1

2

2

2

1

1

1

1

1

1

1

1

1

1

1

1

1

1

352

2

2

2

2

2

2

2

2

350

350

350

350

1
334

116

116

6

3

3

2

1

9

9

177

177

6

6

19
1

3

11

1

1

8

1

3

1

15

5
15

8

2

1

1

1

1

3

3

3

3

3

2
1

1

1

1

1

1

1

1

1

4

4

4

4

4

4

1

1

2

1

1
