# Supplementary material for: Metagenomics of African Empogona and Tricalysia (Rubiaceae) reveals the presence of leaf endophytes
Source: PeerJ. 2023 Aug 4;11:e15778. doi: 10.7717/peerj.15778 (PMC10405798; doi:10.7717/peerj.15778)
Supplement: Data S1 [file peerj-11-15778-s003.zip › Tricalysia semidecidua.html]

Javascript must be enabled to view this page.

members
magnitude
magnitudeUnassigned

kaiju.out

2000000

598179
81

5513
597983

2
174

39

1

1

1

1

1

1

38

24

4

2

2

2

2

2

2

2

14

14

14

14

6

6

1

1

1

5

5

14

10

5

5

5

5

5

5

5

4

4

1

1

1

1

2

2

1

1

1

1

1

1

1

1

2

2

2

2

1

1

1

1

1

127

4

4

98

4

4

94

94

24

24

24

1

1

1

1

1

1

585900
14387

4

4

4

4

4

4

133

3

3

3

3

13

8

8

8

5

5

1
59

11

46

1

58

58
1

57
7

1

1

1

1

4

4

6
20

2

8

6

2

3

1

9

9

9

2

7

7

6

2

4

2
3317

1

1

1

1

1

1

1

1

1

15

9

9

6

6

5

4

1

2

1

1

1

1

1

1

1

3296
5

20

20

20

20

19
2

17

16

1

1

1

16

3
16

6

6

6

7

1

6

3253
22

73

73

7
73

1

1

16

12

12

4

4

3

1

1

5

5

5

1

1

1

5

5

30
5

1

13

3

8

8

2

2

2

2

1045

1

1

1044
1

1016
2

896

896

9
3

1
4

1

2

1

1

88
13

1

74

6
2

4

15
1

1

5

1

1

4

2

1

1

1

1

27

27

3

3

1

1

2

1

1

1

1

3

3

3

3

255

255
1

28

28

28

11

11

1

1

1

34
3

1

1

1

4
3

1

26
7

13
1

5

1

2

1

2

1

2

1

1

1

1

1
177

2
3

1

1

1

1

7

7

2

2

8

8

8

1

1

1

2
25

1

20

20

2

2
20

1

7

9

1

1

1

99

99

99

1

1

1

7

7

2

2

2

1

1

1

33

8

3

17

1

1

3

4
1406

2
14

3

1

1

1

2

2

2

2

2

4

4

2

2

3

3

1

1

1

1

1388
103

1019

495

495

524

524

15

13

13

2

2

251
2

54

50

34

16

4

178

178

17

17

17

413

413

7

7

244
1

1

1

4
3

1

2

2

35
2

1

19

3
13

1

7

1

1

3

3

3

1

1

8

8

1

1

2

2

1

1

183

20

2

2

70

91

1

1

1

1

1

1

1

1

46
1

38

38

1
6

5
4

1

1

1

1

115

115

115

2

2

2

2

2

2

8
1

5

1

4

2

2

2

2

2

39
2

3

2

1

34

1

1

1

1

1

6

6

6

6

27

2

2

25

8

8

1

1

1

16

16

9658
371

17
5524

7

7

7

7

7

4

4

1

1

1

3

2

2

1

1

1

62

61

60

60

60

54

6

1

1

1

1

1

1

9

6

5

5

5

5

1

1

1

3

3

1

1

2

2

5424
437

77

39
1

8
1

7

7

6
18

1

3

6

1

1

1

2

1

1

1

12
1

1

10

18

18

1

1

1

1

9

6

4
17

1

1

1

1

1

3

3

4

1

3

2

1

1

1

1

3

3

2

1

352
6

24

24

1

1

10

12

6
173

140
2

1

7

1

6

126
2

1

116

2

1

1

2

1

1

1

1

1

6

6

10

10

11

1

1

1

1

2

3
5

2

9

9

9

131

3
66

43

43

1

3

6

10

10
64

1

1

4

6

22
3

9

4

6

20

1

1

9

2

2

1
7

2

1

2

1

1

2068

16
2068

5

2

2

3

2047
273

1

5

2

14

56

93

2

1

3

1

1

83

3

40

18

7

8

1

96

2

74
317

5

2

7

103

1

14

1

1

2

1

1

1

1

1

1

2

86

1

2

6

1

1

1

1

46

43

154

91

7

8

1

554

1

2

1

44

68

1025
26

13

1

1

11

1

10

10

1

1

3

3

1

1

2

34

34

1

33

1

1

1

10

9

1

1

12

11

11

1

1

1

5

5

5

31
765

1

1

1

1

1

1

2

2

2
137

1

134
7

49

1

3

64

1

1

1

5

2

1

1

1

580

580

7

7

4

1

1

3

8
148

38

36

2

2

2

2

1

1

1

4

3

1

1

2

6

6

1

1

1

2
24

12

10

6

1

3

1

1

51
2

48

4

37

1

5

1

1

2

2

2

2

2

1

1

2

2

2

2

2

2

8
5

3

2

2

1

1

1

1

206

206
1

8

8

4

4

3
1

1

1

1

3

3

4
187

1

171

1

9

1

1

85

1

1

84
1

3

3

3

3

3

7
32

6

6

18

1

4

4

4

9
8

1

9

2

1

1

4

1

1

1

1

1

1

1

3

1

1

1

3
5

2

2

11

5

5

6

2

2

1

1

20

20

12

12

8

1

4

3

65

65

1

1

64

64

64

11

11

11

1
11

10

16

16

2

2

13
1

1

1

11

1

1

7

7

1

1

6

6

5

5

5

1

4

741

741

741
63

196

10
35

25

2

89

356

10

10

10

1

9

266
59

17

2

1

1

1

6
1

2

2

3

4
9

5

29

28

2

1

1

1

4

20

1

1

161

2

2

11
157

145

1

2

2

30

4

4

22

2

2

2

2

1

1

132
8

9

7

7

2

2

2

2

11

11

9

9

2

2

20

20

3

17

14

1

1

1

1

13

13

1
13

12

33

1

32

15

15

1

1

1

3

1

1

2

2

9

8

8

1

1

1

1

9

9

8

8

8

8

8

1

1

1

1

1

1
9

8

3

3

2
3

1

5

5

4

3

1

1

1

5

5

5

5

5

5

332

10

10

1

9

25
322

4

4

4

4

4

1

1

1

1

10
70

1

1

1

6

6

6

6

5

5

5

2

2

2

2

18

1

1

1

6

6

1

4

1

11

11

5

1
5

4

4

23

23

8

15

3

5

1

5

1

4
9

1

1

3

119

23

1

1

1

1

1

1

21

21

21

11
96

19

5

5

5

1

1

13

13

1

12

40

6
25

1

1

18

8

8

6

2

7

7

7

26

12

12

1
14

12

1

27
93

13

10

10

3

3

3

2

2

2

1

1

1

1

9

8
6

1
2

1

1

1

1

1

1

16

8

8

8

8
1

6

1

1

24

24

1

21

17

4

2

1

1

1

1

3201
14

9

1

8

25

25

1

1

1

1
24

3

3

19

19

1

1

471

44
170

5

5

23
6

1

1

9

9

9

5

1

1

1

1

8

8

8

8

4

4

4

4

8

3

1

1

2

5

5

14

3

3

11
5

4

1

1

20

1

1

1

11

10

1

2

5

5

5

5

1

5

1

1

2

2

2

1

1

1

1

39

4

4

35

35

301

1

1

1

300

300

300

2

2

2

2

2

2560
8

2134
23

132

131

131

1

1

1101
6

5

1

4

4

1

1

4

4

4

4

4

5

5

93

1

92

4

1

3

3

16

7

9

9

9

3

3

7

1

6

1

11
934

8
467

5

66

1

95

2

1

1

1

277

6

3

1

204

5

122

2

3

72

144

8

1

1

1

71

65

22

43

6

9

13

4

5

5

1
717

81

81

125

125

510
3

6

11

5

1

484

10

1

1

1

7

7

2

2

2

2

1
23

9

9

13

8

5

13

1
13

11

1

4

1

3

3

111

5

4

1

100
22

6

3

45
1

2

3

1

2

6

29

1

1

5

12

6

3

2
3

1

3

3

3

418

7

1

1

6

6

232

1

1

231

215

3

9

9

4

10

2

2

8

3

5

101
2

4

4

2

2

1

1

11
10

1

72

49

23

9

9

23

23

20

1

1

1

1

1

1

45

45

45

4

3

3

1

1

55

55

50

46

46

4

4

5

5

5

5

61

21

10

1

1

9

9

11

10

10

1

29

29

19

19

1
10

9

9

11

11

11

11

16

11

11

11

11

11

4

1

1

1

1

1

67

1

1

66

9

9

9

9

9

51

51

51

51

6

6

6

2

2

4

4

1

1

14521
557746

16888
580

163
4

1

2

1

3

1

2

1

110

1

7

5

1

3

1

7

5

4

1

1

2

5
177

106

17

17

89
4

4

81

68

13

2
66

2

2

5

5

5

2

1

1

26

1

25

3

3

6

6

1
18

1

1

9

5

1

1

2

2

143

4
75

1

1

4

4

4

2

2

2

1

1

8
32

15

3

6

1

1

5

1

4

9

9

10
8

1

1

1

3

3

3

3

7

1

6

5

5

5

9

2
9

4
7

3

2
46

2

3

1

1

1

3

3

2
5

3

10
1

5

1

2

2

3

1

10

10

5
6

1

2

2

2

2

1

1

1

1

1

5185
40

3615
13

1

1

1009
3549

15

1

1

1

2

7

9

5

1

11

2

2

11

2

22

40
3

35

35

2

2

6

1

412
2

4

4

4

2

10

4

1

1

1

28

356

4

27

3
93

11

1

64

1

2

11

4

6

7

11

4

1

9

2

2

28

1

3

5

7

8

4

4

5

4

1

11

43

2

5

43

4

4

1

11

1

3

16

8

345
26

109

109
29

80

80

146

1

1

34

5
10

5

24

28

3

3

585
156

2

75

3

3

2

4

13

3

7

7

7

1

3

13

3

13

9

17

2

1

5

3

7

1

3

23

5

3

2

3

2

1

5

3

1

1

1

2

3

31

4

11

1

4

8

1

2

8

1

1

5

22

1

11

5

2

1

2

4

1

4

7

1

1

13

2

1

4

2

2

1

3

4

6

5

6

2

27

2

4

5

25

129

451

223

183

3

1

4

4

13

6

14

3

17

17

4

1

1

3

31

7

7

24

16

8

2

2

1528

7

6

1

4

4

1466
53

1

3

9

24

1

4
954

11

9

930

7
73

1

1

18

14

10

8

1

1

12

4

7

1

331

1

4

10
49

12

1

7

4

13
1

1

1

4

6

2

2

2

99

1

1

1

14
6

5

3

1

5

5

6

6

6

8

8

3
47

16

1

8
15

7

20

20

5

4

4

1

3

2

1

1

1

2

1

1

11

11
1

1

2

7

3

1

1

2

2

4

4

4

4

1
524

31

1
31

30

10

17

3

181

181

181

14

74

93

2
1

1

1

28

20

3

13

4

8

5

5

3

58

55

55

55

3

2

1

9

9

9

9

63

20
63

5

7

9

3

2

2

2

20
14

6

1

1

2
151

3

2

1

1

9

9

18

4

4

10

2

1

1

1

61
20

1

4

1

4
27

20

3

3

1

1

3

3

12

14

7

7

26

26

1

1

7

7

7

7

134

134
9

8

8

5

1

4

7

1
3

2

1

1

1

4

4

2

2

94

11
94

83

77

6

1

1

106

105

9
91

7

52

17

6

8

7

1

3

1

2

2

3

3

1

1

14

14

12

1

1

10

10

2

2

1032

1032
3

1

1

4

4

4

4

29
424

22

3

6

2

4

58
17

41

41

41

3

58

21

1

4

6

11

1

2

24

175

107

8

4

4

56

596

3

17

1

560

7

7

8

8

5746
76

4561
109

2

2

12

11

1

10

1

3
527

23

14

9

501
1

6

36

380
8

4

217

45

106

1

77

2
368

366

9
2

5

2

893

395

18

3

24

4

34

22

3

2

278

1

6

498
465

30
2

23

1

1

2

1

3

4

4

7

7

10
1383

1354
1350

4

9

10

9

1

13
6

7

9

9

5
16

7

4

14
33

3

1

2

1

15

2

2

6

6

1

1

5

5

5

16

16

16

7
1152

23

12

1

976
1086

110

14

6

3

2

2

2

14

14

14
12

2

49

18

18

2

2

29
7

6

16
6

6

1

3

168

20

14

6

1

1

140

9

1

1

2

127

7

7

4

2

1

1

2

2

2
291

32
4

1

2

25

2

231

4

1

3

227

21
2

7

7

5

3

3

10

9

1

31
407

16

14

2

2

5
17

1

7

4

4

4

148
1

5

1

45

1

79

27

52

2

13

1

1

1

25
190

1

7

16

2

17

17

4

118
3

9

12

7

16

32

8

2

3

5

19

2

16
164

25
85

1

30

29

8

5

16

9
18

2

1

1

7

11

11

24

24

10
1

9

95

20

14
4

8

2

2

6

6

6

13

13

6

7

7

10

10

20

7

7

1
3

2

7

7

7

3

3

31

1

1

6

6

1

1

3

3

18

13
11

1

1

5

2

2

1

1

1

1

5
782

194
1

8

8

8

11

11

11

1

1

1

1

2

2

8
24

1

3

3

4

6

1

1

3

3

3

9
126

18

45

3

3

1

37

1

53

1

3

5

5

9
3

6

2

2

1
2

1

1

1

1

11

1
8

1

3

1

1

2

3

3

13

13

13

510
4

98
354

13

1

11

2

3

3

7

1

2

1

95

1

7

5

3

102

6

11

59

7

3

6

1

2

7

1

1

12
8

1

1

2

3

3

15

15

1

1

3

3

14

14

4
30

1

8

3

3

12

2

71

71

1

1

2

2

14

3

3

1
8

7

3

1

1

2

6

1

1

1

5

2

2

3

4

3

3

1

1

3

3

19

19

9

10

6

6

6
2

2

2

202

12
188

25

25

25

3

3

24

24

24

6

6

7

7

21

15

15

6

33
62

13

16
7

2

7

1

1

5
2

3

12

12

10

6

4

2

1

1

12

12

12

105

7

6

1

7

1

1

3

3

1

1

2

2

11
91

29
80

11

3

1

1

1

17

3

5

5

1

1

2

2

4
113

30

1

1

3

3

3

1

1

1

3

3

1
22

18

3

3

1

1

1

5
43

9

1

1

3

5

10

1
10

8

1

1

1

1

11

11

6

3
6

3

1

1

8

8

7

7

1

1

8

8

8

4

4

4

3
15

2

10

1671
22

335
7

15

6

7

1

1

1

3

3

51
2

2

4
30

4

4

2

8

5

3

6

6

5

22

7

15

10

10

1

1

8
98

1

16
87

7

7

16

3

1

8

4

7

1

13

4

2

4

4

3

3

1

1

21
120

9

3

1

4
41

6

3

6

2

7

3

8

2

4

13

10

1

11

6

28
1295

2

2

67
9

19

8

29
7

1

12

1

1

4

3

1

1

12

12

14

2

1

1

5

5

5

9

9

100
21

2

13

6

56

8

1

3

2

3

4

34

1

2

21
4

5
14

4

4

1

1

1

1

2

2

17
7

2

6

2

457

457

70
11

4

3

2

43

43

7

3

1

3

496
121

1

1

3

86
80

5

1

1

6

218

1

1
2

1

3

8

7

7

7

38
14

24

19
8

4

7

14

14

4

4

4

10

10
5

1

3

1

1

111

1

4

11

1

94

204
6829

3

3

3

3

93
3503

28

7

1

6

6

2

2

2

3

3

3

3

3

13

13

10

10

3

6
7

1

30
556

20

20

1

1

72
398

126

25
150

9

3

5

1

3

1

1

4

1

46

9

12

1

1

4

1

10

2

8

3

3

1

1

3

3

1

10

10

1

1

1

11

3

1

1
23

6

16

8

8

12
9

3

3

61

2

1

1

13
55

1

15

3

4

1

17

1

2

11

11

6

6

6

1
26

2

2

2

8
1

7

7

11

2

7

2

4

1

3

5

5

5

1

1

1

1

19

19

8

11

298
605

12

6

7

276

1

3

2

88
810

11

5

6

6

37
650

36
19

3

3

2

2

11

1

1

5

3

3

2

572
149

1

1

6

4

5

31

2

10

19

6

102

20

7

16
126

5

6

4

25

1

1

1

14

10

8

10

2

9

1

1

4

7

1

3

10

23

11

2

24

9

1

8
59

19

17

1

1

1

32

1

22
4

18

8

1

2

1

1

1

101
8

10

10

7

3

2

2

14

14
10

4

4

4

12

12

15

14

4

10

1

36
6

21
27

2

4

3

3
136

10

2

2

8

17
69

2

9

2

26
5

4

7

3

1

5

1

13

14

1

3
8

4

1

5

1

1

32

1

1

1

5

5

1

1

33

2

2

1

1

14

14

16

16

16

90

3

3

33

4

23

23

6

1

1

30

1

29

29

7

1

6

6

2

2

14

14

14

21

3

3

3

18

18

8

8

8

112

7

6

1

1

9
83

26

3

2

2

9

7

1

24
11

1

4

1

7

1

1

13

1

12

12

8

8

419
2

3

3

12
6

2

1

1

1

1

1

1

1

352
31

53
299

13

4

1

4

15

1

7

7

2

2

8

8

137

3

1

6

4

6

8

4

1

4

11

4

2

4

1

1

1

1

1

1

12
19

7

1

1

2

2

13

13

12

12

42

19

1
23

22

8
302

14

1

1

13

38

3

1

34

49
190

1

24
84

2

1

1

11

1

5

17

9

1

1

2

9

12

23

6

1

3

11

7
52

5

5

2

7

12
3

4

3

2

2

3

5

4

17

7

7

7

10

4

4

6

9
62

7

7

5

5

4
23

7

7

7

1

1

3

8

8

10

10

1

1

1

21

3

3

6

6

12

3

2

1

9

9

9

2

2

1
2

1

21

19

2

161
4

5

7

5

8

1

1

1

1

4

4

108

3

6

2

1

84

84

23

1

3

1

7
17

3

5

2

1

1
34

1

11

4

7

1

9

15

1
6

5

5

4

3

3

9
2

6

2

4

1

1

1

1

1

2
567

549
45

13

13

6

2

1

1

1

3

3

1

1

2

2

2

2

2

2

2

1

1

1

7

1

4

2

3

3

5

5

3

2

2

2

11

6

5

13

13

3

3

3

1

1

2

2

2

1

4

5

5

4

1

6

6

6

1

1

12
10

2

1

1

18

6

2

10

4

4

52

52

7

7

3

3

3

3

3

4

4

48

2

6

16
7

1

8

9

9

6

3

3

2

2

23
1

15

11

4

7

7

1

1

6

5

5

4

4

6

6

47
10

1

1

7

1

1

1

1

19

1

1

1

1

2

5

4

4

1

7

2

2

2

3

4

1
3

2

1

2

2

57

4

1
20

1

1

16

1

2

10

14

7

16

6

2

4

4

20

1

17

1

1

4

4

1

1

1

8

8

1

1

1

1

1

1

1

6

1

5

5

4

4

4

4

4

15

6

9

7
3

4

9

9

5

5

4

17
482

1

1

17
393

1

1

1

1

1

1

1

1

45
13

8

1

3

5

2

1
13

4

6

1

1

1

1

5
17

11
8

2

1

1

10

10

42
250

2

7

1

9

2

2

1

6

10

1

1

3

1

2

3

1

1

3

38
141

1

1

1

8

11

2

2

1

5

2

6

1

1

6

2

1

7

1

1

1

3

1

22

1

1

4

1

4

2

1

1

1

3

1

3

3

1

2

2

2

3

3

3

3

42
1

11

2

1

4

1

21
13

2

3

1

2

1

71

2

1

1

27

23

2

1

1

1

5

5

2

1

1

26
4

16

9

7

7

6

6

6

9

1

1

7

518

518

517

517

1

1

1

1214
18

617
20

28
11

17

17

23

7

7

16
10

6

11

11

1

1

6

6

6

6

5

5

17

7

7

10

6

6

6

5

2

2

2

1

11

11

25
136

26

12

1

1

2

1

1

7

2

10

40
10

1

4

5

10

7

3

8

3

1

1

2

6

6

6

9
123

60

3

4

33

13

1

14

14

5

5

4

4

9

9

27

27

27

25
5

3

17

6

6

4
7

3

3

57

57

10

8

2

1

1

11

11

34

34

76
5

1

43

14

9

4

79

25
3

19

3

54

54

13

13

6

6

7

348
14

2

2

3

3

6

4

4

2

4

4

8

5

2

2

1

14
4

5

5

9

9

8

8

7

1

1

1

1

1

2

2

4

4

3

1

2

7

5

2

11

4

6

1

11

11

5
8

1

2

22

22

4

4

4

5

5

3

3

3

3

11

11

57
164

5

1

3

2

46
1

16

3

12

1

3

10

3

44

3

9

1
9

8

3

3

8

2

6

49

38

38

11

3

2

1

8

14

2
14

12

10

1

1

1

1

9

4

4

2

2

3

3

11

11

4

4

7

7

1

1

1

1

9

9

3

3

1

5

5

17

17

10

1

6

3

7

7

886

8
855

37
2

3

1

2

1

3

25

1
470

6
460

453

1

6

435

434

1

4

1

6

1

1

1

7

7

7

2

2

2

1

1

133

68

67

3

3

64

64

1

1

1

1

1

2

2

1

1

1

1

1
60

3
45

12
8

4

28

8

1

19

14

5

2

2

14
2

1
3

2

1

1

2

2

2

2

1

1

4

4

2

2

2

1

1

1

125

87

58

53

53

5

10

10

4

4

2

2

13

13

7

7

31

8

8

8

17

17

2

2

4

2

2

80

32

20

3

17

17

12
10

2

35

12

6

6

5

1

23

23

13
1

12

31

31

3

3

3

3

5

5

2

3

2

1

2

2

21

6

6

2
15

4

9

9

9

518451
3415

5
845

9
614

26

6

5

15

56
19

4

11

22

6

1

1

4

1

261

2
3

1

40
258

30
2

5

10

13

8

80

8

8

84

17

13

4

13
22

1

8

1

7

5

5

2

2

14
63

3

25

25

21

31

19

12

12

10

14

5

6

3

10

7

2

2

5

21
6

6

8
3

5

1

1

1

18

16

2

11
35

24

6
226

1

1

38

38

6
13

6

1

1

3

2

2

1

2
78

16
18

1

1

4

13

13

9

6

2

24

21

21

14

14

32
15

17

3

1

2

11

4

6

6

1

5

5

1

1

1

17

17

17
4

2
4

2

9

8058
512516

31
680

25

11

9

2

2

7
27

1

1

18

12

3

1

2

6

1

5

18
1

2

15

14

1

35

11

3

21

12

9

49

3

27

19

6

6

7

18
85

6

3

5

53
2

13

24

1

13

7
91

84

46

13

25

11

4

4

3

13

13

15

14

1

12
4

3

1

2

2

4
75

31
1

2

6

5

15

2

40

45

29
3

1

2

4

7

12

16

59

26

4

29
1

13

5

10

15

6

1

5

5

4

20
2

2

5

7

7

4

2

2

19

19

4
16

7

2

3

2

5

2630
170

19

19

11

1

10

1

3

1

5

23
60

9

6

1

20
2

9

4

3

2

1

13
18

1

4

4

1
4

3

9

3

3

4
164

2

82
131

5

1

1

19

8

3

11

1

10

12

5

1

1

1

27
222

1

1

1
63

5

2

2

1

5

1

5

2

12

16

11

58

11

6

11

9

12

1

4

5

13

42

18

24

42

21

21

21

35

27

8

8

1

1

20
4

1

6

9

2

7

9

9

76
690

184

62

356
124

2

3

9

31

3

1

2

14

1

3

35

3

3

15

5

22

7

31

3

1

8

3

5

2

2

16

2

7

5

21
6

11

2

2

2

4

2

2

14
39

1

24

9

15

301
34

45

1
3

1

1

219

4
62

12
58

2

1

18

2

3

4

1

14

1

4

4

12

12

12
1

11
2

2

7

1

8

8

7

1

1

6

33
113

4

6

9

3

1

2
24

1

1

3

8

7

2

2

7

1

23

12

7

5

5

17

17

67
6

6
22

3

5

7

1

8

1

13

9

7

1

2

2

201
1

149
30

27

33

4

9

4

3

6

6

4

1

22

11

2

9

1

15

13

3

1

2

11

11

45

39

4

7

6

12

9

1

6

2

2

153
32

1

11

3

2

2

1

101

13

11

2

2

314
2901

138
20

81
10

2

1

7

1

9

4

3

1

1

10

1

5

13

9

2

2

11

4

22

84

6

30

25

23

15

15

118
575

2

7

6

1

4

11

15

1

1

24

7

15

7

8

22

1

16

2

5

10

10

244
55

1

5

4

2

14

3

16

11

12

6

1

1

9

22

19

3

2

4

4

6

3

22

13

1

5

2

5

18

4

5

4

15

6

9

9

37

37

22

12

5

5

87
209

14

14

61
23

1

2

1

7

11

8

8

1

1

4

13

14

645
111

4

1

20

1

36
39

3

2

46

4

50
407

26

56

112

7

3

29

26

19

1

21

24

1

32

10

26

16

9

1

43

30

13

13

8

8

44

4

8

4

24

22

2

4

273
27

31

41

75

18

35

8

14

21

30

9

17

8

14

81

52

14

14

15

96
28

7

23

11

5
27

11

1

6

4

276
53

14

7

2

17

7

1

175
39

1

9

5

4

6

11

35

2

12

1

8

12

6

5

19

13
45

6

5

1

1

13

13

13

13

13

12
242

40

4

4

4

4

7

11

5

3

3

104

8

26

7

495861
239577

36
18

17

15

1

1

1

77

77
69

8

169
2757

174

16

809

211

455

452

452

203

268

3
54

30

30

1

10

10

375

375

14
384

275
232

43

95

7

7

162
32

14

3

19

1

12

11

30

6

7

3

1
21

4

2

7

1

6

3

1

1

92721
20297

135
134

1

338

1846

2169

450

474

344

4009

616

2069

5312

2558

6212

6689
314

15

1426

4929

5

2603

296

469

2944

2288

3772

4762

1844

8584

1784

4494

3566

909

888

86

14

17

55

95664
12750

18431
1044

334

2

27

512

6

2

7

2

50

14

1

36

92

304

10

10

16

16

312

563

69

1

164

15

91

6

17

39

1

2

1

37

168

432

2

1349

21

4

18

4

1

7

237

2

2

22

433

2

10

17

8

793

527

163

11

5126

10

63

467

23

19

7

287

12

71

74

6

4

1

3

19

73

2

28

2

26

299

7

13

19

34

64

6

7

111

9

54

1701

142

1

13

7

22

144

10

475

36

80

10

23

47

33

97

44

221

80

14

187

21

2

15

14

1

9

4626

833
838

5

2

326

9092
870

567
622

1

10

31

13

99

144

289
295

6

8

217

1886

61

563

255

1799

46

89

13

6

148

10

543
502

31

10

24

20

4

1026
911

1

107

7

33

186

149

2176

12733

129

449
2561

6

21

15

59

10

6

99

26

1068
1380

7

29

10

6

2

233

2

1

5

11

6

185
198

13

25

13

2

252
186

2

55

9

8028

935

22770

267
255

12

3

3

57825
9634

109

97

487

416

224

249

148

33

140

233

255

386

895

6190

323

115

230

72

146

169
123

46

53

192

308

160

659
647

12

201

93

18

178

334

135

163

51
37

14

613

96

199

75

104

3344
9453

6109

183

194

67

4666

44

307

81

928

195

159

62

223

231
260

29

61

162

335

57

251

1010

354

254
222

32

240

613

203

463
9696

77

50

351

32

197

376

50

388

224

32

703

186

593

386

9

358

424

26

126

48

28

71

43

27

169

146

1049

689

31

62

25

174

21

21

102

235

50

96

50

140

211

29

60

584

131

353

3

72

239

376
385

9

492

32

382

171

112

133

7

497

821

238

31

29

29

2

2

2

2

694
20

402

272

2051
841

10

38
34

4

93
87

6

1

1

74

73
489

7

2

15

31

9

36

9

3

47

3

6

82

39

24

7

51

1

40

1

3

22

28

137

61

39

207

10

326
753

164

20

40

5

25

11

47

16

28
64

36

14

7

7

161
164

1

2

4

10

9

3

3

6

29

29

1147
2563

1

73

50

85

8

23

32

32

13

30

99

22

214

16

7

16

89

18

56

34

13

30

19

20

249
3

16

106

90

3

6

25

74

29

64

2099
156

10

10

7
18

11

27
16

2

3
1

2

6

44

2

42

12

12

123
13

1

12

7

78

8

24

1

45

1

1

10

368
92

11

10

11

4

38

5

37

7

1

7

2

107
4

1

20

7

9

65

1

3

3

7

1

16

6

102
2

2

59

39

2

37

62

23

8

31

12

19

13

13

8
36

16

12

3
66

11

11

26

26

9

3

6

52
620

38

28

10

4

9

115
11

24

62

14

4

23

16

26

2

172

12

3

51

5

5

5

2

13

6

23

113

22

91

91

16

16

92
1

13

78

13

13

4

4

4

112
10

19
9

10

30

24

25

4

19

15

15

4

5

5

3

3

51
24

13
8

5

2

12

3

5

4

5

5

115

17
2

1

2

8

4

6
1

2

3

3

6

6

6

2

2

83
27

4

52
4

11

10

1

17

5

4

1

1

36
538

96

2

2

94

3

74

3

4

37

9

21

17

5
211

121
34

4

8

49

10

13

1

1

18

6

17

8

1

17

17

4
33

10
5

1

4

18

1

35

27

8

10

5

4

5

3

12
1

2

4

5

4
182

5
2

3

3

3

3

3

2

2

1

2

37

36

1

3

3

5

5

4

4

53

17

31

5

3
63

3

9

29

2

4

4

8

1

1

1

1

421
79

8

192

1

1

1

1

4

3

1

11

1

2

1

7

3

1

17

1

3

4

3

4

6

16

2

9

5

4

6

19

2

3

22
519

17
11

2

2

2

36

5

5

21

3

7

7

11

10

10

109
10

2
16

1

13

12

1

6

1

1

1

4

10

1

8

1

3

3

3

7

7

7

1

1

56

56

67

8

2

6

6

9

3

6

3
5

2

2

45
5

40

91

80
5

56

56

1

7

5

6

1

1

6
1

5

5

4

4

28

2

2

26

1
8

5

1

1

18

3

3

3

146

7

7

78
26

10

8
24

8

8

18

10
61

1

1

3

2

2
17

1

1

8

2

2

1

5

7

2

6

7

65

65

3

3

1

1

1

1

1

1

4
11

6

1

30

30

12
17

5

13

10
13

3

1

2

1

1

1

192

1
8

7

5

5

5

5
3

2

2

2

1

1

1

1

3

3

1

2

3
87

5

2

2

1

1

1

1

2

2

1

1

1

1

1

1

16

16

16
1

3

3

1

1

1

8

8

3

2

1

63
4

1

57

1

8

8

8

2

2

2

2

2

2

84

5

5

5

5

5

5

2

1

1

1

1

1

1

1
51

9

9

3

3

5

1

1

16

2

1

1

1

1

14

1

1

3

5

4

1

4

4

4

1

1

22

22
4

1

1

1

1

1

12

12

3

3

1

1

3

3

1

1

1

1

1

1

26

1

18

1

1

5

1

1

1

1

1

1

1

15

11

11

7

4

4

4

4

4

213
39

1

41

2

2

2

1

5

30

1

7

1

1

3

6

1

70

74

74

1

1

1

6

6

6

22

22
12

10

23

23

1

1

1

1

1

15

15

3

2

1

1

8
12

4

4

1

1

1

3

3

1

1

1

1

1

1

3

3

3

1

1

1

26

7
1

2

1

3

18

16

16

16
3

4

9

2

2

1

1

1

1

1

80

9

1

10

60

4

4

4

4

4

3

3

1

1

1

1

1

1

1

1

1

2

2

2

1

1

1

1

369
6396

6

6

6

4

4

4

4

4

2

2

2

2

3980
1

3975
165

16
1382

1247

506

501

501

6

1
6

5

2

2

2

2

478
4

136

136
25

96

2
1

1

13

1
338

4

4

325
1

25
323

5

293

1

8

5

2

1

1

1

1

14

14
13

1

1

3

3

2
1

1

1

1

1

1

1

2

2

2

2

2

656

1
656

581

581
1

2
484

6

6

194
278

59

25

198

96

96

74

1

1

73

73

73

85

85

85

85
17

1

1

1

1

1

1

2

1

1

43

1

1

1

41

19
8

1

2

8

1

1

1

3

3

3

3

3

31

13

1

1

3

3

9

8

1

18

18

18

18

75

75

21
1

1

1

1

1
7

2

2

1

1

1

1

4

4

4

1

1

1

1

2

2

1

1

1

1

8

8

2
8

3

3

1

1

1

47

47

47
41

6

1

5

7

7

7

7

7

7

1

1

1

1

1

1

1

1

9

5

1

1

1

1

1

1

4

4

4

4

4

4

4

2

2

2

2

1

1

2

2

2

1

1

1

1

1

37
2428

1
614

33

33

33

33

33
3

6

22

2

1
418

5
226

73

1
69

5

5

1

1

2

2

1

1

1

4

1

1

1

3

3

4

4

1

2

1

5

5

2

3

3

2

2

2

2

2

2

2

2

1

1

1

1

1

1

3

1

1

2

2

37

37

37

37

2

2

2

1

1

1

1

1

1

1

4

2

2

2

1

1

1

1

1

1

1

1

1

1

1

1

1

1

1

1

1

1

1

1

147

4

4

4

4

2

2

2

2

2

71

9

1

1

1

1

1
7

6

6

62

62

62

62

1
6

3

3

3

1

1

1

1

1

1

1

2

2

2

2

1

1

62

13

13

13

13

47

47

28
47

19

19

2

2

2

105

105

105

105

104

104

1

1

86

12

12

12

12

74

74

74

2

66

66

6

34

9

1

1

1

1

8

8

7

7

7

1

1

24
1

14

14

14

14

9

9

6
9

3

1

1

1

1

1

128

115

94
115

14

5
14

2

2

2

2

5

5
4

1

6

6

5

5

1

1

1

1

1

1

13

12

12

12
1

1

4

6

1

1

1777

7

1

1

1

1

1

1

5

5

5

5

1

1

1

1

1

1

1770
4

3
1714

3

3

1
3

1

1

1

1

1

1

1

1

1

1

1707
25

115

115
2

21

8

8

8
1

7

2

1

1

1

1

1

1

1

1

1

1

10

7

1

1

1

5

4

1

3

2

1

1

1

1

14

1

1

1

1

2

2

2

2

2

3

3

3

3

1

1

1

1

7

7

5

5

5

1

1

1

1

1

78

3
77

1

1
5

3

1

1

1

1

1

1

1

1

1

1

1

1

1

66

66

66

66

66

1

1

1

1

1

2

2

2

2

2

2

2

275

143

2
17

4

2

1

1

1

1

1

1

1

3

3

3

1

1

1

1

1

1

1

1

1

1

2

2

1

1

1

3

2

2

1

1

115

115

114

114

1

1

11

2

2

2

2

9

1
9

8

2

1

4

1

132

93

1

1

1

1

11

11

1
11

5

1

1

4
1

1

2

2
81

1

1

1

5

5

3

2

2

1

1

1

1

56

17
3

9
5

3

1

1

1

1

1

1

1
3

1

1

39

39

1
16

12

12

12

2

2

2

1

1

1

28

2

1

1

1

1

1

1

1

23

1

1

1

1

1

22

22

21

1

1

1

1

1

1

2

2

1

1

1

1

1

1

11

11

1

1

1

1

9

9

9

1

1

1

2
1290

1
4

3

2

2

2

1

1

1

1

7
1284

4

2

2

2

2

1

1

1

1

1

1

1273
10

2

2

1
2

1

1258
40

1155
86

2

2

4

1

1

3

3

991

3

3

23

2

5

2

2

1
2

1

2

8

4

1

3

3

3

1

63
20

2

2

3

3

1

4

2

1

1

1

1

1

1

1

1

3

1

1
2

1

1

1

3

1

1

1

1

1

1

1

1

1

2

1

1

1

2

1

7

3

3

1

2

1

3

1

1

1

2

1

1

1

1

1

52

52

23
52

1

1

1

5

1

1

1

1

1

1

1

1

1

1

1

1

1

1

1

1

1

1

1

1

2

2

2

13

4

3

1

1

1

1

8

8

8

4

2

2

2

2

1

1

1

4

4

4

3

3

3

1

1

21

16

6

6

6

3
1

1

1

1

1

1

1

3

3

3

10

1

1

1

1

1

9

9

9

9

9

9

4

4

4

4

4
1

1

2

1

1

1

1

1

1079

1079
74

16

8

6
8

2

2

8

8

8

8

8

27

27

27

27

7

20

141

141
5

25

6

6

19
6

7

6

6

6

111

4
111

94

94

13

32

32

32

8

5

3

24

23

1

92

5

5

5

5

5

64
10

51
2

43

28
43

2

4

7

2

6

6

6

3

3

3

3

2
23

1

1

1

1

1

1

1

1

1

19

14

1

1

1

1

13

13

6

7

7

4

4

4

1

1

1

1

4

4

4

4

4

3

3

3

3

3

3

28

28

28

28

14

14

14

1

1

13

13

13

11
648

241
21

38

4

4

4

34

1

1

33

33

33

40

40

26

26

26

14

4

10

138

3
138

5

5

2

2

4

4

2

2

2

21

21

101
6

9

9

86
2

8

67

67

9

4

4

4

4

3
396

5

5

5

5

387
9

319
13

1

1

1

7

7

7

259

256

11

225

20

2

18

3

3

9

9

9

12
1

3

3

8

8

8

2

2

2

16

16

16

59
4

4

4

4

17

1

1

16

16

29

29

2
29

12

15

5

5

5

1

1

1

1

1

1

1

1

1

744
1

12

8

8

8

8

8

8

3

3

1

1

1

1

1

1

2
584

8

4

3

3

3

3

2

1

1

1

1

1

1

4

4

4

4

4

3
544

26

1
26

23
2

11

2

3

5

1

1

1

1

506

506

18
4

2

2

1

4

3

2

487

487

1

1

2

2

2

2

2

7

7

3

1

2

4

4

30

10

6

1
6

3

3

1

1

1

1

1

1

1

2

1

1

1

1

1

1

1

4

4

4

4

4

4

4

15

14

8

2

2

2

6

6
5

1

1

1

1

1

1

5

5

5

5

1

5

5

5
4

1

1

147

65
1

4

4

4

4

4

4

60

58

58

58

58

58

58

58

2

1
2

1

1

1

1

1

69

14

9

9

9

5

1

1

4

4

2

2
1

1

2
1

1

1

5

5

5

5

5

55

52

52

52
1

1

1

50

50

50

1

1

1

1

2

2

2

5

1

1

1

4

4

4

4

4

4

4

4

1
4

1

1

2

4

3

3

3

3

1

1

1

1

2

2

2

2

2

2

60

60

60

60

60

60

45
1

32

2

2

2

2

23

23

8

2

2

2

6

2

1
2

1

4

4

4

15

6

6

6

9

1

1

8

8

7

7

7

7

7

12

10

10

10

1
10

9

9

2

1

1

1

1

1

8

1

1

1

1

7

7

7

6

6

1

2
81

1

1

1

1

1

65
2

1

1

4

4

4

4

4

4

58
11

2

2

1

1

1

1

43
3

38

8

8

8

4

4

2

2

2

26

1

1

1

1

1

1

2

2

2

2

13

5

5

5

5

7

1
7

2

2

3

3

3

1

1

1

1

1

1

115

102

1

1

1

1

1

1

1

1

101

101

101

101

1

1

1

90

52

52

3

3

3

3

17

17

9

5

4

1

4

5

1

1

3

3

1

1

1

10

10
9

1

10

10

10

10

10

10

1

1

9
2

1

2

1

1

1

1

1

1

1

1

1

1

1

1

1

1

1

1

1

1

1

1

1

1

1

1401821
